# Osteoarthritis, osteoarthritis treatment and risk of incident dementia: a prospective cohort study based on UK Biobank

**DOI:** 10.1093/ageing/afae167

**Published:** 2024-08-07

**Authors:** Rong Guo, Ya-Nan Ou, Li-Yun Ma, Lian Tang, Liu Yang, Jian-Feng Feng, Wei Cheng, Lan Tan, Jin-Tai Yu

**Affiliations:** Department of Neurology, Qingdao Municipal Hospital, Qingdao University, Qingdao, China; Department of Neurology, Qingdao Municipal Hospital, Qingdao University, Qingdao, China; Department of Neurology, Qingdao Municipal Hospital, Qingdao University, Qingdao, China; Department of Neurology, Qingdao Municipal Hospital, Qingdao University, Qingdao, China; Department of Neurology and Institute of Neurology, Huashan Hospital, State Key Laboratory of Medical Neurobiology and MOE Frontiers Center for Brain Science, Shanghai Medical College, Fudan University, National Center for Neurological Disorders, Shanghai, China; Institute of Science and Technology for Brain-Inspired Intelligence, Fudan University, Shanghai, China; Key Laboratory of Computational Neuroscience and Brain-Inspired Intelligence (Fudan University), Ministry of Education, Shanghai, China; Fudan ISTBI—ZJNU Algorithm Centre for Brain-Inspired Intelligence, Zhejiang Normal University, Jinhua, China; MOE Frontiers Center for Brain Science, Fudan University, Shanghai, China; Zhangjiang Fudan International Innovation Center, Shanghai, China; Department of Neurology and Institute of Neurology, Huashan Hospital, State Key Laboratory of Medical Neurobiology and MOE Frontiers Center for Brain Science, Shanghai Medical College, Fudan University, National Center for Neurological Disorders, Shanghai, China; Institute of Science and Technology for Brain-Inspired Intelligence, Fudan University, Shanghai, China; Key Laboratory of Computational Neuroscience and Brain-Inspired Intelligence (Fudan University), Ministry of Education, Shanghai, China; Fudan ISTBI—ZJNU Algorithm Centre for Brain-Inspired Intelligence, Zhejiang Normal University, Jinhua, China; Department of Neurology, Qingdao Municipal Hospital, Qingdao University, Qingdao, China; Department of Neurology and Institute of Neurology, Huashan Hospital, State Key Laboratory of Medical Neurobiology and MOE Frontiers Center for Brain Science, Shanghai Medical College, Fudan University, National Center for Neurological Disorders, Shanghai, China

**Keywords:** osteoarthritis, osteoarthritis treatment, dementia, brain structure, older people

## Abstract

**Background:**

We aimed to investigate the association between OA and treatment with dementia risk and structural brain abnormalities.

**Methods:**

We recruited a total of 466,460 individuals from the UK Biobank to investigate the impact of OA on the incidence of dementia. Among the total population, there were 63,081 participants diagnosed with OA. We subsequently categorised the OA patients into medication and surgery groups based on treatment routes. Cox regression models explored the associations between OA/OA treatment and dementia risk, with the results represented as hazard ratios (HRs) and 95% confidence intervals (95% CI). Linear regression models assessed the associations of OA/OA therapy with alterations in cortical structure.

**Results:**

During an average of 11.90 (± 1.01) years of follow-up, 5,627 individuals were diagnosed with all-cause dementia (ACD), including 2,438 AD (Alzheimer’s disease), and 1,312 VaD (vascular dementia) cases. Results revealed that OA was associated with the elevated risk of ACD (HR: 1.116; 95% CI: 1.039–1.199) and AD (HR: 1.127; 95% CI: 1.013–1.254). OA therapy lowered the risk of dementia in both medication group (HR: 0.746; 95% CI: 0.652–0.854) and surgery group (HR: 0.841; 95% CI: 0.736–0.960). OA was negatively associated with cortical area, especially precentral, postcentral and temporal regions.

**Conclusions:**

Osteoarthritis increased the likelihood of developing dementia, and had an association with regional brain atrophy. OA treatment lowered the dementia risk. OA is a promising modifiable risk factor for dementia.

## Key Points

- Osteoarthritis is associated with an increased risk of dementia, especially Alzheimer’s disease.
- Medications lower dementia risk, prominently nonsteroidal anti-inflammatory drug and opioids.
- Joint replacements reduce dementia risk, notably knee replacement has a stronger effect.
- Osteoarthritis causes reduction in cortical areas at baseline.

## Introduction

Dementia is an age-related disease characterised by a gradual decline in cognitive capacities [1]. It has imposed heavy burden on patients with their caregivers and the national healthcare system [2]with no effective measures to cure or slow its progression. Hence, management of its modifiable risk factors for prevention is essential [3].

A growing number of studies demonstrated that OA raised the risks of cognitive impairment and dementia [4–6], with a possibility via pathway of local inflammatory cytokines [7, 8]. Long-term exposure to pain in patients with OA has also been found to cause the occurrence of dementia [9–11]. Distinct and effective therapies, principally surgical and medicinal treatments, have been developed for osteoarthritis [12, 13], and possibly to decrease indirectly the rates of subsequent dementia [14]. However, prior studies to investigate whether medication reduced the risk of subsequent dementia in OA patients yielded inconsistent outcomes, some of which were negative [15–17], positive [18, 19] and neutral [20–22]. Considering the improved efficacy after OA treatment [23], so if medication or surgery reverts the heightened risk of dementia caused by osteoarthritis, it may be one of the risky elements for dementia that is susceptible to modification.

Inconsistent structural brain changes after different OA treatments (including surgeries and medications) were observed [24–26], while the mechanisms underlying the associations between OA and brain imaging measures are still unclear [27, 28]. Therefore another intention of this study was to identify the brain regions related to OA and treatment, contributing to a clearer appreciation of the potential mechanisms of dementia.

Utilising the massive sample size and longitudinal tracking over long periods of the UK Biobank (UKB), we systematically and comprehensively examine the associations of OA/OA treatment and dementia subtypes, and brain structures to extensively investigate the hidden linkages. We hypothesise that the risk of dementia is elevated by OA, and would be lowered by OA treatments. We also speculated that OA patients will carry greater risks of structural atrophy in the cortical regions based on previous literature.

## Methods

### Study population

**Figure 1:**
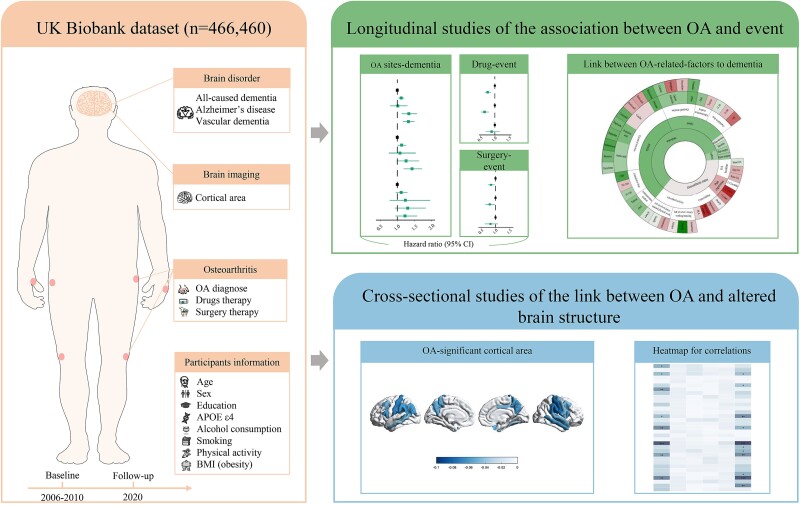
Structural summary of study. The research overview summarises the selection and analysis process of the analysis population. Abbreviations: OA, osteoarthritis; BMI, body mass index.

UKB (https://www.ukbiobank.ac.uk/) is a large nationwide prospective cohort study that enrolled 502,494 individuals. We recruited 466,460 participants between 2006 and 2010 from the UKB database and followed up these participants until the onset of dementia or the endpoint date (31 December 2020) (Fig. 1).

### Exposure to OA

OA was ascertained based on primary care, hospitalisation, self-report, death or hospital inpatient data defined by International Classification of Diseases, 10th edition (ICD-10) codes M15-M19 [29]. We conducted a subgroup analysis by categorising participants into hand OA (ICD-10: M15, M18), knee OA (ICD-10: M17) and hip OA (ICD-10: M16) subgroups. We defined participants without OA at baseline to be controls. Additionally, we took into consideration the influencing factors of OA, such as history of joint injury, manual labor and pain experience, and also explored their associations with dementia risk.

### Exposure to OA treatments

Treatment information included records of drug prescriptions and surgery history. We sorted medicine via the Read Codes, British National Formulary codes and Dictionary of Medicines and Devices codes. Surgery was defined as patients underwent joint replacement with OPCS4 code. We defined the participants who received treatment after OA diagnosis as the medication or surgery group and others who never took medication since developed OA as the unmedicated or no-surgery group. The treatment period defined from the start of OA treatments to the end of follow-up. Subsequently, in order to avoid reverse causation and insufficient treatment exposure [30], we further excluded participants who started receiving therapy in 2 years prior to the end point of follow-up.

### Dementia outcomes

Diagnosis of dementia is detailed in the Supplementary Methods section. Incident dementia events were diagnosed after enrollment.

### Covariates

Covariates are described in the Supplementary Methods.

### Neuroimaging data

The magnetic resonance imaging analysed in this study consisted of 66 cortical regions. See the Supplementary Methods for more details.

### Statistical analyses

Continuous baseline variables were expressed as mean (standard deviation [SD]) or median (interquartile range), and categorical values were present as number (percentage). We examined these variables using analysis of variance or the Mann–Whitney U test.

Multivariate Cox proportional hazards regression explored the associations between OA/OA treatments and dementia risk, with results presented as hazard ratios (HRs) and 95% confidence interval (95% CIs). Independent analyses firstly adopted a minimal-adjustment Cox model with age and gender as covariates (Model 1). Model 2 additionally made adjustments for *APOE* ε4, ethnic, education, smoking status, alcohol consumption, BMI and TPA.

To examine whether dementia risk varied with different kinds of outcomes or exposures, we conducted a secondary analysis. To further explore the different effects on dementia risk across different types of OA drugs, we subdivided the medication group into subgroups of nonsteroidal anti-inflammatory drugs (NSAIDs), opioids, glucosamine and corticosteroids. NSAIDs and opioids were further stratified by chemical structure and product name based on the World Health Organization’s the Anatomical Therapeutic Chemical codes. We calculated the cumulative use of NSAIDs and opioids using ‘defined daily doses’, which was divided into four mutually exclusive groups to generating categorical variables over time [31]. Joint replacement is further subdivided mainly into knee replacement and hip replacement.

We used the interactive terms for age, early or late-onset dementia type [31], gender, *APOE* ε4 status, length of OA, BMI [32] and TPA to assess whether there was stratification effect among the distinct subgroups (*P* < 0.1). We conducted four sensitivity analyses to test the robustness. More detailed processes were shown in the Supplementary Statistical analyses.

We investigated the association between OA and brain morphometry using a linear regression model. The *P* values for brain structures were adjusted via the false discovery rate (FDR) correction method. Adjusted significance thresholds were two-sided *P* < 0.05. Analyses were conducted by using the R survival packages with version 4.1.2.

## Results

### Participant characteristics

Our study comprised 466,460 participants with a mean age at recruitment of 56.74 (SD 8.08), of whom 254,909 (54.6%) were males. There was a total of 63,081 OA patients, including 4087 (6.5%) with hand OA, 6860 (10.9%) with hip OA and 14,155 (22.4%) with knee OA. After a median follow-up of 11.90 (SD 1.01) years, 5627 developed all-cause dementia [ACD, including 2438 Alzheimer’s disease (AD) and 1312 vascular dementia (VaD)]. In terms of different treatment routes, 17,734 OA patients underwent joint replacement surgeries and 17,856 OA patients received pharmacological treatment. Characteristics of the participants, both clinical and demographic, were shown in Table 1.

**Table 1:** Demographic and clinical characteristics according to osteoarthritis conditions

Variables	Group without osteoarthritis (*n* = 403,379)	Group with osteoarthritis (*n* = 63,081)	*P* value
Age at baseline, years, mean(SD)	56.12(8.14)	60.70(6.37)	<0.001
Gender, *n* (%)			
Female	186,832(46.3)	24,719(39.2)	<0.001
Male	216,547(53.7)	38,362(60.8)	<0.001
Education, *n* (%)			
With college degree	190,014(47.1)	23,912(37.9)	<0.001
Without college degree	213,365(52.9)	39,169(62.1)	<0.001
*APOE* $\boldsymbol{\varepsilon}$ 4 carrier, *n* (%)			
Carrier	98,511(28.7)	15,663(28.1)	<0.001
Non-carrier	244,223(71.3)	40,147(71.9)	<0.001
Ethnicity, *n* (%)			
White	354,391(87.9)	57,464(91.1)	<0.001
Mixed	451(0.1)	80(0.1)	<0.001
Asian	13,281(3.5)	1374(2.3)	<0.001
Black	10,745(2.8)	1552(2.6)	<0.001
BMI, (kg/m), *n* (%)			
<18.5	2229(0.6)	176(0.3)	<0.001
18.5–24.9	134,627(33.6)	13,551(21.6)	<0.001
25–29.9	171,210(42.7)	25,783(41.2)	<0.001
≥30	92,823(23.2)	23,134(36.9)	<0.001
Smoking status, *n* (%)			
Never	220,190(54.9)	31,242(49.9)	<0.001
Previous	137,307(34.2)	25,172(40.2)	<0.001
Current	43,562(10.9)	6231(9.9)	<0.001
Alcohol drinking status, *n* (%)			
Never	17,600(4.4)	3379(5.4)	<0.001
Previous	14,036(3.5)	3206(5.1)	<0.001
Current	370,441(92.1)	56,277(89.5)	<0.001
Depressive status, *n* (%)			
Yes	90,853(23.7)	15,673(26.3)	<0.001
No	293,212(76.3)	44,002(73.7)	<0.001
Total physical activity, *n* (%)			
<600	92,651(24.3)	16,478(27.9)	<0.001
600–2,999	183,409(48.1)	26,026(44.1)	<0.001
≥3,000	105,555(27.7)	16,460(27.9)	<0.001
Dementia types, *n* (%)			
All-caused dementia			
Yes	4330(1.1)	1297(2.1)	<0.001
No	399,049(98.9)	61,784(97.9)	<0.001
Alzheimer’s disease			
Yes	1862(0.5)	576(0.9)	<0.001
No	399,049(98.9)	61,784(97.9)	<0.001
Vascular dementia			
Yes	998(0.2)	314(0.5)	<0.001
No	399,049(98.9)	61,784(97.9)	<0.001
Osteoarthritis types, n (%)			
Hand OA	NA^a^	4087(6.5)	<0.001
Hip OA	NA^a^	6860(10.9)	<0.001
Knee OA	NA^a^	14,155(22.4)	<0.001
Only hand OA	NA^a^	1,527(2.4)	<0.001
Only hip OA	NA^a^	2,845(4.5)	<0.001
Only knee OA	NA^a^	7,346(11.6)	<0.001
Hand hip OA	NA^a^	95(0.2)	<0.001
Hand knee OA	NA^a^	201(0.3)	<0.001
Hip knee OA	NA^a^	253(0.4)	<0.001
Hand hip knee OA	NA^a^	23(0.0)	<0.001
Osteoarthritis duration, years, mean (SD)	NA^a^	17.55(9.38)	<0.001
Treatment			
Drug therapy, *n* (%)			
No	NA^a^	34,033(54.0)	<0.001
Yes	NA^a^	17,823(28.3)	<0.001
NSAID	NA^a^	11,445(18.1)	<0.001
Opioid	NA^a^	12,431(19.7)	<0.001
Glucosamine	NA^a^	1291(0.2)	<0.001
Corticosteroid	NA^a^	4792(7.6)	<0.001
Surgery therapy, *n* (%)			
No	NA^a^	44,973(71.3)	<0.001
Yes	NA^a^	17,708(28.1)	<0.001
Hip surgery	NA^a^	7,617(12.1)	<0.001
Knee surgery	NA^a^	8,672(13.7)	<0.001

Data are presented as *n* (%) and mean (SD). The *P* values are derived using Student’s *t* test, Mann–Whitney U test or $\chi$^2^ test among diagnosed with OA or without OA group.

Abbreviations: BMI, body mass index; APOEε4, apolipoprotein E4; TPA, total physical activities; SD, standardised deviation; NSAID, nonsteroidal anti-inflammatory drug; NA, not applicable.

a The untreated group and treated group were only classified in participants with osteoarthritis.

### Associations between OA and dementia risk

**Figure 2:**
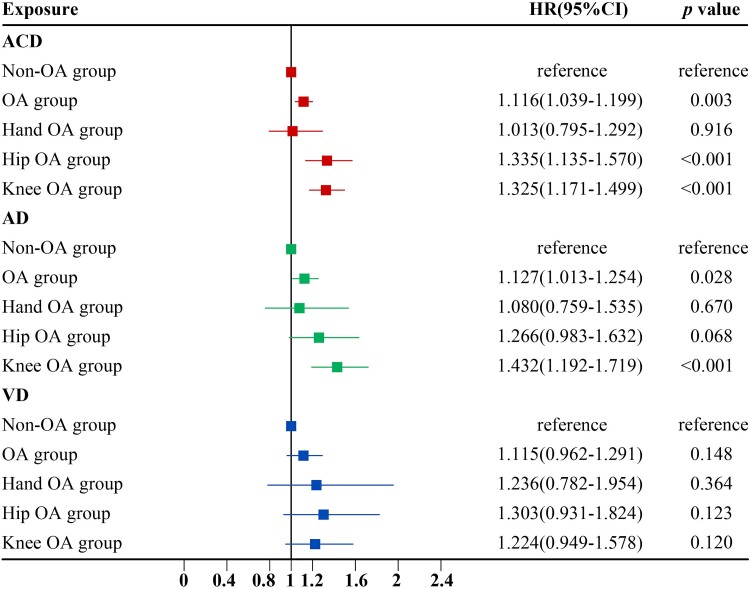
Association of OA and OA treatment with incident dementia during follow-up in the fully-adjusted model. The red, green and blue squares represent the HR of osteoarthritis to ACD, AD and VD in three outcomes, where the non-OA group is used as reference. The red, blue and green horizontal lines indicate the corresponding 95% CIs around the HRs. HRs were calculated using Cox proportional hazards regression analysis after adjustments for age, sex, ethnic, education, BMI, TPA, smoking status, alcohol status and *APOE* ε4 status. Abbreviations: ACD, all-cause dementia; AD, Alzheimer’s disease; VD, vascular dementia; HR: hazard ratio; BMI, body mass index; APOE, apolipoprotein E; TPA, total physical activities.

At baseline, 63,081 participants were reported with a diagnosis of OA, of which 1,297 developed ACD during the follow-up. Multivariable Cox proportional hazards models revealed OA was significantly associated with elevated risk of ACD (HR: 1.116; 95% CI: 1.039–1.199) and AD (HR: 1.127; 95% CI: 1.013–1.254) (Fig. 2). Concerning the different sites, we found that knee OA significantly elevated the risk of ACD and AD, and hip OA markedly added the elevated ACD risk (Supplementary Table 1). To account for the cumulative effect of the number of OA sites, we further subdivided the different sites into joints [33]. When only one joint was affected, single knee OA and single hip OA were associated with a meaningful risk of dementia. When multiple joints were simultaneously involved, knee and hip double joint OA was associated with dementia (HR: 2.197; 95%CI: 1.180–4.091; Supplementary Table 1). This is consistent with the results of the primary analysis.

The observed associations were more pronounced in older, male, early-onset dementia, *APOE* ε4 carrier, OA duration >5 years, BMI ≥ 30 and TPA ≥ 3000 subgroups (Supplementary Tables 3–9). The sensitivity analysis by excluding participants with OA diagnosed from self-reported sources and inflammatory arthritis showed the associations of OA with ACD and AD became more pronounced (Supplementary Tables 10 and 11). Even considering potential selection bias and the competing risk of all-cause mortality, the results remained robust (Supplementary Tables 12 and 13).

### Associations between influencing factors of OA and dementia risk

**Figure 3:**
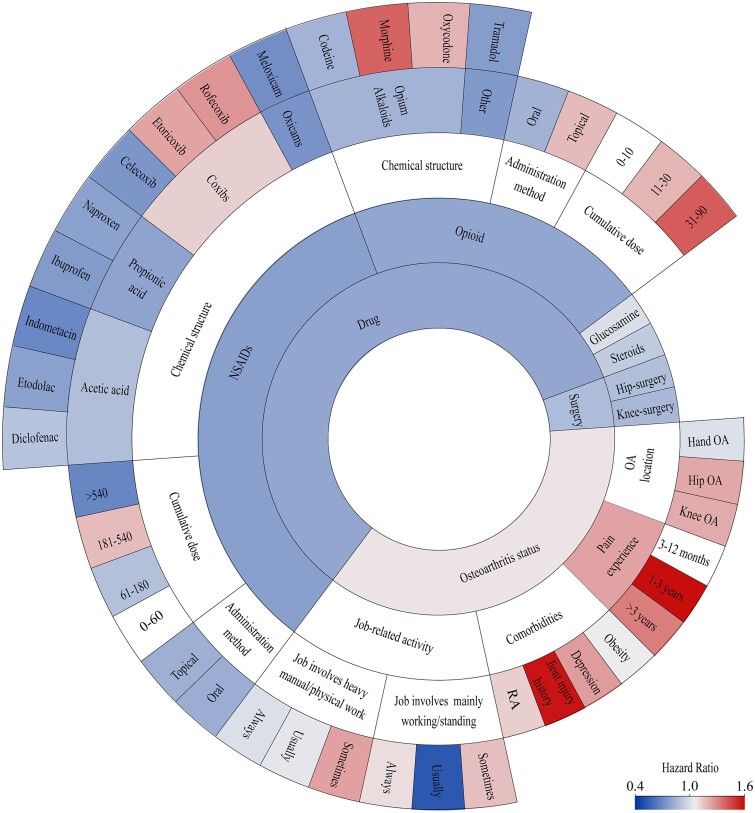
Association between different aspects of osteoarthritis and dementia. Association between different factors of osteoarthritis and dementia risk. The colour of the circle represents the magnitude of HRs, derived from fully adjusted Cox models (adjusted for age, sex, ethnic, education, BMI, TPA, smoking status, alcohol status and *APOE* ε4 status). Abbreviations: BMI, body mass index; APOE, apolipoprotein E; TPA, total physical activities; RA, rheumatoid arthritis; NSAID, nonsteroidal anti-inflammatory drug.

Many factors influence the relationship between OA and dementia, so we have performed a lot of exploratory analyses (see the grey semi-circle named ‘Osteoarthritic status’ in Fig. 3). OA patients with depression (HR: 1.382) and joint injury history (HR: 1.985) showed an elevated risk of dementia. OA patients with obesity showed no significant differences in dementia risk than those without. No disparity of dementia risk was observed between OA patients with and without chronic pain and various pain durations (Supplementary Table 14). Job involving mainly walking/standing or heavy manual/physical work reportedly among OA patients did not elevate the risk of dementia.

#### Associations between OA medications and dementia risk

**Figure 4:**
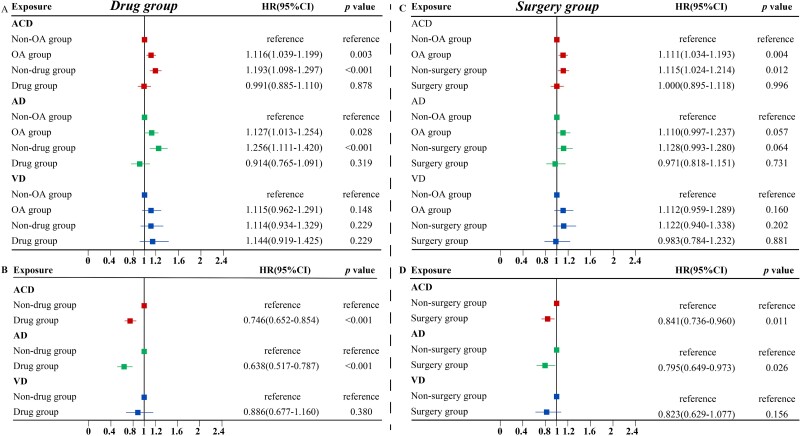
Association of osteoarthritis treatment with incident dementia during follow-up in the fully-adjusted model. (A) displays the association between medication taking and dementia in OA patients compared to those without OA. (B) demonstrates among OA patients, the incidence of dementia in drug group compared to the non-drug group. (C) shows the association between undergoing surgery and dementia in patients with OA compared to non-OA. The plot 4D illustrates among OA patients, the risk of dementia in operative group compared to the non-surgery group. Abbreviations: OA, osteoarthritis; ACD, all-cause dementia; AD, Alzheimer’s disease; VD, vascular dementia; HR: hazard ratio.

A total of 17,856 individuals with OA received medication. No significant differences in the dementia risk between medication group and healthy controls (Fig. 4A). The protective effect of medication on dementia risk was stronger after excluding 33 patients who started taking medication within two years before the outcome event (HR: 0.746; 95% CI: 0.652–0.854) (Fig. 4B).

In subgroup analyses, the stronger protective effects of OA medications against dementia can be seen in older, late-onset dementia, male and *APOE* ε4 carrier subgroups. Analyses of dementia subtypes revealed a significant association between OA medications and the risk of AD (Supplementary Table 15). The results of several sensitivity analyses remained robust. (Supplementary Tables 10, 11 and 13).

Oral medications offer stronger protection against dementia (HR: 0.747; 95% CI:0.649–0.859) (Supplementary Table 15). The protective role of NSAIDs and opioid was stronger than that of glucosamine and intra-articular steroid hormone injections. In the subgroups of NSAIDs, propionates (chemical structure) were protective against dementia risk, and ibuprofen and naproxen (product name) within the propionate group were significant. Ibuprofen was associated with AD risk, not VD (Supplementary Table 16). For opioid, protective effects against ACD and AD were observed for codeine and tramadol in the grouping of product name (Supplementary Table 17). Different cumulative exposure subgroups for oral NSAIDs and opioids were not statistically associated with dementia risk and its subtypes (Supplementary Figs 1 and 2).

### Associations between OA surgeries and dementia risk

Among the baseline OA participants, 17,734 underwent surgeries who had no evidently elevated risk of dementia during the follow-up compared with non-OA controls (Fig. 4C). Within the OA participants, after excluding the 26 who had surgery within 2 years prior to the endpoint, we found the protective role of OA surgeries against dementia remained significant (HR: 0.841; 95% CI: 0.736–0.960) (Fig. 4D).

The protective associations between OA surgeries and dementia risk were still significant in older, male, TPA ≥ 3,000 and BMI ≥30 subgroups (Supplementary Tables 3, 5, 8 and 9). As for dementia subtypes, OA surgeries showed significant associations with the risk of AD (HR: 0.795; 95% CI: 0.649–0.973). In subgroup analyses of surgical procedures, only knee replacement was significantly associated with dementia risk after correction for multiple factors by model 2 (HR: 0.825; 95% CI: 0.695–0.979) (Supplementary Table 18). Yet, no statistically significant association between surgery and dementia risk could be seen in several sensitivity analyses (Supplementary Tables 10, 11 and 13).

### Associations between OA/OA treatments and brain structures

The data on structural brain changes are based on the population that underwent magnetic resonance imaging (MRI) brain examinations, of which there were 3,956 in OA patients, 293 in the surgical population and 1,197 in the medication population. Cortical atrophy was observed in OA patients, including the reduced areas of the postcentral gyrus, right precentral gyrus, caudal middle frontal, left inferior parietal lobule, right temporal lobe and left middle temporal lobe, right posterior cingulate gyrus and the precuneus (Supplementary Fig. 3A). The above associations remained significant after FDR correction (Supplementary Fig. 3B). After FDR correction, the cortical atrophy observed in OA participants were not attenuated after OA treatments (Supplementary Table 19).

## Discussion

This research demonstrates that OA/OA treatment were associated with the altered risk of dementia. OA conferred a 11.6% higher risk of dementia, whereas OA treatment (surgeries and medications) lowered a 15.9–25.4% dementia risk. Notably, NSAIDs and opioids had significant protective effects on dementia. Besides, OA was related to reduced grey matter area to a large extent. Collectively, our findings indicated that OA might be a risky factor for dementia, and this risk could be reversed through OA treatments.

There are several possible mechanisms underlying the association between OA and risk of dementia. OA could release pro-inflammatory factors into the blood stream, leading to brain inflammation and subsequently contributing to higher risks of cognitive impairment and dementia [7, 34, 35]. Animal experiments observed increased neuroinflammation and aggravated AD pathology in mice by constructing an OA model [34]. An American retrospective study suggested patients with both OA and chronic pain elevated risk of dementia versus those with OA alone [5]. In addition, dementia can be attributed to depressive symptoms among OA patients [36, 37], and we found a 38.2% higher risk of dementia when OA was combined with depression. Obesity might elevate the likelihood of developing dementia among OA patients [38]. Our study revealed that the risk of dementia increased by 11.4% in patients with both OA and obesity than patients with OA alone. Overuse, improper posture, and mechanical loads are recognised to contribute to the initiation and progression of OA [32–40], while we found work intensity did not significantly influence the associations between OA and dementia risk.

NSAIDs suppresses inflammatory response by inhibiting cyclooxygenase, which thus alleviate cognitive decline [41, 42]. We found that the dementia risk fell 23.5% in OA patients taking NSAIDs than those who did not. Topical/oral opioids and NSAIDs comprise the first-line pharmacological therapies for OA [43, 44]. Various types of previous studies have pointed out that ibuprofen [45], naproxen [46] and tramadol [47] lowered the risk of dementia, and we achieved the same results. It is worth noting that all of these medications are associated with pain relief. However, there is no data in the UKB database to clearly and objectively quantify pain scales and accurately record changes in pain fluctuations among patients. Further animal experiments are therefore needed to explore the mechanisms by which more detailed and accurate drugs reduce the risk of dementia. We found no evidence of an association between glucosamine and dementia, which was in line with a previous prospective cohort study based on the UKB database [48]. Our study serves as the first to explore the association between intra-articular steroid injections and dementia risk, yielding no statistically significant association. In addition, previous studies have indicated that paracetamol is less useful for OA [49, 50]. Considering the large base of people taking it, we supplemented the analysis of the association between paracetamol-only and dementia risk while this analysis was not included in the main one. Results suggested that no statistically significance was observed for the effect of paracetamol on dementia risk in OA patients (Supplementary Table 20). In conclusion, our study is the first to restrict our participants to OA patients, and we also explored whether these OA medications could reverse the dementia risk elevated by OA. Joint replacement could improve joint movement and relieve the symptoms of OA [13], and we discovered a dramatic fall in dementia risk among OA patients who underwent OA surgeries. Furthermore, previous studies have failed to compare OA treatment group with non-OA controls. Thus, they could not figure out the independent role of OA treatment in dementia [51]. By including a healthy control group, our study design is a better way to estimate the effectiveness of OA therapy on dementia risk. Grotle et al. discovered a significant dose–response association between obesity (BMI > 30 kg/m^2^) and the risk of knee OA rather than hip OA [52]. In OA patients with a BMI ≥ 30 kg/m^2^, knee replacement surgery exerted stronger protection against dementia than hip replacement surgery. However, in this study, we extracted information on obesity from BMI with the recording time was not further clarified. We were unable to calculate the fluctuation of BMI before and after OA/OA treatment. This shortcoming awaits future UKB databases to refine the time of weight recording and calculation of the associated rate of change, or additional databases to refine this information. We also found that OA medications reduced the risk of dementia only in *APOE* ε4-positive participants, which provided new insights into dementia prevention among *APOE* ε4 carriers. Women prevail in OA patients [53] and the adults in UKB database has a large proportion of females, although we ended up with more males (54.6%) in our final analysis. We had no selection bias in the inclusion process. To account for this phenomenon, we corrected for the covariate of gender and stratified by sex and found that the male effect was still significant.

Cerebral atrophy assessed on structural MRI has been considered as an effective marker of dementia [54]. Long-term OA pain can lead to decreased quality of life [53], less exercise and poor sleep, which may cause progressive cortical thinning [55, 56]. Chronic pain was associated with brain structural alterations in temporal lobe regions [57, 58]. Temporal cortex is sensitive to AD-related pathological and cognitive changes [59], since superior temporal regions and meso-temporal regions are involved in cognitive domains, including speech perception [60], motion processing [61] and episodic memory [62]. We detected marked reduction in the areas of right and left middle temporal lobes and right superior temporal lobes in OA patients. Previous fMRI studies showed that the activities in motor and somatosensory cortices (precentral and postcentral cortices) were mainly observed in the evoked pain condition [9, 63]. A latest study found that OA was related to speeding up Aβ accumulation and more Aβ and tau deposition in precentral and postcentral cortices [11]. Consistent with the above findings, we found that the areas of postcentral and right precentral cortices in the right and left hemispheres were reduced in the OA patients. The precuneus and posterior cingulate cortex are closely associated with the AD-targeted default mode network [64, 65], and the posterior cingulate cortex connects with regions involved in emotion, executive control and memory [66]. In conclusion, the negative associations between OA and the areas of specific cortical regions indict that OA may cause structural abnormalities in these regions to enhance dementia risk [67].

There are several advantages of our study. First, it is the first comprehensive and systematic assessment of the associations of OA and its treatments with dementia and brain structures in a longitudinal cohort. Second, we utilised computerised pharmacy data to capture drug use throughout the follow-up, subsequently we can characterise medication use throughout the study to trap elaborate medication expenditure. Third, using comprehensive questionnaires and physical assessments, we took a wide range of important confounders into consideration in the analysis, including sociodemographic and lifestyle factors.

This study has several limitations. First, we lacked the detailed data about OA severity (e.g. the Kellgren–Lawrence grades and the evolution of pain levels). The dynamic changes in OA severity might influence our findings on the associations between OA and dementia risk. Second, Foot OA is also one of the more common subtypes of OA [68]. Yet in baseline recruitment of our OA population based on ICD-10 codes, we were unable to be specific to foot OA. This paucity of data relies on further refinement of the database or may be explored in other databases in the future. Third, since the imaging data were from cross-sectional studies, it is beyond our ability to conclude the causalities and temporal relationships between OA and changes in brain structures. Nonetheless, their cross-sectional associations we observed added complementary support to this longitudinal analysis.

In conclusion, our study suggested that OA was associated with increased risk of dementia and atrophic brain structures. However, OA treatments (surgeries and medications) could reverse this risk among OA patients. Therefore, OA should be regarded as a changeable risk factor in the prevention and management of dementia.

## Supplementary Material

aa-23-1966-File006_afae167

## Glossary

OA, osteoarthritis; ACD, all-caused dementia; AD, Alzheimer’s disease; VaD, vascular dementia; TPA, total physical activity; BMI, body mass index; UKB, UK Biobank; ICD-10, International Classification of Diseases, 10th edition; OPCS4, Office of Population Censuses and Surveys Classification of Interventions and Procedures; SD, standard deviation; 95%CI, 95% confidence interval; NSAIDs, nonsteroidal anti-inflammatory drugs; FDR, false discovery rate; MRI, magnetic resonance imaging.
