# Supplementary material for: Osteoarthritis, osteoarthritis treatment and risk of incident dementia: a prospective cohort study based on UK Biobank
Source: Age Ageing. 2024 Aug 7;53(8):afae167. doi: 10.1093/ageing/afae167 (PMC11303829; doi:10.1093/ageing/afae167)
Supplement: aa-23-1966-File006_afae167 [file aa-23-1966-file006_afae167.docx]

Supplemental Files

[Supplementary Methods 4](#_Toc159328776)

[Covariates 4](#_Toc159328777)

[Measurement of Incident Dementia 4](#_Toc159328778)

[Brain imaging data 4](#_Toc159328779)

[Statistical analyses 4](#_Toc159328780)

[Management of drugs and surgery 4](#_Toc159328781)

[Definition and stratification of OA factors 5](#_Toc159328782)

[Stratification and sensitivity analysis 6](#_Toc159328783)

[Supplementary Information 7](#_Toc159328784)

[Table S1. Codes used for disease diagnosis and classification in the UK Biobank. 7](#_Toc159328785)

[Table S2. UKB showcase variables for osteoarthritis treatments used in the paper. 9](#_Toc159328786)

[Table S3. UKB showcase variables for covariates used in the paper 12](#_Toc159328787)

[Table S4. UKB primary care showcase variables for OA characters used in the paper 13](#_Toc159328788)

[Table S5. List of drugs used by osteoarthritis patients and defined daily dose. 14](#_Toc159328789)

[List S1. Example calculation of total standardized daily dose 16](#_Toc159328790)

[Supplementary Tables 17](#_Toc159328791)

[Dementia analysis 17](#_Toc159328792)

[Part I OA sites 17](#_Toc159328793)

[Hierarchical analysis 19](#_Toc159328794)

[Sensitivity analysis 34](#_Toc159328795)

[Part II Other characteristics with OA 44](#_Toc159328796)

[Part III Therapy in drugs 46](#_Toc159328797)

[Part IV Therapy in surgery 56](#_Toc159328798)

[Brain structure 58](#_Toc159328799)

[Supplementary Table 20. The association of paracetamol with subsequent dementia outcome in participants. 66](#_Toc159328800)

[Supplementary figures 68](#_Toc159328801)

[Supplementary 68](#_Toc159328802)

[Supplementary Figure 1. Association of NSAID usage in OA patients with incident dementia during follow-up. 69](#_Toc159328803)

[Supplementary Figure 2. Association of opioid usage in OA patients with incident dementia during follow-up. 70](#_Toc159328804)

[Supplementary Figure 3. Association between brain structure with OA and OA treatment. 71](#_Toc159328805)

[Reference 72](#_Toc159328806)

# Supplementary Methods

## Covariates

Data on demographic, socioeconomic, and lifestyle factors were collected at baseline through questionnaires. Information on education (with or without a college or university degree), ethnicity (white or non-white including Mixed, Asian, Black), APOE4 (carrier/non-carrier status), cigarette and alcohol consumption (never, former, and current), was collected from the touchscreen questionnaire. TPA was defined by total metabolic equivalent task minutes per week for all activity including walking, moderate and vigorous activity. BMI was calculated from weight (kg) and standing height (meters) measured during the medical examination.

## Measurement of Incident Dementia

The adjudication for incident dementia was conducted by the UK Biobank Outcome Adjudication Group. All-cause dementia was ascertained using hospital inpatient records containing data on admissions and diagnoses obtained from the Hospital Episode Statistics for England, Scottish Morbidity Record data for Scotland, and the Patient Episode Database for Wales. Additional cases were detected through linkage to death register data provided by the National Health Service Digital for England and Wales and the Information and Statistics Division for Scotland. The International Classification of Diseases ICD-10 codes [1], including F00.0-9 (dementia in Alzheimer’s disease), F01.0-9 (vascular dementia), F02.0-8 (dementia in other diseases classified elsewhere), F03 (unspecific dementia), and G30.0-9 (Alzheimer’s disease), were used to identify participants with dementia if one or more of these codes were recorded as a primary or secondary diagnosis in the health records or recorded as the under lying or contributory cause of death in the death registers. A subsample of the population was also retrieved from primary care data using Read codes (Version 2 or Version 3).

## Brain imaging data

MRI scans were acquired at the baseline assessments in three imaging analysis centers that utilize similar scanners (3T Siemens Skyra with a 32-channel head coil). T1-weighted images were processed and analyzed by the UK Biobank imaging team using the FSL diffusion tensor fitting program (FMRIB) Software Library version 6.0 (http://fsl.fmrib.ox.ac.uk/fsl). The quality-controlled imaging-derived phenotypes from the Diffusion Tensor Imaging (DTI) assessments were used. Imaging data collection began in 2014 at an Imaging Assessment Centre in Cheadle and the brain data of this study were analyzed cross-sectionally. The imaging derived phenotypes (IDP) referring to atlas regions’ surface area, volume, and mean thickness, as well as subcortical volume were then processed and calculated by the UKB team. Details of the imaging protocol can be found in the online documentation (https://biobank.ctsu.ox.ac.uk/crystal/docs/brain_mri.pdf).

## Statistical analyses

### Management of drugs and surgery

We initially classified OA medication into four major categories, and initially defined the drug exposure period as from the onset of OA disease to the occurrence of the outcome event. In order to exclude potential causal bias, we excluded those who started medication only within 2 years before the occurrence of the outcome event [2], and subsequent calculations were based on the excluded population. The exposure period of surgery was treated similarly. Among the determined individuals with OA, the 33 participants who started medication only within 2 years before the end of follow-up (end point for participants without dementia and date of initial diagnosis for participants with dementia) on which the follow-up results were based, and the 26 participants who received surgery were further excluded. During our processing we found that the medication population was too broad, involving multi-system medication and mostly present in combinations. We have already analyzed the population taking combinations of medicines when processing other categories of medicines (Supplementary Table S5), and in order to avoid double counting this part of the medication taking population, we only extracted the records of single ingredient prescriptions in this processing. Previous studies showed no clinically significant difference between paracetamol and placebo in reducing pain and improving function in patients with OA [3, 4], prescription records for paracetamol were not included in this study. We did not include paracetamol as a primary analysis in the study for this treatment. Low-dose aspirin (< 300 mg per day) inhibits platelets but has no anti-inflammatory activity and is commonly used for primary and secondary prevention of cardiovascular disease in the elderly, and was not included in this study because of the confusing dosage indications for aspirin [5]. Existing studies have analyzed the opioid treatment group for safety concerns such as withdrawal symptoms, in order to minimize the risk of adverse events. In this study, addictive opioids were excluded [6, 7]. For opioid we further excluded narcotics and injectable drugs. The drugs extracted according to the British National Formulary (BNF) codes, dictionary of medicines and devices (dmd) codes, and read code codes. We defined the group of patients who the administration of the nonsteroidal anti-inflammatory drugs (NSAIDs) was nasal, eye, and mouth sprays as unmedicated. Paracetamol culls out paediatric and gastrointestinal use. In addition, in the prescription record we further replaced the prescribed dose of 0 as untaken and replaced the unclear dose as NA to facilitate the subsequent cumulative dose. To our knowledge, only a few studies have assessed the effect of different drug classes such as drug composition or chemical structure on the risk of OA and dementia [8, 9]. This article further investigates the effects of different drug classes, chemical composition, and trade names on outcomes. For comparisons of dosages, we used the “defined daily dose,” which is the average dosage of a drug taken by adults for the main indication, according to the World Health Organization [10]. All prescriptions for oral NSAIDs and opioid filled during follow-up were used to create time-dependent covariates, as described below. To investigate the possible dose-effect relationship between NSAIDs and opioids [11], we used the calculation of total standardized daily dose[12] to obtain the cumulative dose of medication taken by patients. For comparisons of dosages, we used the “defined daily dose,” which is the average dosage of a drug taken by adults for the main indication, according to the World Health Organization [10]. All prescriptions for oral NSAIDs and opioid filled during follow-up were used to create time-dependent covariates, as described below. Cumulative use of these two major drug classes during the study period was divided into four mutually exclusive categories to create time-dependent categorical variables, similar to the thresholds used in previous long-term prospective studies [13]. We conducted further drug cumulative exposure dose, chemical composition and trade name studies on the major oral types of NSAIDs and opioids. In addition disease-modifying therapeutic strategies in osteoarthritis, such as glucosamine and chondroitin, are popular and used worldwide, but they remain controversial in official recommendations and meta-analysis findings [14-16]. Therefore we further refine about the effect of glucosamine administration on dementia outcome in OA patients. Intra-articular corticosteroid alleviates OA-related pain through anti-inflammatory and immunosuppressive effects [17]. The guidelines of American College of Rheumatology strongly recommend injections for patients with OA of the knee or hip citation [18]. Therefore, we only took prescription records for intra-articular steroid injections. Arthroscopic procedures have little or no clinically important benefit for pain or function in the short or long term [19]. Arthroplasty is the only definitive procedure, and this article only explores the impact of arthroplasty on outcomes in patients with OA.

### Definition and stratification of OA factors

There is no clear diagnosis of OA pain in the UK Biobank database, and we followed the design of previous literature to investigate a series of questionnaires on pain questionnaires [20]. OA pain was defined using the options in the UK Biobank Touch Screen Questionnaire (category 154). These included headache, facial pain, neck or shoulder pain, back pain, stomach or abdominal pain, hip pain, knee pain and generalised pain. Headache, facial pain, generalized pain, and stomach or abdominal pain were not included in defining exposure, and OA pain was defined by the participant's response to two questions, "1. Do you suffer from constant or intermittent pain or discomfort that has been present for more than 3 months?" 2. "Have you had hip pain/knee pain/arm pain/foot pain for more than 3 months?" Based on the included OA population, subjects who answered "yes" to both questions were defined as having OA pain. As question 2 followed question 1, the control group consisted of those who answered "no" to question 1. This is because if participants indicated that they were not experiencing pain interference, they would not have been asked question 2: "Have you had pain in your knee for more than 3 months? and further explored the effect of duration of pain on outcome based on those with OA pain. History of joint injury (i.e., dislocation, sprain and strain and ligaments of joints, ICD-10 codes: S73 and S83) was defined as any diagnosis of these diseases before the index date (i.e., recruitment date) from the UK Biobank hospital inpatient and primary care data [21]. We performed a stratified analysis for the presence or absence of comorbid joint injury in patients with OA. Similarly, we stratified for the presence or absence of combined RA, obesity status (BMI ≥ 30). We refine the effect of different work intensities on outcomes in patients with OA. Information about physical activity were collected through the adapted questions from the well-validated long International Physical Activity Questionnaire at recruitment, which included 28 different items of physical activity-related conditions, in four major categories: activities at leisure time, job-related activities, traveling-related activities, and sedentary behaviors [21]. Information related frequency (days/week) of job was mainly extracted. (See annex table for relevant codes).

### Stratification and sensitivity analysis

In addition among the overall participants, we estimated the associations of OA and treatment with incident dementia risk in stratified analyses by age at baseline (< 60 and ≥ 60 years), gender (male and female), the onset time of dementia (dementia onset age < 65 and ≥ 65 years), APOE ɛ4 status (APOE ɛ4- and APOE ɛ4+) and osteoarthritis duration (< 5 and ≥ 5 years), TPA(< 600, 600 - 3000, ≥ 3000) and BMI (18.5, 18.5 - 30, ≥ 30). We performed sensitivity analyses to ensure the robustness of the results. To guarantee a greater accuracy of the diagnosis source of OA, those with self-declared sickness in primary care were removed. The impact of OA prevalence status and site of onset, and medication versus surgical treatment on outcome was explored by excluding patients with OA diagnosed from self-reported sources. Considering the competitive risk of all-cause mortality, we also used the competing risk analyses to determine the association of OA as exposures with subsequent dementia outcome. Inflammatory arthritis (gout [22], rheumatoid arthritis, ankylosing spondylitis and other spondyloarthritis, psoriatic arthritis [22, 23], and enteropathic arthritis [24]) may affect the risk estimate of dementia by OA, and we refined the assessment of dementia risk by OA/OAtreatment after excluding the inflammatory arthritis participants. Due to the excessive missing persons, in order to compensate for the potential selection bias, we opted for inverse probability censored weights to verify the robustness of the outcomes. Results are presented as HRs with 95% CIs.

# Supplementary Information

## Table S1. Codes used for disease diagnosis and classification in the UK Biobank.

| Diagnosis | Code Type | Codes |
| --- | --- | --- |
| ACD | ICD-9 | 290.2, 290.3, 290.4, 291.2, 294.1, 331.0, 331.1, 331.2, 331.5 |
|  | ICD-10 | A81.0, F00, F00.0, F00.1, F00.2, F00.9, F01, F01.0, F01.1, F01.2, F01.3, F01.8, F01.9, F02, F02.0, F02.1, F02.2, F02.3, F02.4, F02.8, F03, F05.1, F10.6, G30, G30.0, G30.1, G30.8, G30.9, G31.0, G31.1, G31.8, I67.3 |
|  | Read V2 | 1461, A411., A4110, E00.., E000., E001., E0010, E0011, E0012, E0013, E001z, E002., E0020, E0021, E002z, E003., E004., E0040, E0041, E0042, E0043, E004z, E012., E02y1, E041., Eu00., Eu000, Eu001, Eu002, Eu00z, Eu01., Eu010, Eu011, Eu012, Eu013, Eu01y, Eu01z, Eu02., Eu020, Eu021, Eu022, Eu023, Eu024, Eu025, Eu02y, Eu02z, Eu041, Eu106, Eu107, F110., F1100, F1101, F111., F112., F116., F118., F11x2, F11x7, F11x9, F11y2, F21y2, Fyu30, 38C13, 3AE3., 3AE4., 3AE5., 3AE6., 66h.., 6AB.., 8BM02, 8BM50, 8BM60, 8BPa., 8CMe0, 8CMG2, 8CMZ., 8CMZ0, 8CMZ1, 8CMZ2, 8CMZ3, 8CSA., 8Hla., 8IAe0, 8IAe2, 9hD.., 9hD0., 9hD1., 9Ou.., 9Ou1., 9Ou2., 9Ou3., 9Ou4., 9Ou5. |
|  | Read CTV3 | .1461, 1461, .E11., .E111, .E112, .E113, .E114, .E115, .E116, .E11Z, .F21Z, .F371, .G78., A411., A4110, E00.., E000., E001., E0010, E0011, E0012, E0013, E001z, E002., E0020, E0021, E002z, E003., E004., E0040, E0041, E0042, E0043, E004z, E012., E02y1, E041., Eu00., Eu000, Eu001, Eu002, Eu00z, Eu01., Eu010, Eu011, Eu012, Eu013, Eu01y, Eu01z, Eu02., Eu020, Eu021, Eu022, Eu023, Eu024, Eu025, Eu02y, Eu02z, Eu041, F110., F1100, F1101, F111., F112., F116., F118., F11x2, F11x7, F11y2, F21y2, Fyu30, Ub1T6, X002m, X002w, X002x, X002y, X002z, X0030, X0031, X0032, X0033, X0034, X0035, X0036, X0037, X0039, X003A, X003B, X003C, X003D, X003E, X003F, X003G, X003H, X003I, X003J, X003l, X003m, X003P, X003R, X003T, X003V, X003W, X003X, X00R2, X00Rk, Xa0lH, Xa0sC, Xa0sE, Xa1GB, Xa25J, Xa3ez, XaA1S, XabVp, XaE74, XaIKB, XaIKC, XaKyY, XaOfZ, XE17j, XE1aG, XE1Xs, XE1Xu,  XE1Z6, .3AE3, .3AE4, .3AE5, .3AE6, .66h., .6AB., .9hD1, .9Ou., .9Ou1, .9Ou2, .9 Ou3, .9Ou4, .9Ou5, 3AE3., 3AE4., 3AE5., 3AE6., 66h.., 6AB.., 8BM02, 8BM50, 8BPa., 8CMe0, 8CMG2, 8CMZ., 8CMZ0, 8CMZ1, 8CMZ2, 8CMZ3, 8CSA., 8IAe0, 8IAe2, 9hD1., 9Ou., 9Ou1., 9Ou2., 9Ou3., 9Ou4., 9Ou5., Xa0fZ, XaaBZ, XaaeA, XaaiW, Xabd2, Xabd3, XabEk, XabEl, XabtQ, XacIx, XacIy, XacIz, XacJ0, XacLx, Xacly, Xaclz, XacM2, Xaefu, XaJBQ, XaJBU, XaJBV, XaJBW, XaJBX, XaJPy, XaLFf, XaLFo, XaLFp, XaMFy, XaMG0, XaMGF, XaMGG, XaMGI, XaMGJ, XaMGK, XaMJC, XaYFR, XaYPX, XaZqJ, XaZWz |
| AD | ICD-9 | 331.0 |
|  | ICD-10 | F00, F00.0, F00.1, F00.2, F00.9, G30, G30.0, G30.1, G30.8, G30.9 |
|  | Read V2 | Eu00., Eu000, Eu001, Eu002, Eu00z, F110., F1100, F1101, Fyu30 |
|  | Read CTV3 | .F21Z, Eu00., Eu000, Eu001, Eu002, Eu00z, F110., F1100, F1101, Fyu30, X002x, X002y, X002z, X0030, X0031, X0032, X0033, X003G, XaIKB, XaIKC, XE17j |
| VD | ICD-9 | 290.4 |
|  | ICD-10 | F01, F01.0, F01.1, F01.2, F01.3, F01.8, F01.9, I67.3 |
|  | Read V2 | E004., E0040, E0041, E0042, E0043, E004z, E012., Eu01., Eu010, Eu011, Eu012, Eu013, Eu01y, Eu01z, F11x2, F21y2 |
|  | Read CTV3 | .E115, .E116, .G78., E004., E0040, E0041, E0042, E0043, E004z, Eu01., Eu010, Eu011, Eu012, Eu013, Eu01y, Eu01z, F11x2, F21y2, X003R, X003T, X003V, X003W, Xa0lH, XE1Xs |
| OA | ICD-10 | M15, M15.0, M15.00, M15.1, M15.2, M15.3, M15.4, M15.8, M15.9, M15.99, M16, M16.0, M16.1, M16.2, M16.3, M16.4, M16.5, M16.6, M16.7, M16.9, M17, M17.0, M17.1, M17.2, M17.3, M17.4, M17.5, M17.9, M18, M18.0, M18.1, M18.2, M18.3, M18.4, M18.5, M18.9, M19, M19.0, M19.00, M19.01, M19.02, M19.03, M19.04, M19.05, M19.06, M19.07, M19.08, M19.09, M19.1, M19.10, M19.11, M19.12, M19.13, M19.14, M19.15, M19.16, M19.17, M19.18, M19.19, M19.2, M19.20, M19.21, M19.22, M19.23, M19.24, M19.25, M19.26, M19.27, M19.28, M19.29, M19.8, M19.80, M19.81, M19.82, M19.83, M19.84, M19.85, M19.86, M19.87, M19.88, M19.89, M19.9, M19.90, M19.91, M19.92, M19.93, M19.94, M19.95, M19.96, M19.97, M19.98, M19.99, |
| Surgery  (joint replacement) | ICD-10 | O06, O07, O08, O10, O18, O19, O21, O22, O23, O24, O25, O26, O32, O37, O38, O39, O40, W05, W15, W37, W38, W39, W40, W40, W41, W42, W43, W44, W45, W46, W47, W48, W49, W50, W51, W52, W53, W54, W55, W56, W57, W58, W59, W60, W61, W62, W63, W64, W83, W84, W85, W86, W87, W88, W89, W93, W94, W95, W96, W97, W98, |
| RA | ICD-10 | M05, M06 |
| Psoriatic arthritis,  Enteropathic arthritis | ICD-10 | M07 |
| Gout | ICD-10 | M10 |
| Ankylosing spondylitis | ICD-10 | M45 |
| Other inflammatory spondylopathies | ICD-10 | M46 |
| Joint injury | ICD-10 | S73.0, S73.1, S83.0, S83.1, S83.2, S83.3, S83.4, S83.5, S83.6, S83.7 |

Abbreviations: ACD, All-cause dementia; AD, Alzheimer's disease; VD, Vascular dementia; OA, osteoarthritis; RA, rheumatoid arthritis; ICD, International Classification of Diseases; Read V2, Read codes version 2; Read CTV3, Read codes version 3.

## Table S2. UKB showcase variables for osteoarthritis treatments used in the paper.

| Type | Sources | Code/ Field ID |
| --- | --- | --- |
| Nonsteroidal anti-inflammatory drugs | Primary care (BNF) | "40701","100101","100302","120301","130801","4070100","10010100","10030100","10030250","12030100","13080150","15010402","01.01.02.02.00","01.03.05.00.00","04.07.01.02.00","04.07.02.00.00","0407041A0BBAAAA","0407041A0BBABAG","10.01.01.00.00","10.01.04.01.00","10.03.02.01.00","100101040AAAAAA","100101070AAAAAA","100101070BBAAAA","100101080AAAAAA","1001010AAAAAAAA","1001010AAAAABAB","1001010C0AAACAC","1001010C0AAADAD","1001010C0AAAEAE","1001010C0AAAFAF","1001010C0AAAJAJ","1001010C0AAALAL","1001010C0AAANAN","1001010C0AAARAR","1001010C0AAAUAU","1001010C0AAAWAW","1001010C0BBABAE","1001010C0BBAFAF","1001010C0BBAHAL","1001010C0BLAAAM","1001010C0BLABAX","1001010C0BMABAW","1001010C0BPAAAR","1001010E0AAAAAA","1001010E0AAACAC","1001010E0AAADAD","1001010E0BBADAD","1001010G0AAABAB","1001010I0AAACAC","1001010J0AAADAD","1001010J0AAAEAE","1001010J0AAAFAF","1001010J0AAANAN","1001010J0AAAPAP","1001010J0B8AAAR","1001010J0BCACAF","1001010J0BCAFAP","1001010J0BHACAF","1001010K0BDAFAD","1001010K0BNAAAJ","1001010L0BDAAAI","1001010L0BDABAJ","1001010N0AAAAAA","1001010N0AAADAD","1001010N0BBAAAA","1001010P0AAADAD","1001010P0AAAEAE","1001010P0AAAHAH","1001010P0AAAIAI","1001010P0AAAJAJ","1001010P0BCAIAI","1001010P0BGAAAH","1001010P0BKAAAL","1001010R0AAAAAA","1001010R0AAABAB","1001010T0BBADAD","1001010X0AAAAAA","100302010AAAAAA","1003020I0AAAAAA","1003020P0AAAAAA","1003020P0AAACAC","1003020P0BGAAAC","1003020R0AAAAAA","1003020R0BBAAAA","1003020R0BBABAA","1003020U0AAAAAA","1003020U0BBAAAA","12.03.01.00.00","1203010K0AAABAB","13.04.01.00.00","13.08.01.01.00","50.00.00.00.00" |
|  | Primary care (Read V2) | "di21.","di21.00","dibR.00","dl18.00","dl19.00","dl1b.00","h32D.","h32D.00","j212.00","j21y.00","j21z.00","j22..","j221.","j221.00","j222.00","j226.","j226.00","j229.00","j22a.00","j22B.00","j22C.","j22C.00","j22d.","j22d.00","j22e.","j22e.00","j22G.","j22G.00","j22h.00","j22i.","j22i.00","j22J.","j22n.","j22n.00","j22o.","j22o.00","j22p.","j22p.00","j22Q.","j22q.00","j22r.","j22R.00","j22t.00","j22u.00","j22v.00","j22y.","j22y.00","j22z.","j22z.00","j241.00","j247.","j247.00","j248.","j248.00","j249.","j249.00","j255.00","j262.00","j281.","j281.00","j282.","j282.00","j283.","j283.00","j287.00","j288.00","j289.00","j28A.00","j28B.00","j28d.00","j28H.00","j28u.00","j28x.00","j28Y.00","j291.","j291.00","j292.","j292.00","j293.00","j299.00","j29j.00","j29p.00","j29y.","j29y.00","j2a2.00","j2ay.00","j2az.00","j2b1.","j2b2.","j2b3.","j2b3.00","j2b5.00","j2bx.","j2bx.00","j2c1.","j2c1.00","j2c2.","j2c2.00","j2c5.00","j2c6.","j2c6.00","j2ck.","j2ck.00","j2cl.","j2cl.00","j2cq.","j2cq.00","j2cw.","j2cw.00","j2cZ.00","j2e1.00","j2e2.00","j2e3.","j2e3.00","j2e4.00","j2ew.","j2g5.00","j2i2.00","j2j1.00","j2j2.00","j2k2.","j2k2.00","j2l1.00","j2m1.00","j2m2.","j2n2.","j2n2.00","j2n3.","j2n3.00","j2n5.00","j2n7.00","j2o1.","j2oM.","j2or.00","j2oz.00","j2pJ.","j2pJ.00","j2pW.","j2pW.00","j2q1.00","j2qz.00","j2ry.00","j2rz.","j2rz.00","jA11.00","jA12.00","jA15.00","ja1A.","ja1A.00","ja1F.","ja1F.00","ja1U.","ja1U.00","ja1V.","ja1V.00","jA1w.00","jA1x.00","ja1y.","ja1y.00","ja1z.","ja1z.00","ja27.","ja27.00","ja2M.","ja2M.00","ja2N.00","ja2q.","ja2q.00","ja2U.00","ja2X.","ja2X.00","jA2y.","jA2y.00","ja2z.","ja2z.00","ja3F.","ja3F.00","ja3L.","ja3L.00","ja3M.","ja3M.00","jA51.","jA51.00","jA52.","jA52.00","jA53.","jA53.00","jA54.","jA54.00","jA55.","jA55.00","jA57.","jA58.","jA58.00","jA61.00","lb61.","lf3z.","mt1z.","mt1z.00","o4a1." |
|  | Primary care (dmd code) | "134795004","322784004","329546008","329547004","329556007","329559000","329563007","329574003","329583008","329585001","329586000","329587009","329591004","329598005","329600004","329602007","329607001","329620003","329622006","329652003","329653008","329654002","329662005","329686007","329708004","329711003","329714006","329715007","329716008","329756005","329787007","329788002","329803004","329806007","329807003","329838002","329839005","329850008","329862007","329863002","329894002","329907004","329910006","329914002","329927003","329928008","329933007","329963003","329967002","329968007","330162006","330163001","330169002","330170001","330202001","330203006","330206003","330232009","330233004","330264002","330274004","370195008","385585000","407907003","407908008","407909000","2910000000000","13000000000000","17800000000000","46100000000000","90600000000000","162000000000000","166000000000000","230000000000000","236000000000000","242000000000000","259000000000000","263000000000000","307000000000000","359000000000000","368000000000000","434000000000000","516000000000000","587000000000000","589000000000000","592000000000000","604000000000000","625000000000000","644000000000000","720000000000000","746000000000000","763000000000000","778000000000000","805000000000000","814000000000000","845000000000000","894000000000000","2840000000000000","2890000000000000","3160000000000000","3350000000000000","3360000000000000","3380000000000000","3410000000000000","3420000000000000","3440000000000000","3690000000000000","3880000000000000","3950000000000000","4570000000000000","8070000000000000","9100000000000000","10800000000000000","13100000000000000","14600000000000000","15000000000000000","16700000000000000","17300000000000000","18200000000000000","18600000000000000","19200000000000000","22500000000000000","25500000000000000","29000000000000000","42100000000000000","42200000000000000","78500000000000000","110000000000000000","114000000000000000","154000000000000000","176000000000000000","190000000000000000","192000000000000000","196000000000000000","213000000000000000","299000000000000000" |
| Opioid | Primary care (BNF) | "30102","40701","40702","40901","110801","1040200","4070100","4070200","01.04.02.00.00","02.08.02.00.00","03.01.02.00.00","03.04.01.02.00","03.09.01.00.00","0309010C0AAABAB","0309010C0AAADAD","04.06.01.00.00","04.07.01.01.00","04.07.01.02.00","04.07.02.00.00","04.09.01.02.00","0407010F0AAAAAA","0407010F0AAABAB","0407010F0AAACAC","0407010F0AAADAD","0407010F0AAAFAF","0407010F0AAAHAH","0407010F0BEAAAD","0407010M0AAABAB","0407010N0AAAAAA","040702040AAAAAA","040702040AAACAC","040702040AAADAD","040702040AAAFAF","040702040AAAGAG","040702040AAAHAH","040702040AAAIAI","040702040AAAJAJ","040702040BBACAC","040702040BDACAH","0407020A0AAAEAE","0407020A0AAAFAF","0407020A0AAAGAG","0407020C0AAADAD","0407020C0AAAEAE","0407020G0AAACAC","0407020G0AAADAD","0407020G0AAAEAE","0407020G0AAAIAI","0407020G0BBAAAD","0407020G0BBABAE","0407020H0BBAAAB","0407020L0AAABAB","0407020Q0AAAUAU","0407020Q0AACDCD","0407020Q0AACECE","0407020Q0BBAAAK","0407020Q0BBABAL","0407020Q0BBAGCP","0407020Q0BBAKCG","0407020Q0BCAGCN","0407020Q0BHACCD","0407020T0AAABAB","0407020V0AAACAC","06.01.05.00.00","07.04.05.00.00","11.08.01.00.00","15.01.07.00.00","50.00.00.00.00" |
|  | Primary care (Read V2) | "a811.","a811.00","a812.","a812.00","a813.","a813.00","c331.00","cg11.00","cg16.00","dia2.","dia2.00","dia4.00","dia6.","dia6.00","diam.00","dian.","dian.00","diap.","diap.00","diaq.","diaq.00","diaU.","diaU.00","dibB.","dibB.00","dibD.","dibD.00","dibE.","dibE.00","dibF.","dibQ.","dibQ.00","dibt.","dibt.00","dicO.00","dicv.00","dicz.","dicz.00","dj1f.","dj1f.00","dj1g.","dj1h.","dj1h.00","dj1l.","dj1l.00","dj1N.","dj1Q.00","dj1r.","dj1y.","dj1y.00","dj1z.","dj1z.00","dj41.00","dj42.","dj42.00","dj43.00","dj44.","dj44.00","dj81.","dj81.00","dj87.","dj87.00","dj88.","dj88.00","dj89.00","dj8a.","dj8b.","dj8b.00","dj8c.","dj8e.00","djAb.","djAc.","djAe.","djAe.00","djAh.00","djb3.00","djBU.","djBZ.","dji2.","dji2.00","dji4.00","dji5.","dji5.00","dji6.00","dji7.","dji7.00","dji8.00","djiC.","djiF.","djiF.00","djiG.","djiG.00","djiH.","djiH.00","djiI.00","djiv.00","djiw.00","djj1.00","djjB.00","djjC.00","djjD.00","djk1.","djk1.00","djk2.","djk4.","djk4.00","djk5.","djk5.00","djkG.","djkI.","djkI.00","djkJ.","djkJ.00","djkL.","djkL.00","djkM.00","djkN.","djkN.00","djkO.00","djkp.","djkp.00","djkq.","djkq.00","djkr.","djkr.00","djks.","djks.00","djkt.","djku.","djku.00","djkw.","djkw.00","djkx.","djkY.","djkY.00","djkZ.00","djmI.00","djmJ.","djmJ.00","djy6.00","djz1.","djz1.00","djz3.","djz3.00","djz4.","djz4.00","djz5.","djz6.","djz6.00","djz7.","djzD.","djzD.00","djzE.00","djzj.00","djzk.00","djzl.00","j28V.00","o424.","o424.00","o425.","o42I.","o42I.00","o42J.00","o42K.","o42R.","o42R.00","o42S.","o42T.","o42T.00","o42U.00","o4dB.00","o4dD.00","o4dF.00","o4e3." |
|  | Primary care (dmd code) | "322366004","322341003","322311000","322502004","322503009","322307006","322623000","3805610000000000","18505000000000000","322344006","322546007","7386810000000000","322504003","3164310000000000","322711002","3075210000000000","299275000000000000","322343000","23657800000000000","322637009","922411000000000","322636000","9089910000000000","322365000","322539003","322721005","5212810000000000","15065900000000000","18643300000000000","2895710000000000","18644600000000000","322638004","3077710000000000","19200200000000000","19486700000000000","360311000000000","19487500000000000","110785000000000000","3652110000000000","320962001","322710001","154925000000000000","212265000000000000","322689004","19624400000000000","14976800000000000","3651610000000000","86345000000000000","3037510000000000","3778010000000000","3037110000000000","12037400000000000","322713004","322626008","322694004","20937700000000000","20938100000000000","20937500000000000","19230700000000000","4389410000000000","203811000000000","322639007","322550000","60375000000000000","15499400000000000","9089710000000000","115895000000000000","136735000000000000","154975000000000000","11085300000000000","322553003","322505002","20937900000000000","4074910000000000","352411000000000","322440003","2898210000000000","11084900000000000","136665000000000000","329683004","212255000000000000","9752310000000000","23658000000000000","2883210000000000","322708003","9478910000000000","7381610000000000","22785300000000000","19487100000000000","14985800000000000","333919005","136725000000000000","322379008","333936002","272811000000000","103785000000000000","8095110000000000","322665006","4001410000000000","3838510000000000","3870410000000000","2891710000000000","29838600000000000","29838400000000000","322628009","226655000000000000","322720006","20545300000000000","232711000000000","322692000","8427710000000000","136685000000000000","306311000000000","407919006","2896610000000000" |
| Glucosamine | Primary care (BNF) | "10.01.05.00.00","21.01.00.00.00" |
|  | Primary care (Read V2) | "iz1a.00","iz1J.00","iz1M.00","iz1O.00","iz1R.00","iz1U.","iz1X.00","iz1Y.","iz1Y.00","iz1Z.","iz1Z.00" |
|  | Primary care (dmd code) | "4283411000001100","4283511000001100","4283611000001100","7889511000001100","7904211000001100","9742711000001100","11146411000001100","11359411000001100","11393511000001100","12185711000001100","12298911000001100","17235911000001100","19519211000001100","24112711000001100","176495001000027000","235815001000027000","241485001000027000","244305001000027000" |
| Intraarticular injection of steroid | Primary care (BNF) | "6030200","10010202","06.03.01.02.00","06.03.02.00.00","0603020M0AAAAAA","0603020Z0AAAAAA","0603020Z0AAABAB","10.01.02.02.00","1001022K0AAAAAA","1001022K0AAABAB","1001022K0AAAEAE","1001022K0BBAAAA","1001022K0BBAFAF","1001022K0BBAGAG" |
|  | Primary care (Read V2) | "fe46.00","fe4d.00","fe5b.","fe5b.00","fe5c.","fe5c.00","fe5e.","fe5e.00","fe83.00","fe88.00","fe8x.","fe8x.00","fe8y.00","j421.00","j423.","j423.00","j431.00","j434.00","j435.","j435.00","j436.","j436.00","j43w.","j43w.00","j43y.","j43z.00","j441.00","j462.00","j463.00","j46x.00","j46z.00","j473.00","j47z.00" |
|  | Primary care (dmd code) | "623611000000000","306011000000000","470611000000000","65635000000000000","65615000000000000","777311000000000","125255000000000000","769111000000000","3017510000000000","3231810000000000","330002005","325398000","865511000000000","325423003","329978005","299275000000000000","325385009","116915000000000000","150805000000000000","325467001","426832001","68285000000000000","325422008","355311000000000","15845000000000000","16845000000000000","243245000000000000","16157400000000000","73735000000000000","325397005","112465000000000000","56825000000000000","355001000000000","199635000000000000","144905000000000000","70765000000000000" |
| Paracetamol | Primary care (BNF) | "04.07.01.02.00","04.07.01.02.00","4070100",,"4070100","0407010H0AAAMAM","40701","0407010H0AAAQAQ","50.00.00.00.00", |
|  | Primary care (Read V2) | "di21.","di21.00","di2q.","di2q.00","di22.00","di22.","di2u.","di24.","dibD.00","dian.00","di2u.00","didu.00","didv." |
|  | Primary care (dmd code) | "322236009","322280009","322237000","172000000000000","322788001","155000000000000000","137000000000000000","299000000000000000","836000000000000","322284000","21600000000000000","16700000000000000","3320000000000000","5870000000000000" |

Abbreviates: BNF, British National Formulary; Read V2, Read Codes Version 2; ICD, International Classification of Diseases.

^a^ Digitals outside the parentheses were field IDs in the UKB, while digitals within the parentheses were subcategories of the field ID listed before the parentheses.

## Table S3. UKB showcase variables for covariates used in the paper

| Variables | Field ID | Definition |
| --- | --- | --- |
| Age | 21022 | Age at baseline (instance 0, 2006-10) |
| Sex | 31 | Male and female |
| Ethnic | 21000 | Ethnic background, categorized to white, mixed, Asian and black. |
| Education | 6138 | Qualification levels were categorized as higher education level (college / university degree or with other professional qualifications) and lower education level (A levels/AS levels, O levels/GCSEs, CSEs, NVQ or HND or HNC, equivalent of above, or none of the listed above). |
| Smoking status | 20116 | The part/current smoking status of participants, including never smoke, past smokers, and current smokers |
| Alcohol consumption | 1558 | Questions of alcohol intake frequency, answers included daily or almost daily, three or four times a week, once or twice a week, one to three times a month, special occasions only, and never |
| BMI | 21001 | BMI was constructed from height and weight measured during the initial Assessment Centre visit |
| APOEε4 | SNP | Carrier/non-carrier status as defined by genetic information |
| TPA | 22040 | Total Metabolic Equivalent Task minutes per week for all activity including walking, moderate and vigorous activity. |

Abbreviations: BMI, body mass index; TPA, total physical activity; APOE, Apolipoprotein E

## Table S4. UKB primary care showcase variables for OA characters used in the paper

| Variables | Field ID | Definition |
| --- | --- | --- |
| Work involving walk/standing | 816 | Job involves heavy manual or physical work |
| Work involving heavy manual/ physical work | 806 | Work involving walk/standing: |
| Pain experience status | 120019 | Troubled by pain or discomfort present for more than 3 months |
| OA status in pain experience questionnaires | 120000 | Ever had osteoarthritis affecting one or more joints (e.g. hip, knee, shoulder) |
| Duration of pain experience | 120020 | Length of time of pain or discomfort:  How long have you been suffering with this pain or discomfort? |
| Location of pain | 120028-120034 |  |

Abbreviations: OA, osteoarthritis

## Table S5. List of drugs used by osteoarthritis patients and defined daily dose.

| **Nonsteroidal anti-inflammatory drugs**  Salicyclic acid:   - Salicylate - Benorilate (3000mg) ^a^ - Diflunisal (750mg) ^a^ - Benorilate (3000mg) ^a^   Acetic acid:   - Indomethacin (100mg)^a^ - Diclofenac (100mg) ^a^ - Etodolac (400mg) ^a^ - Aceclofenac (200mg) ^a^ - Sulindac 400mg - Acemetacin 120mg ^a^ - Tolmetin (700mg)   Propionic acid:   - Ibuprofen (1200mg) ^a^ - Naproxen (500mg) ^a^ - Ketoprofen (150mg) ^a^ - Fenoprofen (1200mg) ^a^ - Fenbufen (600mg) ^a^ - Flurbiprofen (200mg) ^a^ - Flubiprofen - Dexketoprofen (75mg) ^a^ - Dexibuprofen (800mg) ^a^ - Tiaprofenic (600mg) ^a^ - Suprofen (400mg) ^a^   Coxib:   - Celecoxib (200mg) ^a^ - Etoricoxib (60mg) ^a^ - Lumiracoxib (100mg) ^a^ - Valdecoxib 10mg ^a^ - Rofecoxib 25mg ^a^ | Oxicams:   - Piroxicam (20mg) ^a^ - Tenoxicam (20mg ) ^a^ - Meloxicam (15mg) ^a^ - Lornoxicam (12mg)   Fenamates:   - Mefenacmic (1000mg) ^a^ - Flufenamic (500mg) ^a^   Butylpyrazolidines:   - Phenylbutazone (300mg) ^a^ - Oxyphenbutazone (300mg) ^a^   Other:   - Nabumetone (1000mg) ^a^ - Azapropazone 750mg ^a^ - Nimesulide (200mg) ^a^   **Opioid**  natural opium alkaloids:   - Morphine (100mg) ^a^ - Oxycodone (75mg) ^a^ - Codeine (100mg) ^a^ - 30codeine/500paracet (3UD) ^a^ - 20codeine/500paracet (4UD) ^a^ - 7.46-15codeine/500paracet (6UD) ^a^ - Dihydrocodeine (150mg) ^a^ - Papaveretum - Pentazocine (200mg) ^a^ - Opium   Diphenylpropylamine:   - Dextromoramide (20mg) ^a^ - Dextropropoxyphene (chloride 200mg, napsylate 300mg) ^a^ - Methadone (25mg) ^a^ | Oripavine derivatives:   - Buprenorphine (1200mg) ^a^   Phenylpiperidine derivatives:   - Pethidine (400mg) ^a^ - Fentanyl (600mg) ^a^   Benzomorphan derivatives:   - Pentazocine (200mg) ^a^   Morphinan derivatives:   - Butorphanol (12mg) ^a^ - Nalbuphine (80mg) ^a^   Other:   - Tramadol (300mg) ^a^ - 37.5tramadol/325paracet (4UD) ^a^ - Meptazinol (1200mg) ^a^ - Tapentadol (400mg) ^a^   **Glucosamine** (1500mg) ^a^  **Corticosteroid**  Glucocorticoids:   - Hydrocort (30mg) ^b^ - Methylpred (20mg) ^b^ - Prednisolone (10mg) ^b^ - Triamcinol (7.5mg) ^b^ - **Paracetamol** (3000mg) |
| --- | --- | --- |

^a^ Defined daily dose for oral medicine; ^b^ intraarticular injection of steroid; Abbreviation: UD, unit dose

## List S1. Example calculation of total standardized daily dose

Participant has the following medication fills extending from diagnoses of OA through the outcome:

Eg: 2010: Filled a new prescription for 90 tablets of meclizine 25 mg which was refilled once;

2011: Filled a new prescription for 60 tablets of oxybutynin 10 mg which was refilled 5 times.

**Diclofenac SSD:** $\sum\boldsymbol{Diclofenac fills=}\frac{\boldsymbol{2 fills (90}\boldsymbol{tablets)(25}\boldsymbol{mg)}}{\boldsymbol{100}\boldsymbol{m}\boldsymbol{g}^{\boldsymbol{a}}}\boldsymbol{=45 SDD}$

**Ibuprofen SDD:**$\sum\mathbf{Ibuprofen}\boldsymbol{fills=}\frac{\boldsymbol{6 fills (60 tablets)(600}\boldsymbol{mg)}}{\boldsymbol{1200}\boldsymbol{m}\boldsymbol{g}^{\boldsymbol{b}}}\boldsymbol{=180 SDD}$

TSDD = 45 SDD+180 SDD =225

Abbreviations: SDD, standardized daily dose; TSDD, total standardized daily dose

a: the basic definition of the defined daily dose for diclofenac

b: the basic definition of the defined daily dose for ibuprofen

# Supplementary Tables

# Dementia analysis

### Part I OA sites

#### Supplementary Table 1. Association between different parts of osteoarthritis and dementia in UK Biobank, after adjustments.

|  | **Number of incident dementia/ Number of participants** | **Model 1**  **(Gender+Age)** | | **Model 2**  **(+APOE ε4+Ethnicity+Education+TPA**  **+BMI+Smoking+Alcohol)** | |
| --- | --- | --- | --- | --- | --- |
|  |  | **HR (95%CI)** | **P value** | **HR (95%CI)** | **P value** |
| **ACD** | | | | | |
| **Non-OA** | 4330/403379 |  | | | |
| **OA** | 1297/63081 | **1.113(1.046-1.185)** | **0.001** | **1.116(1.039-1.199)** | **0.003** |
| **Site of OA** |  | | | | |
| Hand OA | 93/4087 | 1.110(0.903-1.364) | 0.321 | 1.013(0.795-1.292) | 0.916 |
| Hip OA | 186/6860 | **1.238(1.069-1.434)** | **0.004** | **1.335(1.135-1.570)** | **<0.001** |
| Knee OA | 363/14155 | **1.324(1.190-1.475)** | **<0.001** | **1.325(1.171-1.499)** | **<0.001** |
| Hand OA only | 27/1527 | 0.964(0.660-1.408) | 0.851 | 0.836(0.526-1.329) | 0.448 |
| Hip OA only | 62/2845 | 1.025(0.797-1.317) | 0.849 | 1.191(0.910-1.559) | 0.203 |
| Knee OA only | 163/7346 | **1.279(1.093-1.495)** | **0.002** | **1.357(1.138-1.617)** | **0.001** |
| Hand and hip OA | 1/95 | 0.447(0.063-3.176) | 0.421 | - | - |
| Hand and knee OA | 8/201 | 1.649(0.824-3.299) | 0.158 | 1.915(0.912-4.022) | 0.086 |
| Knee and hip OA | 13/253 | **2.201(1.277-3.795)** | **0.005** | **2.197(1.180-4.091)** | **0.013** |
| Knee, hip and hand OA | 3/23 | **6.040(1.947-18.737)** | **0.002** | 3.727(0.931-14.923) | 0.063 |
| **AD** | | | | | |
| **Non-OA** | 1862/400911 |  | | | |
| **OA** | 576/62360 | **1.102(1.003-1.211)** | **0.044** | **1.127(1.013-1.254)** | **0.028** |
| **Site of OA** |  | | | | |
| Hand OA | 47/4041 | 1.215(0.909-1.624) | 0.189 | 1.080(0.759-1.535) | 0.670 |
| Hip OA | 77/6751 | 1.140(0.907-1.432) | 0.263 | 1.266(0.983-1.632) | 0.068 |
| Knee OA | 169/13961 | **1.403(1.198-1.642)** | **<0.001** | **1.432(1.192-1.719)** | **<0.001** |
| Hand OA only | 11/1511 | 0.857(0.474-1.551) | 0.611 | 0.826(0.412-1.655) | 0.590 |
| Hip OA only | 23/2806 | 0.851(0.564-1.283) | 0.440 | 1.072(0.696-1.650) | 0.753 |
| Knee OA only | 75/7258 | **1.358(1.078-1.710)** | **0.010** | **1.520(1.177-1.963)** | **0.001** |
| Hand and hip OA | 1/95 | 0.966(0.136-6.860) | 0.972 | - | - |
| Hand and knee OA | 5/198 | 2.262(0.940-5.441) | 0.069 | 2.576(0.964-6.882) | 0.059 |
| Knee and hip OA | 6/246 | **2.421(1.086-5.398)** | **0.031** | 2.336(0.875-6.241) | 0.091 |
| **Knee, hip and hand OA** | 2/22 | **8.601(2.148-34.431)** | **0.002** | 3.954(0.556-28.140) | 0.170 |
| **VD** | | | | | |
| **Non-OA** | 998/400047 |  | | | |
| **OA** | 314/62098 | 1.177(1.035-1.337) | 0.013 | 1.115(0.962-1.291) | 0.148 |
| **Site of OA** |  | | | | |
| Hand OA | 24/4018 | 1.292(0.861-1.939) | 0.217 | 1.236(0.782-1.954) | 0.364 |
| Hip OA | 46/6720 | 1.311(0.975-1.763) | 0.073 | 1.303(0.931-1.824) | 0.123 |
| Knee OA | 81/13873 | **1.255(1.0002-1.575)** | **0.0498** | 1.224(0.949-1.578) | 0.120 |
| Hand OA only | 8/1508 | 1.279(0.638-2.567) | 0.488 | 0.817(0.305-2.184) | 0.687 |
| Hip OA only | 16/2799 | 1.110(0.677-1.819) | 0.680 | 1.199(0.692-2.076) | 0.517 |
| Knee OA only | 38/7221 | 1.255(0.907-1.735) | 0.170 | 1.263(0.880-1.813) | 0.205 |
| Hand and hip OA | 0/94 | - | - | - | - |
| Hand and knee OA | 3/196 | 2.655(0.855-8.248) | 0.091 | **3.253(1.045-10.130)** | **0.042** |
| Knee and hip OA | 2/242 | 1.455(0.363-5.827) | 0.596 | 0.859(0.121-6.113) | 0.789 |
| Knee, hip and hand OA | 1/21 | **9.398(1.322-66.825)** | **0.025** | **7.923(1.112-56.470)** | **0.039** |

The results are derived from Cox proportional hazard regression models in two models. Bold indicates statistical significance (P value<0.05).

Model 2 was additionally controlled for APOE ε4, ethnicity, education, TPA, BMI, smoking and alcohol than model 1.

Abbreviation: OA, osteoarthritis; ACD, all-cause dementia; AD, Alzheimer’s disease; VD, vascular dementia; APOE, Apolipoprotein E; BMI, body mass index; TPA, total physical activity; HR, Hazard ratio; CI, Confidence interval.

## Hierarchical analysis

#### Supplementary Table 2. Interaction terms for some factors

| **P for interaction** | **OA** | | **Drug** | | **Surgery** | |
| --- | --- | --- | --- | --- | --- | --- |
|  | **Model 1** | **Model 2** | **Model 1** | **Model 2** | **Model 1** | **Model 2** |
| **Age** | 0.194 | 0.140 | 0.187 | 0.338 | 0.320 | 0.556 |
| **Gender** | 0.639 | 0.701 | 0.078 | **0.044** | 0.482 | 0.462 |
| **APOE ε4** | 0.993 | 0.639 | 0.064 | 0.051 | 0.366 | 0.677 |
| **OA duration** | **<0.001** | **0.001** |  |  |  |  |
| **Dementia onset** | - | 0.973 | **<0.001** | **<0.001** | **<0.001** | **0.001** |
| **BMI** | 0.057 | 0.054 | 0.947 | 0.990 | 0.364 | 0.477 |
| **TPA** | 0.036 | 0.150 | 0.340 | 0.321 | 0.761 | 0.893 |

Abbreviations: BMI, body mass index; TPA, total physical activity; OA, osteoarthritis

*Model 1: Adjusted for age, sex

*Model 2: Adjusted for all covariates

#### Supplementary Table 3. Association of different parts of osteoarthritis with therapy and dementia in subgroups of age.

| Exposure elements | **Number of incident dementia/ Number of participants** | **Model 1**  **(Gender+Age)** | | **Model 2**  **(+APOE ε4+Ethnicity+Education+TPA**  **+BMI+Smoking+Alcohol)** | |
| --- | --- | --- | --- | --- | --- |
|  |  | **HR (95%CI)** | **P value** | **HR (95%CI)** | **P value** |
| **Age <60 years old** | | | | | |
| **Non-OA** | 594/237209 |  | | | |
| **OA** | 120/21839 | **1.400(1.148-1.707)** | **0.001** | **1.404(1.119-1.761)** | **0.003** |
| **Site of OA** |  | | | | |
| Hand OA | 7/1127 | 1.415(0.671-2.986) | 0.362 | 0.998(0.372-2.678) | 0.996 |
| Hip OA | 11/4775 | 1.440(0.792-2.616) | 0.232 | 1.168(0.553-2.470) | 0.684 |
| Knee OA | 28/4775 | **1.499(1.025-2.191)** | **0.037** | **1.827(1.223-2.728)** | **0.003** |
| **Drug** |  |  |  |  |  |
| **Nonuse ^c^** | 63/11859 |  |  |  |  |
| **Drug** | 30/5608 | 0.876(0.567-1.354) | 0.551 | 0.883(0.571-1.365) | 0.576 |
| **Drug_kind** |  |  |  |  |  |
| NSAID | 19/3643 | 0.834(0.499-1.393) | 0.488 | 0.840(0.502-1.404) | 0.506 |
| Opioid | 17/3841 | 0.697(0.408-1.191) | 0.186 | 0.698(0.408-1.193) | 0.188 |
| Glucosamine | 3/299 | 1.744(0.546-5.570) | 0.348 | 1.708(0.534-5.464) | 0.367 |
| Steroids injected | 13/1420 | 1.491(0.820-2.710) | 0.190 | 1.473(0.809-2.682) | 0.206 |
| **Surgery** |  |  |  |  |  |
| **Non-surgery** | 94/17447 |  |  |  |  |
| **OA surgery** | 23/4298 | 0.696(0.441-1.100) | 0.121 | 0.785(0.480-1.282) | 0.332 |
| Hip replacement | 9/1851 | 0.673(0.339-1.334) | 0.256 | 0.743(0.358-1.543) | 0.426 |
| Knee replacement | 10/2027 | 0.624(0.325-1.201) | 0.158 | 0.687(0.340-1.389) | 0.296 |
| **Age ≥60 years old** | | | | | |
| **Non-OA** | 3736/166170 |  | | | |
| **OA** | 1177/41242 | **1.087(1.018-1.161)** | **0.013** | **1.090(1.011-1.175)** | **0.024** |
| **Site of OA** |  | | | | |
| Hand OA | 86/2960 | 1.086(0.876-1.345) | 0.451 | 1.014(0.789-1.302) | 0.915 |
| Hip OA | 175/5058 | **1.218(1.046-1.418)** | **0.011** | **1.340(1.135-1.583)** | **0.001** |
| Knee OA | 335/9380 | **1.308(1.169-1.463)** | **<0.001** | **1.287(1.131-1.466)** | **<0.001** |
| **Drug** |  |  |  |  |  |
| **Nonuse ^c^** | 629/22174 |  |  |  |  |
| **Drug** | 273/12215 | **0.732(0.635-0.843)** | **<0.001** | **0.733(0.636-0.845)** | **<0.001** |
| **Drug_kind** |  |  |  |  |  |
| NSAID | 170/7802 | **0.705(0.595-0.835)** | **<0.001** | **0.706(0.597-0.838)** | **<0.001** |
| Opioid | 203/8590 | **0.750(0.640-0.879)** | **<0.001** | **0.751(0.641-0.880)** | **<0.001** |
| Glucosamine | 30/992 | 0.967(0.670-1.394) | 0.856 | 0.969(0.672-1.398) | 0.866 |
| Steroids injected | 91/3372 | 0.875(0.702-1.090) | 0.234 | 0.862(0.692-1.075) | 0.188 |
| **Surgery** |  |  |  |  |  |
| **Non-surgery** | 742/27526 |  |  |  |  |
| **OA surgery** | 396/13410 | **0.805(0.712-0.910)** | **0.001** | **0.845(0.736-0.971)** | **0.017** |
| Hip replacement | 163/5766 | **0.791(0.667-0.937)** | **0.007** | 0.854(0.708-1.031) | 0.100 |
| Knee replacement | 209/6645 | **0.837(0.717-0.976)** | **0.024** | **0.834(0.698-0.995)** | **0.044** |

The results are derived from Cox proportional hazard regression models in two models. Bold indicates statistical significance (P value<0.05).

Model 2 was additionally controlled for APOE ε4, ethnicity, education, TPA, BMI, smoking and alcohol than model 1.

Abbreviation: OA, osteoarthritis; ACD, all-cause dementia; AD, Alzheimer’s disease; VD, vascular dementia; APOE, Apolipoprotein E; BMI, body mass index; TPA, total physical activity; HR, Hazard ratio; CI, Confidence interval; NSAID, nonsteroidal anti-inflammatory drugs.

#### Supplementary Table 4. Association of different parts of osteoarthritis with therapy and dementia in subgroups of onset age.

|  | **Number of incident dementia/ Number of participants** | **Model 1**  **(Gender+Age)** | | **Model 2**  **(+APOE ε4+Ethnicity+Education+TPA**  **+BMI+Smoking+Alcohol)** | |
| --- | --- | --- | --- | --- | --- |
|  |  | **HR (95%CI)** | **P value** | **HR (95%CI)** | **P value** |
| **Early onset <65 years old** | | | | | |
| **Non-OA** | 483/205623 |  | | | |
| **OA** | 103/15638 | **1.795(1.450-2.223)** | **<0.001** | **1.785(1.393-2.286)** | **<0.001** |
| **Site of OA** |  | | | | |
| Hand OA | 7/679 | **2.354(1.115-4.972)** | **0.025** | 1.751(0.651-4.706) | 0.267 |
| Hip OA | 8/1334 | 1.542(0.766-3.102) | 0.225 | 1.261(0.521-3.053) | 0.607 |
| Knee OA | 25/3416 | **1.963(1.313-2.935)** | **0.001** | **2.404(1.568-3.686)** | **<0.001** |
| **Drug** |  |  |  |  |  |
| **Nonuse ^c^** | 58/9133 |  |  |  |  |
| **Drug** | 20/3282 | 0.746(0.446-1.245) | 0.263 | 0.755(0.451-1.262) | 0.283 |
| **Drug_kind** |  |  |  |  |  |
| NSAID | 13/2079 | 0.773(0.421-1.419) | 0.407 | 0.795(0.433-1.460) | 0.460 |
| Opioid | 11/2166 | 0.626(0.327-1.199) | 0.158 | 0.616(0.321-1.181) | 0.144 |
| Glucosamine | 2/179 | 1.300(0.316-5.349) | 0.716 | 1.157(0.280-4.788) | 0.841 |
| Steroids injected | 10/811 | 1.539(0.782-3.031) | 0.212 | 1.449(0.734-2.862) | 0.285 |
| **Surgery** |  |  |  |  |  |
| **Non-surgery** | 86/13350 |  |  |  |  |
| **OA surgery** | 14/2234 | 0.659(0.374-1.161) | 0.149 | 0.683(0.367-1.271) | 0.229 |
| Hip replacement | 6/1027 | 0.642(0.281-1.470) | 0.295 | 0.668(0.268-1.664) | 0.386 |
| Knee replacement | 6/992 | 0.599(0.261-1.373) | 0.226 | 0.595(0.237-1.494) | 0.269 |
| **Late onset ≥65 years old** | | | | | |
| **Non-OA** | 3847/197756 |  | | | |
| **OA** | 1194/47443 | **1.083(1.015-1.156)** | **0.017** | **1.086(1.008-1.170)** | **0.030** |
| **Site of OA** |  | | | | |
| Hand OA | 86/3408 | 1.073(0.866-1.329) | 0.519 | 0.994(0.774-1.277) | 0.963 |
| Hip OA | 178/5526 | **1.217(1.047-1.415)** | **0.011** | **1.332(1.129-1.572)** | **0.001** |
| Knee OA | 338/10739 | **1.298(1.161-1.451)** | **<0.001** | **1.277(1.122-1.452)** | **<0.001** |
| **Drug** |  |  |  |  |  |
| **Nonuse ^c^** | 634/24900 |  |  |  |  |
| **Drug** | 283/14541 | **0.754(0.656-0.868)** | **<0.001** | **0.756(0.657-0.870)** | **<0.001** |
| **Drug_kind** |  |  |  |  |  |
| NSAID | 176/9366 | **0.724(0.612-0.855)** | **<0.001** | **0.726(0.614-0.858)** | **<0.001** |
| Opioid | 209/10265 | **0.766(0.655-0.895)** | **0.001** | **0.767(0.656-0.897)** | **0.001** |
| Glucosamine | 31/1112 | 1.007(0.702-1.444) | 0.970 | 1.014(0.707-1.455) | 0.938 |
| Steroids injected | 94/3981 | 0.902(0.726-1.121) | 0.352 | 0.890(0.716-1.106) | 0.293 |
| **Surgery** |  |  |  |  |  |
| **Non-surgery** | 750/31623 |  |  |  |  |
| **OA surgery** | 405/15474 | 0.817(0.723-0.922) | 0.001 | 0.861(0.751-0.988) | 0.032 |
| Hip replacement | 166/6590 | 0.797(0.673-0.943) | 0.008 | 0.864(0.717-1.041) | 0.123 |
| Knee replacement | 213/7680 | 0.846(0.726-0.986) | 0.032 | 0.847(0.711-1.010) | 0.064 |

The results are derived from Cox proportional hazard regression models in two models. Bold indicates statistical significance (P value<0.05).

Model 2 was additionally controlled for APOE ε4, ethnicity, education, TPA, BMI, smoking and alcohol than model 1.

Abbreviation: OA, osteoarthritis; ACD, all-cause dementia; AD, Alzheimer’s disease; VD, vascular dementia; APOE, Apolipoprotein E; BMI, body mass index; TPA, total physical activity; HR, Hazard ratio; CI, Confidence interval; NSAID, nonsteroidal anti-inflammatory drugs.

#### Supplementary Table 5. Association of different parts of osteoarthritis with therapy and dementia in subgroups of gender.

|  | **Number of incident dementia/ Number of participants** | **Model 1**  **(Gender+Age)** | | **Model 2**  **(+APOE ε4+Ethnicity+Education+TPA**  **+BMI+Smoking+Alcohol)** | |
| --- | --- | --- | --- | --- | --- |
|  |  | **HR (95%CI)** | **P value** | **HR (95%CI)** | **P value** |
| **Female** | | | | | |
| **Non-OA** | 2416/186832 |  | | | |
| **OA** | 579/24719 | **1.099(1.004-1.204)** | **0.042** | 1.103(0.996-1.222) | 0.059 |
| **Site of OA** |  | | | | |
| Hand OA | 33/1182 | 1.148(0.814-1.619) | 0.431 | 1.246(0.863-1.800) | 0.240 |
| Hip OA | 94/3049 | **1.276(1.038-1.569)** | **0.021** | **1.369(1.091-1.716)** | **0.007** |
| Knee OA | 207/7096 | **1.369(1.188-1.578)** | **<0.001** | **1.378(1.174-1.617)** | **<0.001** |
| **Drug** |  |  |  |  |  |
| **Nonuse ^c^** | 313/13654 |  |  |  |  |
| **Drug** | 145/7157 | **0.798(0.656-0.972)** | **0.025** | **0.811(0.667-0.988)** | **0.038** |
| **Drug_kind** |  |  |  |  |  |
| NSAID | 86/4574 | **0.748(0.589-0.950)** | **0.017** | **0.761(0.599-0.967)** | **0.025** |
| Opioid | 109/4982 | 0.837(0.673-1.040) | 0.109 | 0.844(0.679-1.050) | 0.129 |
| Glucosamine | 12/461 | 0.870(0.489-1.548) | 0.635 | 0.874(0.491-1.557) | 0.648 |
| Steroids injected | 58/1898 | 1.166(0.881-1.544) | 0.282 | 1.180(0.891-1.564) | 0.247 |
| **Surgery** |  |  |  |  |  |
| **Non-surgery** | 358/17195 |  |  |  |  |
| **OA surgery** | 203/7355 | 0.828(0.696-0.985) | 0.033 | 0.872(0.720-1.057) | 0.163 |
| Hip replacement | 78/2930 | 0.827(0.647-1.058) | 0.131 | 0.906(0.693-1.185) | 0.473 |
| Knee replacement | 112/3904 | 0.833(0.673-1.030) | 0.092 | 0.857(0.677-1.086) | 0.202 |
| **Male** | | | | | |
| **Non-OA** | 1914/216547 |  | | | |
| **OA** | 718/38362 | **1.125(1.031-1.226)** | **0.008** | **1.130(1.023-1.248)** | **0.016** |
| **Site of OA** |  | | | | |
| Hand OA | 60/2905 | 1.085(0.839-1.403) | 0.536 | 0.885(0.641-1.223) | 0.460 |
| Hip OA | 92/3811 | 1.197(0.971-1.477) | 0.093 | **1.306(1.034-1.649)** | **0.025** |
| Knee OA | 156/7059 | **1.269(1.077-1.494)** | **0.004** | **1.259(1.038-1.529)** | **0.020** |
| **Drug** |  |  |  |  |  |
| **Nonuse ^c^** | 379/20379 |  |  |  |  |
| **Drug** | 158/10666 | **0.700(0.581-0.843)** | **<0.001** | **0.692(0.575-0.834)** | **<0.001** |
| **Drug_kind** |  |  |  |  |  |
| NSAID | 103/6871 | **0.692(0.557-0.860)** | **0.001** | **0.683(0.549-0.850)** | **0.001** |
| Opioid | 111/7449 | **0.674(0.546-0.833)** | **<0.001** | **0.670(0.542-0.828)** | **<0.001** |
| Glucosamine | 21/830 | 1.104(0.712-1.714) | 0.658 | 1.107(0.713-1.718) | 0.651 |
| Steroids injected | 46/2894 | **0.727(0.535-0.988)** | **0.041** | **0.704(0.518-0.957)** | **0.025** |
| **Surgery** |  |  |  |  |  |
| **Non-surgery** | 358/17195 |  |  |  |  |
| **OA surgery** | 112/3904 | **0.772(0.657-0.908)** | **0.002** | **0.808(0.672-0.972)** | **0.024** |
| Hip replacement | 94/4687 | **0.755(0.604-0.942)** | **0.013** | 0.803(0.627-1.028) | 0.082 |
| Knee replacement | 107/4768 | 0.815(0.660-1.005) | 0.056 | 0.785(0.612-1.009) | 0.059 |

The results are derived from Cox proportional hazard regression models in two models. Bold indicates statistical significance (P value<0.05).

Model 2 was additionally controlled for APOE ε4, ethnicity, education, TPA, BMI, smoking and alcohol than model 1.

Abbreviation: OA, osteoarthritis; ACD, all-cause dementia; AD, Alzheimer’s disease; VD, vascular dementia; APOE, Apolipoprotein E; BMI, body mass index; TPA, total physical activity; HR, Hazard ratio; CI, Confidence interval; NSAID, nonsteroidal anti-inflammatory drugs.

#### Supplementary Table 6. Association of different parts of osteoarthritis with therapy and dementia in APOE ε4 subgroups.

|  | **Number of incident dementia/ Number of participants** | **Model 1**  **(Gender+Age)** | | **Model 2**  **(+APOE ε4+Ethnicity+Education+TPA**  **+BMI+Smoking+Alcohol)** | |
| --- | --- | --- | --- | --- | --- |
|  |  | **HR (95%CI)** | **P value** | **HR (95%CI)** | **P value** |
| **APOE ε4-** | | | | | |
| **Non-OA** | 1674/244223 |  | | | |
| **OA** | 529/40147 | **1.183(1.072-1.306)** | **0.001** | 1.111(1.00001-1.235) | 0.050 |
| **Site of OA** |  | | | | |
| Hand OA | 34/2610 | 1.086(0.773-1.527) | 0.634 | 0.915(0.624-1.341) | 0.648 |
| Hip OA | 75/4486 | 1.274(1.010-1.607) | 0.041 | 1.275(1.0002-1.625) | 0.0498 |
| Knee OA | 145/8787 | **1.393(1.175-1.651)** | **<0.001** | **1.317(1.098-1.580)** | **0.003** |
| **Drug** |  |  |  |  |  |
| **Nonuse ^c^** | 327/24484 |  |  |  |  |
| **Drug** | 130/12864 | **0.673(0.549-0.825)** | **<0.001** | **0.668(0.545-0.819)** | **<0.001** |
| **Drug_kind** |  |  |  |  |  |
| NSAID | 76/8271 | **0.608(0.474-0.780)** | **<0.001** | **0.603(0.469-0.773)** | **<0.001** |
| Opioid | 88/8999 | **0.630(0.498-0.798)** | **<0.001** | **0.623(0.492-0.789)** | **<0.001** |
| Glucosamine | 18/920 | 1.164(0.724-1.871) | 0.532 | 1.160(0.721-1.865) | 0.541 |
| Steroids injected | 50/3454 | 0.940(0.698-1.266) | 0.684 | 0.918(0.681-1.237) | 0.573 |
| **Surgery** |  |  |  |  |  |
| **Non-surgery** | 344/28400 |  |  |  |  |
| **OA surgery** | 167/11491 | **0.776(0.644-0.935)** | **0.008** | **0.814(0.668-0.992)** | **0.041** |
| Hip replacement | 70/5007 | 0.776(0.599-1.005) | 0.055 | 0.831(0.634-1.089) | 0.179 |
| Knee replacement | 83/5553 | **0.768(0.603-0.977)** | **0.032** | 0.779(0.604-1.005) | 0.055 |
| **APOE ε4+** | | | | | |
| **Non-OA** | 1973/98511 |  | | | |
| **OA** | 602/15663 | 1.089(0.993-1.193) | 0.070 | **1.118(1.015-1.232)** | **0.024** |
| **Site of OA** |  | | | | |
| Hand OA | 45/1018 | 1.072(0.797-1.442) | 0.645 | 1.082(0.790-1.482) | 0.625 |
| Hip OA | 92/1681 | **1.310(1.062-1.616)** | **0.012** | **1.394(1.120-1.735)** | **0.003** |
| Knee OA | 160/3511 | **1.247(1.061-1.465)** | **0.007** | **1.331(1.126-1.575)** | **0.001** |
| **Drug** |  |  |  |  |  |
| **Nonuse ^c^** | 365/9549 |  |  |  |  |
| **Drug** | 173/4959 | **0.812(0.678-0.974)** | **0.024** | **0.814(0.679-0.976)** | **0.026** |
| **Drug_kind** |  |  |  |  |  |
| NSAID | 113/3174 | 0.820(0.664-1.013) | 0.066 | 0.822(0.666-1.016) | 0.069 |
| Opioid | 132/3432 | 0.853(0.699-1.041) | 0.118 | 0.857(0.702-1.046) | 0.128 |
| Glucosamine | 15/371 | 0.878(0.524-1.472) | 0.622 | 0.875(0.522-1.467) | 0.613 |
| Steroids injected | 54/1338 | 0.907(0.681-1.207) | 0.503 | 0.901(0.676-1.200) | 0.476 |
| **Surgery** |  |  |  |  |  |
| **Non-surgery** | 384/11150 |  |  |  |  |
| **OA surgery** | 202/4419 | 0.835(0.703-0.991) | 0.039 | 0.862(0.720-1.033) | 0.107 |
| Hip replacement | 86/1944 | 0.835(0.660-1.056) | 0.132 | 0.868(0.679-1.109) | 0.256 |
| Knee replacement | 103/2126 | 0.850(0.682-1.059) | 0.146 | 0.864(0.684-1.091) | 0.219 |

The results are derived from Cox proportional hazard regression models in two models. Bold indicates statistical significance (P value<0.05).

Model 2 was additionally controlled for APOE ε4, ethnicity, education, TPA, BMI, smoking and alcohol than model 1.

Abbreviation: OA, osteoarthritis; ACD, all-cause dementia; AD, Alzheimer’s disease; VD, vascular dementia; APOE, Apolipoprotein E; BMI, body mass index; TPA, total physical activity; HR, Hazard ratio; CI, Confidence interval; NSAID, nonsteroidal anti-inflammatory drugs.

#### Supplementary Table 7. The association of OA and dementia in subgroups of osteoarthritis duration.

|  | **Number of incident dementia/ Number of participants** | **Unmatched** | | **Matched**  **(1:4)** | |  |
| --- | --- | --- | --- | --- | --- | --- |
|  |  | **HR (95%CI)** | **P value** | **HR (95%CI)** | **P value** |  |
| **Duration < 5 years** | | | | | |  |
| **Non-OA** | 4085/384538 |  | | | |  |
| **OA** | 37/2609 | **45.058(29.179-69.578)** | **<0.001** | **4.228(21.689-82.433)** | **<0.001** |  |
| **Site of OA** |  | | | | |  |
| Hand OA | 2/68 | **40.938(5.744-291.802)** | **<0.001** | **-** | **-** |  |
| Hip OA | 4/287 | **62.825(23.404-168.643)** | **<0.001** | **-** | **-** |  |
| Knee OA | 11/576 | **66.694(33.007-134.761)** | **<0.001** | **-** | **-** |  |
|  | **Number of incident dementia/ Number of participants** | **Model 1**  **(Gender+Age)** | | **Model 2**  **(+APOE ε4+Ethnicity+Education+TPA**  **+BMI+Smoking+Alcohol)** | |  |
|  |  | **HR (95%CI)** | **P value** | **HR (95%CI)** | **P value** |  |
| **Duration ≥ 5 years** | | | | | |  |
| **Non-OA** | 4330/403379 |  | | | |  |
| **OA** | 1260/60472 | **1.128(1.059-1.201)** | **<0.001** | **1.138(1.060-1.222)** | **<0.001** |  |
| **Site of OA** |  | | | | |  |
| Hand OA | 91/4019 | 1.086(0.882-1.338) | 0.435 | 0.998(0.782-1.275) | 0.989 |  |
| Hip OA | 182/6573 | **1.212(1.044-1.406)** | **0.011** | **1.301(1.104-1.534)** | **0.002** |  |
| Knee OA | 352/13579 | **1.284(1.152-1.432)** | **<0.001** | **1.288(1.137-1.459)** | **<0.001** |  |

The results are derived from Cox proportional hazard regression models in two models. Bold indicates statistical significance (P value<0.05).

Model 2 was additionally controlled for APOE ε4, ethnicity, education, TPA, BMI, smoking and alcohol than model 1.

Abbreviation: OA, osteoarthritis; ACD, all-cause dementia; AD, Alzheimer’s disease; VD, vascular dementia; APOE, Apolipoprotein E; BMI, body mass index; TPA, total physical activity; HR, Hazard ratio; CI, Confidence interval.

#### Supplementary Table 8. The association of OA with therapy as exposures with subsequent dementia outcome in subgroups of BMI.

|  | **Number of incident dementia/ Number of participants** | **Model 1**  **(Gender+Age)** | | **Model 2**  **(+APOE ε4+Ethnicity+Education+TPA**  **+BMI+Smoking+Alcohol)** | |
| --- | --- | --- | --- | --- | --- |
|  |  | **HR (95%CI)** | **P value** | **HR (95%CI)** | **P value** |
| **18.5≤BMI<25** | | | | | |
| **Non-OA** | 1372/134627 |  | | | |
| **OA** | 271/13551 | 1.112(0.975-1.268) | 0.114 | 1.136(0.983-1.313) | 0.084 |
| **Site of OA** |  | | | | |
| Hand OA | 17/881 | 0.974(0.603-1.572) | 0.913 | 1.080(0.658-1.770) | 0.761 |
| Hip OA | 48/1432 | **1.642(1.230-2.191)** | **0.001** | **1.648(1.196-2.270)** | **0.002** |
| Knee OA | 48/2001 | 1.302(0.976-1.737) | 0.073 | 1.313(0.953-1.808) | 0.096 |
| **Drug** |  |  |  |  |  |
| **Nonuse ^c^** | 154/7565 |  |  |  |  |
| **Drug** | 64/3870 | **0.694(0.518-0.929)** | **0.014** | **0.690(0.516-0.925)** | **0.013** |
| **Drug_kind** |  |  |  |  |  |
| NSAID | 36/2544 | **0.595(0.414-0.855)** | **0.005** | **0.597(0.415-0.857)** | **0.005** |
| Opioid | 49/2506 | 0.796(0.577-1.098) | 0.165 | 0.792(0.574-1.093) | 0.156 |
| Glucosamine | 9/288 | 1.089(0.555-2.134) | 0.805 | 1.092(0.556-2.144) | 0.798 |
| Steroids injected | 17/868 | 0.787(0.477-1.300) | 0.350 | 0.811(0.491-1.340) | 0.414 |
| **Surgery** |  |  |  |  |  |
| **Non-surgery** | 193/10858 |  |  |  |  |
| **OA surgery** | 69/2615 | 0.942(0.715-1.241) | 0.670 | 0.944(0.698-1.277) | 0.710 |
| Hip replacement | 37/1568 | 0.913(0.641-1.298) | 0.611 | 0.846(0.568-1.261) | 0.412 |
| Knee replacement | 24/788 | 0.933(0.609-1.429) | 0.749 | 1.013(0.644-1.594) | 0.955 |
| **25≤BMI<30** | | | | | |
| **Non-OA** | 1810/171210 |  | | | |
| **OA** | 502/25783 | 1.096(0.992-1.211) | 0.073 | 1.076(0.963-1.203) | 0.196 |
| **Site of OA** |  | | | | |
| Hand OA | 30/1579 | 0.958(0.667-1.376) | 0.817 | 0.907(0.605-1.359) | 0.634 |
| Hip OA | 71/2924 | 1.130(0.891-1.434) | 0.312 | 1.229(0.948-1.592) | 0.120 |
| Knee OA | 138/5589 | **1.363(1.146-1.622)** | **<0.001** | **1.300(1.068-1.582)** | **0.009** |
| **Drug** |  |  |  |  |  |
| **Nonuse ^c^** | 281/14237 |  |  |  |  |
| **Drug** | 113/7366 | **0.690(0.554-0.858)** | **0.001** | **0.691(0.555-0.860)** | **0.001** |
| **Drug_kind** |  |  |  |  |  |
| NSAID | 74/4771 | **0.690(0.534-0.891)** | **0.004** | **0.690(0.534-0.892)** | **0.005** |
| Opioid | 81/5159 | **0.674(0.526-0.863)** | **0.002** | **0.669(0.523-0.857)** | **0.001** |
| Glucosamine | 13/486 | 1.089(0.624-1.900) | 0.763 | 1.120(0.642-1.955) | 0.689 |
| Steroids injected | 32/1902 | 0.737(0.511-1.062) | 0.101 | 0.720(0.499-1.039) | 0.079 |
| **Surgery** |  |  |  |  |  |
| **Non-surgery** | 329/18734 |  |  |  |  |
| **OA surgery** | 158/6884 | 0.818(0.676-0.991) | 0.040 | 0.874(0.707-1.080) | 0.213 |
| Hip replacement | 70/399 | 0.791(0.610-1.025) | 0.076 | 0.914(0.692-1.208) | 0.527 |
| Knee replacement | 77/3082 | 0.860(0.670-1.104) | 0.236 | 0.825(0.621-1.096) | 0.184 |
| **BMI≥30** | | | | | |
| **Non-OA** | 1046/92823 |  |  |  |  |
| **OA** | 497/23134 | **1.146(1.029-1.277)** | **0.013** | **1.142(1.011-1.291)** | **0.033** |
| **Site of OA** |  |  |  |  |  |
| Hand OA | 43/1569 | 1.325(0.975-1.801) | 0.072 | 1.065(0.724-1.566) | 0.750 |
| Hip OA | 64/2415 | 1.200(0.931-1.546) | 0.159 | 1.265(0.961-1.666) | 0.094 |
| Knee OA | 171/6414 | **1.332(1.132-1.567)** | **0.001** | **1.314(1.092-1.580)** | **0.004** |
| **Drug** |  |  |  |  |  |
| **Nonuse ^c^** | 254/12126 |  |  |  |  |
| **Drug** | 124/6542 | 0.823(0.664-1.020) | 0.076 | 0.828(0.668-1.026) | 0.085 |
| **Drug_kind** |  |  |  |  |  |
| NSAID | 77/4100 | 0.807(0.626-1.042) | 0.100 | 0.813(0.630-1.050) | 0.112 |
| Opioid | 89/4737 | 0.794(0.624-1.011) | 0.062 | 0.801(0.629-1.021) | 0.073 |
| Glucosamine | 11/515 | 0.857(0.468-1.567) | 0.615 | 0.833(0.455-1.524) | 0.553 |
| Steroids injected | 55/2017 | 1.165(0.870-1.560) | 0.305 | 1.153(0.861-1.543) | 0.341 |
| **Surgery** |  |  |  |  |  |
| **Non-surgery** | 294/14938 |  |  |  |  |
| **OA surgery** | 187/8050 | **0.737(0.613-0.887)** | **0.001** | **0.769(0.625-0.947)** | **0.013** |
| Hip replacement | 61/2744 | 0.720(0.545-0.950) | 0.020 | 0.780(0.576-1.057) | 0.109 |
| Knee replacement | 117/4737 | **0.772(0.622-0.959)** | **0.019** | **0.783(0.613-0.999)** | **0.049** |

The results are derived from Cox proportional hazard regression models in two models. Bold indicates statistical significance (P value<0.05).

Model 2 was additionally controlled for APOE ε4, ethnicity, education, TPA, BMI, smoking and alcohol than model 1.

Abbreviation: OA, osteoarthritis; ACD, all-cause dementia; AD, Alzheimer’s disease; VD, vascular dementia; APOE, Apolipoprotein E; BMI, body mass index; TPA, total physical activity; HR, Hazard ratio; CI, Confidence interval; NSAID, nonsteroidal anti-inflammatory drugs.

#### Supplementary Table 9. The association of OA with therapy as exposures with subsequent dementia outcome in subgroups of TPA.

|  | **Number of incident dementia/ Number of participants** | **Model 1**  **(Gender+Age)** | | **Model 2**  **(+APOE ε4+Ethnicity+Education+TPA**  **+BMI+Smoking+Alcohol)** | |
| --- | --- | --- | --- | --- | --- |
|  |  | **HR (95%CI)** | **P value** | **HR (95%CI)** | **P value** |
| **TPA<600** | | | | | |
| **Non-OA** | 1092/92651 |  | | | |
| **OA** | 370/16478 | 1.102(0.978-1.242) | 0.111 | 1.112(0.972-1.271) | 0.121 |
| **Site of OA** |  | | | | |
| Hand OA | 20/1130 | 0.791(0.508-1.233) | 0.301 | 0.621(0.366-1.056) | 0.079 |
| Hip OA | 42/1791 | 0.993(0.729-1.352) | 0.962 | 1.104(0.795-1.532) | 0.555 |
| Knee OA | 114/3630 | **1.423(1.173-1.728)** | **<0.001** | **1.295(1.033-1.623)** | **0.025** |
| **Drug** |  |  |  |  |  |
| **Nonuse ^c^** | 223/9421 |  |  |  |  |
| **Drug** | 81/4781 | **0.638(0.495-0.823)** | **0.001** | **0.638(0.494-0.823)** | **0.001** |
| **Drug_kind** |  |  |  |  |  |
| NSAID | 53/3049 | **0.650(0.482-0.877)** | **0.005** | **0.651(0.482-0.878)** | **0.005** |
| Opioid | 64/3366 | **0.695(0.526-0.917)** | **0.010** | **0.696(0.527-0.920)** | **0.011** |
| Glucosamine | 9/380 | 0.808(0.415-1.574) | 0.531 | 0.790(0.406-1.539) | 0.489 |
| Steroids injected | 32/1376 | 0.819(0.566-1.187) | 0.292 | 0.785(0.541-1.137) | 0.200 |
| **Surgery** |  |  |  |  |  |
| **Non-surgery** | 247/11490 |  |  |  |  |
| **OA surgery** | 112/4858 | 0.704(0.563-0.882) | 0.002 | 0.720(0.564-0.919) | 0.008 |
| Hip replacement | 44/2052 | 0.685(0.497-0.946) | 0.022 | 0.730(0.517-1.030) | 0.073 |
| Knee replacement | 61/2420 | 0.737(0.556-0.978) | 0.034 | 0.700(0.513-0.954) | 0.024 |
| **600≤TPA<3000** | | | | | |
| **Non-OA** | 1898/183409 |  | | | |
| **OA** | 503/26026 | 1.058(0.959-1.169) | 0.262 | 1.061(0.952-1.182) | 0.287 |
| **Site of OA** |  | | | | |
| Hand OA | 38/1583 | 1.165(0.844-1.607) | 0.354 | 1.163(0.819-1.653) | 0.399 |
| Hip OA | 85/2934 | **1.319(1.061-1.641)** | **0.013** | **1.416(1.127-1.780)** | **0.003** |
| Knee OA | 139/5648 | **1.282(1.079-1.524)** | **0.005** | **1.350(1.120-1.627)** | **0.002** |
| **Drug** |  |  |  |  |  |
| **Nonuse ^c^** | 294/15112 |  |  |  |  |
| **Drug** | 129/7829 | **0.747(0.607-0.918)** | **0.006** | **0.741(0.603-0.912)** | **0.005** |
| **Drug_kind** |  |  |  |  |  |
| NSAID | 79/5038 | **0.706(0.550-0.905)** | **0.006** | **0.698(0.545-0.896)** | **0.005** |
| Opioid | 82/5387 | **0.655(0.513-0.837)** | **0.001** | **0.649(0.508-0.829)** | **0.001** |
| Glucosamine | 16/557 | 1.159(0.700-1.917) | 0.567 | 1.152(0.696-1.907) | 0.582 |
| Steroids injected | 40/1995 | 0.900(0.647-1.252) | 0.532 | 0.896(0.643-1.247) | 0.514 |
| **Surgery** |  |  |  |  |  |
| **Non-surgery** | 306/18750 |  |  |  |  |
| **OA surgery** | 179/7121 | 0.967(0.803-1.164) | 0.720 | 1.037(0.848-1.268) | 0.724 |
| Hip replacement | 78/3202 | 0.963(0.750-1.237) | 0.770 | 1.030(0.791-1.342) | 0.826 |
| Knee replacement | 89/3380 | 0.988(0.779-1.252) | 0.917 | 1.033(0.795-1.344) | 0.806 |
| **TPA≥3000** | | | | | |
| **Non-OA** | 1009/105555 |  |  |  |  |
| **OA** | 321/16460 | **1.228(1.082-1.393)** | **0.001** | **1.193(1.042-1.366)** | **0.011** |
| **Site of OA** |  |  |  |  |  |
| Hand OA | 23/1035 | 1.271(0.840-1.923) | 0.257 | 1.242(0.804-1.918) | 0.329 |
| Hip OA | 43/1716 | **1.360(1.002-1.846)** | **0.049** | **1.399(1.010-1.938)** | **0.043** |
| Knee OA | 85/3925 | **1.336(1.070-1.667)** | **0.011** | **1.283(1.009-1.630)** | **0.042** |
| **Drug** |  |  |  |  |  |
| **Nonuse ^c^** | 175/9500 |  |  |  |  |
| **Drug** | 93/5213 | 0.870(0.676-1.119) | 0.277 | 0.879(0.683-1.131) | 0.317 |
| **Drug_kind** |  |  |  |  |  |
| NSAID | 57/3358 | 0.816(0.605-1.100) | 0.182 | 0.821(0.609-1.108) | 0.198 |
| Opioid | 74/3678 | 0.955(0.728-1.253) | 0.739 | 0.970(0.739-1.274) | 0.826 |
| Glucosamine | 8/354 | 1.014(0.499-2.059) | 0.970 | 1.048(0.515-2.131) | 0.898 |
| Steroids injected | 32/1421 | 1.090(0.748-1.590) | 0.653 | 1.089(0.746-1.590) | 0.660 |
| **Surgery** |  |  |  |  |  |
| **Non-surgery** | 211/11851 |  |  |  |  |
| **OA surgery** | 100/4525 | **0.736(0.579-0.936)** | **0.012** | **0.720(0.555-0.933)** | **0.013** |
| Hip replacement | 39/1884 | 0.723(0.513-1.019) | 0.064 | 0.731(0.506-1.056) | 0.095 |
| Knee replacement | 54/2264 | 0.756(0.559-1.022) | 0.069 | 0.720(0.515-1.007) | 0.055 |

## Sensitivity analysis

#### Supplementary Table 10. The association of OA and dementia, after excluding participants diagnosed with self-report.

|  | **Number of incident dementia/ Number of participants** | **Model 1**  **(Gender+Age)** | | **Model 2**  **(+APOE ε4+Ethnicity+Education+TPA**  **+BMI+Smoking+Alcohol)** | |
| --- | --- | --- | --- | --- | --- |
|  |  | **HR (95%CI)** | **P value** | **HR (95%CI)** | **P value** |
| **ACD** | | | | | |
| **Non-OA** | 4324/401476 |  | | | |
| **OA** | 549/24420 | **1.182(1.081-1.292)** | **<0.001** | **1.214(1.098-1.342)** | **<0.001** |
| **Site of OA** |  | | | | |
| Hand OA | 60/3445 | 1.227(0.951-1.584) | 0.116 | 1.076(0.790-1.465) | 0.641 |
| Hip OA | 103/3972 | 1.158(0.952-1.408) | 0.142 | **1.297(1.048-1.606)** | **0.017** |
| Knee OA | 230/9269 | **1.330(1.165-1.519)** | **<0.001** | **1.364(1.173-1.587)** | **<0.001** |
| **Drug** |  |  |  |  |  |
| **Nonuse ^c^** | 281/11876 |  |  |  |  |
| **Drug** | 133/7930 | **0.609(0.495-0.749)** | **<0.001** | **0.609(0.496-0.749)** | **<0.001** |
| **Drug_kind** |  |  |  |  |  |
| NSAID | 79/4581 | **0.632(0.492-0.812)** | **<0.001** | **0.630(0.491-0.810)** | **<0.001** |
| Opioid | 93/5246 | **0.622(0.492-0.787)** | **<0.001** | **0.620(0.490-0.784)** | **<0.001** |
| Glucosamine | 15/552 | 0.933(0.555-1.569) | 0.795 | 0.952(0.566-1.602) | 0.854 |
| Steroids injected | 43/2072 | 0.736(0.534-1.015) | 0.062 | **0.723(0.524-0.997)** | **0.048** |
| **Surgery** |  |  |  |  |  |
| **Non-surgery** | 329/16783 |  |  |  |  |
| **OA surgery** | 202/7348 | 0.876(0.734-1.046) | 0.143 | 0.914(0.749-1.117) | 0.380 |
| Hip surgery | 81/3185 | 0.840(0.657-1.073) | 0.162 | 0.903(0.689-1.183) | 0.457 |
| Knee surgery | 104/3501 | 0.908(0.727-1.135) | 0.397 | 0.912(0.706-1.179) | 0.483 |
| **AD** | | | | | |
| **Non-OA** | 1862/399014 |  |  |  |  |
| **OA** | 106/9145 | **1.219(1.069-1.392)** | **0.003** | **1.292(1.114-1.498)** | **0.001** |
| **Site of OA** |  |  |  |  |  |
| Hand OA | 12/336 | **1.426(1.005-2.024)** | **0.047** | 1.223(0.794-1.882) | 0.362 |
| Hip OA | 12/397 | 1.080(0.798-1.462) | 0.617 | 1.209(0.864-1.692) | 0.269 |
| Knee OA | 106/9145 | **1.407(1.156-1.711)** | **<0.001** | **1.470(1.175-1.840)** | **0.001** |
| **Drug** |  |  |  |  |  |
| **Nonuse ^c^** | 133/11728 |  |  |  |  |
| **Drug** | 57/7854 | **0.550(0.403-0.750)** | **<0.001** | **0.545(0.399-0.743)** | **<0.001** |
| **Drug_kind** |  |  |  |  |  |
| NSAID | 32/4534 | **0.542(0.368-0.793)** | **0.002** | **0.540(0.367-0.795)** | **0.002** |
| Opioid | 38/5191 | **0.537(0.374-0.771)** | **0.001** | **0.530(0.370-0.761)** | **0.001** |
| Glucosamine | 6/543 | 0.795(0.351-1.802) | 0.583 | 0.832(0.367-1.886) | 0.659 |
| Steroids injected | 18/2047 | 0.645(0.394-1.055) | 0.081 | 0.637(0.389-1.044) | 0.073 |
| **Surgery** |  |  |  |  |  |
| **Non-surgery** | 158/16612 |  |  |  |  |
| **OA surgery** | 86/7232 | **0.767(0.588-1.000)** | **0.050** | 0.845(0.626-1.139) | 0.268 |
| Hip replacement | 33/3137 | 0.700(0.480-1.021) | 0.064 | 0.773(0.509-1.174) | 0.227 |
| Knee replacement | 47/3444 | **0.848(0.610-1.178)** | **0.326** | 0.946(0.648-1.381) | 0.772 |
| **VD** | | | | | |
| **Non-OA** | 996/398148 |  |  |  |  |
| **OA** | 135/24006 | **1.245(1.040-1.492)** | **0.017** | **1.230(1.004-1.507)** | **0.046** |
| **Site of OA** |  |  |  |  |  |
| Hand OA | 17/2402 | **1.545(0.956-2.497)** | **0.076** | 1.471(0.849-2.548) | 0.169 |
| Hip OA | 22/3891 | 1.044(0.684-1.594) | 0.842 | 1.116(0.699-1.783) | 0.646 |
| Knee OA | 51/9090 | 1.244(0.939-1.649) | 0.129 | 1.290(0.949-1.753) | 0.104 |
| **Drug** |  |  |  |  |  |
| **Nonuse ^c^** | 69/11664 |  |  |  |  |
| **Drug** | 33/7830 | **0.613(0.405-0.929)** | **0.021** | **0.619(0.409-0.937)** | **0.024** |
| **Drug_kind** |  |  |  |  |  |
| NSAID | 15/4517 | **0.489(0.280-0.855)** | **0.012** | **0.502(0.287-0.878)** | **0.016** |
| Opioid | 21/5174 | **0.575(0.353-0.938)** | **0.027** | **0.576(0.353-0.940)** | **0.027** |
| Glucosamine | 5/542 | 1.266(0.510-3.139) | 0.611 | 1.282(0.517-3.182) | 0.592 |
| Steroids injected | 6/2035 | **0.423(0.184-0.975)** | **0.044** | **0.402(0.174-0.926)** | **0.032** |
| **Surgery** |  |  |  |  |  |
| **Non-surgery** | 80/16534 |  |  |  |  |
| **OA surgery** | 52/7198 | 0.932(0.655-1.326) | 0.695 | 0.860(0.579-1.279) | 0.457 |
| Hip replacement | 19/3123 | 0.816(0.494-1.350) | 0.430 | 0.766(0.429-1.370) | 0.369 |
| Knee replacement | 31/3428 | 1.102(0.725-1.675) | 0.650 | 0.968(0.604-1.550) | 0.891 |

The results are derived from Cox proportional hazard regression models in two models. Bold indicates statistical significance (P value<0.05).

Model 2 was additionally controlled for APOE ε4, ethnicity, education, TPA, BMI, smoking and alcohol than model 1.

Abbreviation: OA, osteoarthritis; ACD, all-cause dementia; AD, Alzheimer’s disease; VD, vascular dementia; APOE, Apolipoprotein E; BMI, body mass index; TPA, total physical activity; HR, Hazard ratio; CI, Confidence interval; NSAID, nonsteroidal anti-inflammatory drugs.

#### Supplementary Table 11. The association of OA and dementia, after excluding inflammatory arthritis (rheumatoid arthritis, psoriatic arthritis, enteropathic arthritis, gout, ankylosing spondylitis and other inflammatory spondylopathies)

|  | **Number of incident dementia/ Number of participants** | **Model 1**  **(Gender+Age)** | | **Model 2**  **(+APOE ε4+Ethnic+Education+TPA**  **+BMI+Smoking+Alcohol)** | |
| --- | --- | --- | --- | --- | --- |
|  |  | **HR (95%CI)** | **P value** | **HR (95%CI)** | **P value** |
| **ACD** | | | | | |
| **Non-OA** | 4062/388934 |  | | | |
| **OA** | 1172/58105 | **1.116(1.045-1.191)** | **0.001** | **1.115(1.035-1.202)** | **0.004** |
| **Site of OA** |  | | | | |
| Hand OA | 78/3640 | 1.055(0.843-1.320) | 0.642 | 1.011(0.780-1.309) | 0.937 |
| Hip OA | 173/6276 | **1.281(1.099-1.492)** | **0.002** | **1.382(1.168-1.636)** | **<0.001** |
| Knee OA | 329/12825 | **1.358(1.213-1.519)** | **<0.001** | **1.343(1.180-1.529)** | **<0.001** |
| **Drug** |  |  |  |  |  |
| **Nonuse ^c^** | 640/31523 |  |  |  |  |
| **Drug** | 258/16298 | **0.694 (0.601-0.802)** | **<0.001** | **0.693(0.600-0.801)** | **<0.001** |
| **Drug_kind** |  |  |  |  |  |
| NSAID | 166/10524 | **0.686 (0.579-0.814)** | **<0.001** | **0.687(0.577-0.815)** | **<0.001** |
| Opioid | 183/11319 | **0.680(0.577-0.801)** | **<0.001** | **0.679(0.576-0.800)** | **<0.001** |
| Glucosamine | 23/1168 | 0.763(0.503-1.156) | 0.202 | 0.761(0.502-1.154) | 0.199 |
| Steroids injected | 89/4297 | 0.882(0.707-1.101) | 0.267 | 0.860(0.688-1.074) | 0.182 |
| **Surgery** |  |  |  |  |  |
| **Non-surgery** | 747/41745 |  |  |  |  |
| **OA surgery** | 386/16048 | **0.832(0.735-0.942)** | **0.004** | 0.870(0.757-1.000) | 0.051 |
| Hip replacement | 160/7048 | **0.814(0.686-0.966)** | **0.019** | 0.869(0.718-1.050) | 0.146 |
| Knee replacement | 199/7766 | **0.854(0.730-1.000)** | **0.049** | 0.850(0.710-1.018) | 0.078 |
| **AD** | | | | | |
| **Non-OA** | 1746/386618 |  |  |  |  |
| **OA** | 522/57455 | **1.1108(1.004-1.222)** | **0.043** | **1.126(1.007-1.260)** | **0.037** |
| **Site of OA** |  |  |  |  |  |
| Hand OA | 38/3600 | 1.111(0.805-1.534) | 0.521 | 1.069(0.734-1.556) | 0.729 |
| Hip OA | 70/6173 | 1.152(0.907-1.464) | 0.246 | 1.274(0.976-1.663) | 0.075 |
| Knee OA | 150/12646 | **1.409(1.192-1.666)** | **<0.001** | **1.433(1.182-1.737)** | **<0.001** |
| **Drug** |  |  |  |  |  |
| **Nonuse ^c^** | 293/31176 |  |  |  |  |
| **Drug** | 105/16145 | **0.616(0.493-0.770)** | **<0.001** | **0.613(0.490-0.766)** | **<0.001** |
| **Drug_kind** |  |  |  |  |  |
| NSAID | 73/10431 | **0.658(0.509-0.850)** | **0.001** | **0.658(0.509-0.850)** | **0.001** |
| Opioid | 74/11210 | **0.598(0.463-0.771)** | **<0.001** | **0.596(0.462-0.770)** | **<0.001** |
| Glucosamine | 5/1150 | **0.359(0.148-0.870)** | **0.023** | **0.364(0.150-0.881)** | **0.025** |
| Steroids injected | 36/4244 | 0.776(0.549-1.098) | 0.152 | 0.754(0.533-1.067) | 0.111 |
| **Surgery** |  |  |  |  |  |
| **Non-surgery** | 347/41345 |  |  |  |  |
| **OA surgery** | 157/15819 | **0.715(0.591-0.864)** | **0.001** | **0.780(0.630-0.966)** | **0.023** |
| Hip replacement | 61/6949 | **0.650(0.495-0.854)** | **0.002** | **0.710(0.524-0.961)** | **0.027** |
| Knee replacement | 85/7652 | **0.768(0.605-0.975)** | **0.030** | 0.817(0.622-1.074) | 0.147 |
| **VD** | | | | | |
| **Non-OA** | 931/385803 |  |  |  |  |
| **OA** | 72/12568 | **1.162(1.015-1.330)** | **0.030** | 1.111(0.951-1.297) | 0.184 |
| **Site of OA** |  |  |  |  |  |
| Hand OA | 7/602 | 1.269(0.782-2.059) | 0.367 | 1.269(0.782-2.059) | 0.335 |
| Hip OA | 6/653 | **1.363(0.959-1.936)** | **0.043** | 1.363(0.959-1.936) | 0.084 |
| Knee OA | 72/12568 | 1.210(0.922-1.589) | 0.050 | 1.210(0.922-1.589) | 0.170 |
| **Drug** |  |  |  |  |  |
| **Nonuse ^c^** | 143/31026 |  |  |  |  |
| **Drug** | 65/16105 | 0.773(0.577-1.037) | 0.085 | 0.773(0.577-1.037) | 0.086 |
| **Drug_kind** |  |  |  |  |  |
| NSAID | 41/10399 | 0.757(0.535-1.072) | 0.108 | 0.757(0.535-1.072) | 0.117 |
| Opioid | 43/11179 | **0.704(0.500-0.990)** | **0.046** | **0.704(0.500-0.990)** | **0.044** |
| Glucosamine | 7/1152 | 1.018(0.477-2.176) | 0.945 | 1.018(0.477-2.176) | 0.963 |
| Steroids injected | 23/4231 | 0.951(0.612-1.479) | 0.986 | 0.951(0.612-1.479) | 0.824 |
| **Surgery** |  |  |  |  |  |
| **Non-surgery** | 174/41172 |  |  |  |  |
| **OA surgery** | 97/15759 | 0.854(0.643-1.136) | 0.381 | 0.854(0.643-1.136) | 0.279 |
| Hip replacement | 41/6929 | 0.858(0.579-1.271) | 0.491 | 0.858(0.579-1.271) | 0.445 |
| Knee replacement | 52/7619 | 0.842(0.590-1.201) | 0.743 | 0.842(0.590-1.201) | 0.342 |

The results are derived from Cox proportional hazard regression models in two models. Bold indicates statistical significance (P value<0.05).

Model 2 was additionally controlled for APOE ε4, ethnic, education, TPA, BMI, smoking and alcohol than model 1.

Abbreviation: OA, osteoarthritis; ACD, all-cause dementia; AD, Alzheimer’s disease; VD, vascular dementia; APOE, Apolipoprotein E; BMI, body mass index; TPA, total physical activity; HR, Hazard ratio; CI, Confidence interval;.

#### Supplementary Table 12. The association of osteoarthritis as exposures and subsequent dementia with competing risk model.

|  | **Number of incident dementia/ Number of participants** | **Model 1**  **(Gender+Age)** | | **Model 2**  **(+APOE ε4+Ethnicity+Education+TPA**  **+BMI+Smoking+Alcohol)** | |
| --- | --- | --- | --- | --- | --- |
|  |  | **HR (95%CI)** | **P value** | **HR (95%CI)** | **P value** |
| **ACD** | | | | | |
| **Non-OA** | 4330/403379 |  | | | |
| **OA** | 1297/63081 | **1.100(1.030-1.170)** | **0.004** | **1.143(1.964-1.229)** | **<0.001** |
| **Site of OA** |  | | | | |
| Hand OA | 93/4087 | 1.000(0.819-1.230) | 0.980 | 0.972(0.758-1.247) | 0.820 |
| Hip OA | 186/6860 | **1.080(1.080-1.090)** | **0.039** | **1.191(1.003-1.414)** | **0.046** |
| Knee OA | 363/14155 | **1.290(1.160-1.430)** | **<0.001** | **1.349(1.194-1.526)** | **<0.001** |
| **AD** | | | | | |
| **Non-OA** | 1862/400911 |  |  |  |  |
| **OA** | 576/62360 | **1.110(1.010-1.220)** | **0.028** | **1.175(1.056-1.308)** | **0.003** |
| **Site of OA** |  |  |  |  |  |
| Hand OA | 47/4041 | 1.130(0.850-1.510) | 0.390 | 1.062(0.737-1.531) | 0.750 |
| Hip OA | 77/6751 | 1.150(0.913-1.460) | 0.230 | 1.184(0.900-1.558) | 0.230 |
| Knee OA | 169/13961 | **1.430(1.220-1.670)** | **<0.001** | **1.473(1.228-1.766)** | **<0.001** |
| **VD** | | | | | |
| **Non-OA** | 998/400047 |  |  |  |  |
| **OA** | 314/62098 | **1.194(1.050-1.360)** | **0.007** | **1.222(1.054-1.416)** | **0.008** |
| **Site of OA** |  |  |  |  |  |
| Hand OA | 24/4018 | 1.204(0.799-1.810) | 0.380 | 1.194(0.746-1.910) | 0.460 |
| Hip OA | 46/6720 | 1.300(0.964-1.760) | 0.085 | 1.259(0.901-1.759) | 0.180 |
| Knee OA | 81/13873 | **1.270(1.012-1.600)** | **0.039** | **1.326(1.029-1.707)** | **0.029** |

The results are derived from Cox proportional hazard regression models in two models. Bold indicates statistical significance (P value<0.05).

Model 2 was additionally controlled for APOE ε4, ethnicity, education, TPA, BMI, smoking and alcohol than model 1.

Abbreviation: OA, osteoarthritis; ACD, all-cause dementia; AD, Alzheimer’s disease; VD, vascular dementia; APOE, Apolipoprotein E; BMI, body mass index; TPA, total physical activity; HR, Hazard ratio; CI, Confidence interval.

#### Supplementary Table 13. The association of OA and therapy as exposures with subsequent dementia outcome, using inverse probability censoring weights to adjust for potential selection bias.

|  | **Number of incident dementia/ Number of participants** | **Model 1**  **(Gender+Age)** | | **Model 2**  **(+APOE ε4+Ethnicity+Education+TPA**  **+BMI+Smoking+Alcohol)** | |
| --- | --- | --- | --- | --- | --- |
|  |  | **HR (95%CI)** | **P value** | **HR (95%CI)** | **P value** |
| **ACD** | | | | | |
| **Non-OA** | 4330/403379 |  | | | |
| **OA** | 1297/63081 | **1.139(1.059-1.225)** | **<0.001** | **1.112(1.032-1.199)** | **0.005** |
| **Site of OA** |  | | | | |
| Hand OA | 93/4087 | 1.082(0.823-1.422) | 0.575 | 1.034(0.767-1.395) | 0.825 |
| Hip OA | 186/6860 | 1.245(1.045-1.484) | 0.014 | **1.285(1.077-1.532)** | **0.005** |
| Knee OA | 363/14155 | **1.407(1.235-1.603)** | **<0.001** | **1.324(1.142-1.534)** | **<0.001** |
| **Drug** |  |  |  |  |  |
| **Nonuse ^c^** |  |  |  |  |  |
| **Drug** | 303/17823 | **0.745(0.651-0.853)** | **<0.001** | **0.747(0.652-0.855)** | **<0.001** |
| **Drug_kind** | 692/34033 |  |  |  |  |
| NSAID | 189/11445 | **0.719(0.612-0.844)** | **<0.001** | **0.720(0.613-0.846)** | **<0.001** |
| Opioid | 220/12431 | **0.623(0.493-0.788)** | **<0.001** | **0.620(0.491-0.784)** | **<0.001** |
| Glucosamine | 33/1291 | 0.979(0.687-1.394) | 0.905 | 0.986(0.693-1.402) | 0.936 |
| Steroids injected | 104/4792 | 0.938(0.761-1.153) | 0.540 | 0.918(0.745-1.131) | 0.419 |
| **Surgery** |  |  |  |  |  |
| **Non-surgery** | 836/44973 |  |  |  |  |
| **OA surgery** | 419/17708 | 0.800(0.704-0.909) | 0.001 | 0.852(0.737-0.985) | 0.030 |
| Hip replacement | 172/7617 | 0.794(0.673-0.936) | 0.006 | 0.754(0.617-0.920) | 0.006 |
| Knee replacement | 219/8672 | 0.788(0.660-0.941) | 0.009 | 0.851(0.701-1.033) | 0.102 |
| **AD** | | | | | |
| **Non-OA** | 1862/400911 |  |  |  |  |
| **OA** | 576/62360 | **1.098(0.986-1.224)** | **0.090** | **1.120(1.001-1.252)** | **0.047** |
| **Site of OA** |  |  |  |  |  |
| Hand OA | 47/4041 | **1.248(0.840-1.854)** | **0.273** | 1.154(0.746-1.785) | 0.520 |
| Hip OA | 77/6751 | 1.118(0.850-1.471) | 0.426 | 1.253(0.947-1.659) | 0.114 |
| Knee OA | 169/13961 | **1.419(1.174-1.713)** | **<0.001** | **1.396(1.129-1.727)** | **0.002** |
| **Drug** |  |  |  |  |  |
| **Nonuse ^c^** | 320/33661 |  |  |  |  |
| **Drug** | 120/17640 | **0.638(0.517-0.788)** | **<0.001** | **0.640(0.518-0.790)** | **<0.001** |
| **Drug_kind** |  |  |  |  |  |
| NSAID | 78/11334 | **0.643(0.502-0.824)** | **<0.001** | **0.646(0.504-0.828)** | **0.001** |
| Opioid | 84/12295 | **0.541(0.377-0.776)** | **0.001** | **0.533(0.372-0.764)** | **0.001** |
| Glucosamine | 9/1267 | 0.561(0.286-1.099) | 0.092 | 0.553(0.281-1.087) | 0.086 |
| Steroids injected | 39/4727 | 0.761(0.545-1.062) | 0.109 | 0.732(0.522-1.026) | 0.070 |
| **Surgery** |  |  |  |  |  |
| **Non-surgery** | 44518/381 |  |  |  |  |
| **OA surgery** | 176/17465 | **0.768(0.629-0.938)** | **0.010** | **0.791(0.645-0.969)** | **0.024** |
| Hip replacement | 70/7515 | 0.791(0.613-1.021) | 0.072 | 0.791(0.605-1.034) | 0.086 |
| Knee replacement | 95/8548 | **0.729(0.549-0.968)** | **0.029** | 0.770(0.579-1.025) | 0.073 |
| **VD** | | | | | |
| **Non-OA** | 998/400047 |  |  |  |  |
| **OA** | 314/62098 | **1.222(1.052-1.418)** | **0.009** | **1.092(0.937-1.272)** | **0.259** |
| **Site of OA** |  |  |  |  |  |
| Hand OA | 24/4018 | **1.334(0.803-2.214)** | **0.265** | 1.067(0.629-1.810) | 0.811 |
| Hip OA | 46/6720 | 1.415(0.985-2.032) | 0.061 | 1.381(0.968-1.971) | 0.075 |
| Knee OA | 81/13873 | 1.415(1.091-1.836) | 0.009 | 1.118(0.837-1.494) | 0.450 |
| **Drug** |  |  |  |  |  |
| **Nonuse ^c^** | 154/33495 |  |  |  |  |
| **Drug** | 81/17601 | 0.885(0.677-1.158) | 0.374 | 0.888(0.679-1.162) | 0.386 |
| **Drug_kind** |  |  |  |  |  |
| NSAID | 48/11304 | 0.816(0.591-1.128) | 0.218 | 0.822(0.595-1.135) | 0.233 |
| Opioid | 55/12266 | **0.574(0.353-0.936)** | **0.026** | **0.577(0.354-0.941)** | **0.028** |
| Glucosamine | 11/1269 | 1.510(0.816-2.792) | 0.189 | 1.417(0.770-2.606) | 0.263 |
| Steroids injected | 27/4715 | 1.090(0.723-1.642) | 0.682 | 1.047(0.692-1.585) | 0.827 |
| **Surgery** |  |  |  |  |  |
| **Non-surgery** | 201/44338 |  |  |  |  |
| **OA surgery** | 105/17394 | 0.870(0.666-1.136) | 0.305 | 0.838(0.640-1.099) | 0.201 |
| Hip replacement | 43/7488 | 0.959(0.691-1.333) | 0.805 | 0.899(0.628-1.286) | 0.559 |
| Knee replacement | 58/8511 | 0.804(0.548-1.182) | 0.267 | 0.815(0.557-1.192) | 0.291 |

The results are derived from Cox proportional hazard regression models in two models. Bold indicates statistical significance (P value<0.05).

Model 2 was additionally controlled for APOE ε4, ethnicity, education, TPA, BMI, smoking and alcohol than model 1.

Abbreviation: OA, osteoarthritis; ACD, all-cause dementia; AD, Alzheimer’s disease; VD, vascular dementia; APOE, Apolipoprotein E; BMI, body mass index; TPA, total physical activity; HR, Hazard ratio; CI, Confidence interval; NSAID, nonsteroidal anti-inflammatory drugs.

### Part II Other characteristics with OA

#### Supplementary Table 14. The association of the characteristics of OA patients as exposures with subsequent dementia outcome.

|  | **Number of incident dementia/ Number of participants** | **Model 1**  **(Gender+ Age)** | | **Model 2**  **(+APOE ε4+Ethnicity+Education+TPA**  **+BMI+Smoking+Alcohol)** | | **Matched**  **(1:4)** | |
| --- | --- | --- | --- | --- | --- | --- | --- |
|  |  | **HR (95%CI)** | **P value** | **HR (95%CI)** | **P value** | **HR (95%CI)** | **P value** |
| **Pain experience** |  |  |  |  |  |  |  |
| No | 1258/3783 |  |  |  |  |  |  |
| Yes | 39/13026 | 1.198(0.578-2.480) | 0.627 | 1.360(0.562-3.294) | 0.496 |  |  |
| **Duration** |  |  |  |  |  |  |  |
| 3-12 months | 3/1941 |  |  |  |  |  |  |
| 1-5 years | 16/3928 | 2.545(0.742-8.737) | 0.138 | 2.018(0.574-7.095) | 0.274 |  |  |
| ≥5 years | 20/7104 | 1.952(0.578-6.591) | 0.281 | 1.508(0.436-5.213) | 0.517 |  |  |
| **Joint injury history** |  | | | | | | |
| No | 1282/62715 |  |  |  |  |  |  |
| Yes | 15/366 | **2.149(1.291-3.576)** | **0.003** | **1.985(1.064-3.703)** | **0.031** | 1.733(0.823-3.649) | 0.148 |
| **Depression** |  | | | | | | |
| No | 945/44002 |  |  |  |  |  |  |
| Yes | 352/15673 | **1.486(1.311-1.685)** | **<0.001** | **1.382(1.196-1.596)** | **<0.001** |  |  |
| **Obesity** |  | | | | | | |
| No | 780/39510 |  |  |  |  |  |  |
| Yes | 497/23134 | 1.090(0.974-1.220) | 0.134 | 1.074(0.878-1.313) | 0.490 |  |  |
| **Comorbid state of RA** |  |  |  |  |  |  |  |
| No | 1242/60982 |  |  |  |  |  |  |
| Yes | 55/2099 | 1.207(0.921-1.581) | 0.173 | 1.195(0.874-1.634) | 0.266 | 1.236(0.870-1.756) | 0.236 |
| **Work involving heavy manual/ physical work** |  | | | | | | |
| Rarely/ NA | 86/13429 |  |  |  |  |  |  |
| Sometimes | 46/5762 | 1.196(0.834-1.713) | 0.331 | 1.248(0.817-1.907) | 0.305 |  |  |
| Usually | 8/1903 | 0.622(0.300-1.289) | 0.201 | 0.436(0.147-1.289) | 0.133 |  |  |
| Always | 15/1903 | 1.305(0.746-2.280) | 0.351 | 1.138(0.485-2.669) | 0.767 |  |  |
| **Work involving walk/standing** |  | | | | | | |
| Rarely/ NA | 36/6467 |  |  |  |  |  |  |
| Sometimes | 53/7097 | 1.289(0.843-1.972) | 0.241 | 1.365(0.856-2.177) | 0.191 |  |  |
| Usually | 36/3848 | 1.114(0.873-1.422) | 0.386 | 1.046(0.770-1.422) | 0.772 |  |  |
| Always | 36/5586 | 1.035(0.888-1.207) | 0.660 | 1.010(0.814-1.252) | 0.930 |  |  |

The results are derived from Cox proportional hazard regression models in two models. Large disparities between the observation and control groups for some items were matched using the propensity score matching. Bold indicates statistical significance (P value<0.05).

Model 2 was additionally controlled for APOE ε4, ethnicity, education, TPA, BMI, smoking and alcohol than model 1.

Abbreviation: OA, osteoarthritis; RA, rheumatoid arthritis; APOE, Apolipoprotein E; BMI, body mass index; TPA, total physical activity; HR, Hazard ratio; CI, Confidence interval.

### Part III Therapy in drugs

#### Supplementary Table 15. The association of osteoarthritis and usage of drugs with subsequent dementia outcome in participants.

| **Exposure** |  | **Model 1**  **(Gender+Age)** | | **Model 2**  **(+APOE ε4 +Ethnicity+Education +TPA+BMI+Smoking+Alcohol)** | |  | |
| --- | --- | --- | --- | --- | --- | --- | --- |
|  | **Number of incident dementia/ Number of participants** | **HR (95%CI)** | **P value** | **HR (95%CI)** | **P value** |  |  |
| **ACD** | | | | | |  |  |
| **Exposure elements** |  |  |  |  |  |  |  |
| Non_OA ^a^ | 3370/324242 |  |  |  |  |  |  |
| OA | 1028/51889 | **1.122(1.046-1.204)** | **0.001** | **1.116(1.039-1.199)** | **0.003** |  |  |
| Nonuse | 692/34033 | **1.133(1.045-1.228)** | **0.002** | **1.193(1.098-1.297)** | **<0.001** |  |  |
| Drug | 336/17856 | 0.935(0.836-1.045) | 0.233 | 0.991(0.885-1.110) | 0.878 |  |  |
| Nonuse ^b^ | 692/34033 |  |  |  |  |  |  |
| Drug | 336/17856 | **0.824(0.723-0.938)** | **0.004** | **0.826(0.725-0.941)** | **0.004** |  |  |
| **Lag time** |  |  |  |  |  |  |  |
| Excluding last two years | 303/17823 | **0.744(0.650-0.851)** | **<0.001** | **0.746(0.652-0.854)** | **<0.001** |  |  |
| **Adm.R** |  |  |  |  |  |  |  |
| Oral | 273/15984 | **0.745(0.647-0.857)** | **<0.001** | **0.747(0.649-0.859)** | **<0.001** |  |  |
| Topical | 176/8470 | **0.838(0.710-0.989)** | **0.037** | **0.834(0.707-0.984)** | **0.032** |  |  |
| **Drug_kind** |  |  |  |  |  |  |  |
| NSAID | 189/11445 | **0.717(0.610-0.842)** | **<0.001** | **0.719(0.612-0.845)** | **<0.001** |  |  |
| Opioid | 220/12431 | **0.746(0.641-0.868)** | **<0.001** | **0.747(0.642-0.870)** | **<0.001** |  |  |
| Glucosamine | 33/1291 | 1.004(0.708-1.424) | 0.982 | 1.005(0.708-1.425) | 0.978 |  |  |
| Steroid | 104/4792 | 0.922(0.751-1.134) | 0.443 | 0.911(0.741-1.120) | 0.376 |  |  |
| **AD** | | | | | |  |  |
| **Exposure elements** |  |  |  |  |  |  |  |
| Non_OA ^a^ | 1489/322361 |  |  |  |  |  |  |
| OA | 456/51317 | **1.089(0.980-1.210)** | **0.114** | **1.127(1.013-1.254)** | **0.028** |  |  |
| Nonuse | 320/33661 | **1.170(1.038-1.318)** | **0.010** | **1.256(1.111-1.420)** | **<0.001** |  |  |
| Drug | 136/17656 | 0.842(0.707-1.003) | 0.054 | 0.914(0.765-1.091) | 0.319 |  |  |
| Nonuse ^b^ | 320/33661 |  |  |  |  |  |  |
| Drug | 136/17656 | **0.721(0.590-0.881)** | **0.001** | **0.723(0.591-0.883)** | **0.002** |  |  |
| **Lag time** |  |  |  |  |  |  |  |
| Excluding last two years | 120/17640 | **0.636(0.516-0.785)** | **<0.001** | **0.638(0.517-0.787)** | **<0.001** |  |  |
| **Adm.R** |  |  |  |  |  |  |  |
| Oral | 106/15817 | **0.625(0.502-0.779)** | **<0.001** | **0.626(0.502-0.780)** | **<0.001** |  |  |
| Topical | 70/8364 | **0.715(0.552-0.926)** | **0.011** | **0.717(0.553-0.929)** | **0.012** |  |  |
| **Drug_kind** |  |  |  |  |  |  |  |
| NSAID | 78/11334 | **0.638(0.498-0.818)** | **<0.001** | **0.642(0.501-0.822)** | **<0.001** |  |  |
| Opioid | 84/12295 | **0.614(0.483-0.781)** | **<0.001** | **0.616(0.484-0.783)** | **<0.001** |  |  |
| Glucosamine | 9/1267 | 0.590(0.304-1.145) | 0.119 | 0.598(0.308-1.161) | 0.129 |  |  |
| Steroid | 39/4727 | 0.745(0.534-1.039) | 0.083 | 0.735(0.527-1.026) | 0.071 |  |  |
| **VD** | | | | | |  |  |
| **Exposure elements** |  |  |  |  |  |  |  |
| Non_OA ^a^ | 760/321632 |  |  |  |  |  |  |
| OA | 246/51107 | **1.203(1.040-1.390)** | **0.013** | 1.115(0.962-1.291) | 0.148 |  |  |
| Nonuse | 154/33495 | 1.106(0.933-1.312) | 0.247 | 1.114(0.934-1.329) | 0.229 |  |  |
| Drug | 92/17612 | 1.117(0.902-1.385) | 0.311 | 1.144(0.919-1.425) | 0.229 |  |  |
| Nonuse ^b^ | 154/33495 |  |  |  |  |  |  |
| Drug | 92/17612 | 1.001(0.773-1.296) | 0.992 | 1.005(0.776-1.301) | 0.970 |  |  |
| **Lag time** |  |  |  |  |  |  |  |
| Excluding last two years | 81/17601 | 0.883(0.675-1.156) | 0.364 | 0.886(0.677-1.160) | 0.380 |  |  |
| **Adm.R** |  |  |  |  |  |  |  |
| Oral | 72/15783 | 0.873(0.660-1.155) | 0.342 | 0.877(0.663-1.161) | 0.359 |  |  |
| Topical | 44/8338 | 0.933(0.667-1.305) | 0.686 | 0.910(0.650-1.273) | 0.582 |  |  |
| **Drug_kind** |  |  |  |  |  |  |  |
| NSAID | 48/11304 | 0.812(0.587-1.123) | 0.208 | 0.820(0.593-1.134) | 0.231 |  |  |
| Opioid | 55/12266 | 0.831(0.611-1.130) | 0.238 | 0.830(0.609-1.129) | 0.234 |  |  |
| Glucosamine | 11/1269 | 1.492(0.809-2.752) | 0.200 | 1.470(0.797-2.711) | 0.218 |  |  |
| Steroid | 27/4715 | 1.061(0.705-1.597) | 0.776 | 1.016(0.674-1.531) | 0.940 |  |  |

The results are derived from Cox proportional hazard regression models in two models. Bold indicates statistical significance (P value<0.05).

Model 2 was additionally controlled for APOE ε4, ethnicity, education, TPA, BMI, smoking and alcohol than model 1.

a, the model was based on total group, b, the model was based on OA participants.

Abbreviation: OA, osteoarthritis; ACD, all-cause dementia; AD, Alzheimer’s disease; VD, vascular dementia; APOE, Apolipoprotein E; BMI, body mass index; TPA, total physical activity; HR, Hazard ratio; CI, Confidence interval; NSAID, nonsteroidal anti-inflammatory drugs.

#### Supplementary Table 16. The association of the NSAID therapy with subsequent dementia outcome in participants.

| **Exposure** |  | **Model 1**  **(Gender+Age)** | | **Model 2**  **(+APOE ε4 +Ethnicity+Education +TPA+BMI+Smoking+Alcohol)** | |  | |
| --- | --- | --- | --- | --- | --- | --- | --- |
|  | **Number of incident dementia/ Number of participants** | **HR (95%CI)** | **P value** | **HR (95%CI)** | **P value** |  |  |
| **ACD** | | | | | |  |  |
| **Exposure elements** |  |  |  |  |  |  |  |
| Non-OA ^a^ | 3370/324242 |  |  |  |  |  |  |
| OA | 1028/51889 | **1.122(1.046-1.204)** | **0.001** | **1.116(1.039-1.199)** | **0.003** |  |  |
| Nonuse | 819/40426 | **1.110(1.030-1.197)** | **0.006** | **1.167(1.080-1.262)** | **<0.001** |  |  |
| NSAID | 209/11463 | 0.899(0.782-1.033) | 0.133 | 0.953(0.828-1.097) | 0.501 |  |  |
| Nonuse ^b^ | 819/40426 |  |  |  |  |  |  |
| NSAID | 209/11463 | **0.809(0.695-0.942)** | **0.006** | **0.811(0.697-0.944)** | **0.007** |  |  |
| **Lag time** |  |  |  |  |  |  |  |
| Excluding last two years | 197/11451 | **0.763(0.653-0.892)** | **0.001** | **0.765(0.655-0.894)** | **0.001** |  |  |
| **Adm.R** |  |  |  |  |  |  |  |
| Oral | 166/9838 | **0.772(0.653-0.912)** | **0.002** | **0.776(0.657-0.917)** | **0.003** |  |  |
| Topical | 96/4597 | **0.793(0.642-0.980)** | **0.032** | **0.784(0.634-0.968)** | **0.024** |  |  |
| **Cumulative exposure time** |  |  |  |  |  |  |  |
| 0-60 | 133/7087 |  |  |  |  |  |  |
| 61-180 | 57/4082 | 0.880(0.644-1.201) | 0.420 | 0.858(0.609-1.209) | 0.382 |  |  |
| 181-540 | 2/104 | 1.093(0.270-4.416) | 0.901 | 1.264(0.311-5.130) | 0.743 |  |  |
| ≥541 | 2/164 | 0.517(0.128-2.093) | 0.355 | 0.610(0.150-2.480) | 0.490 |  |  |
| **CHM-STRUC** |  |  |  |  |  |  |  |
| Acetic acid | 78/4386 | 0.845(0.670-1.066) | 0.156 | 0.851(0.674-1.073) | 0.173 |  |  |
| Propionic acid | 104/6649 | **0.728(0.594-0.893)** | **0.002** | **0.735(0.600-0.902)** | **0.003** |  |  |
| Coxibs | 24/806 | 1.207(0.804-1.812) | 0.364 | 1.190(0.793-1.786) | 0.402 |  |  |
| Oxicams | 7/424 | 0.676(0.321-1.422) | 0.302 | 0.665(0.316-1.399) | 0.282 |  |  |
| **Substances** |  |  |  |  |  |  |  |
| Diclofenac | 73/4097 | 0.864(0.680-1.097) | 0.230 | 0.873(0.687-1.110) | 0.267 |  |  |
| Ibuprofen | 60/3692 | **0.697(0.536-0.906)** | **0.007** | **0.696(0.536-0.905)** | **0.007** |  |  |
| Naproxen | 50/3592 | **0.709(0.533-0.943)** | **0.018** | **0.724(0.544-0.964)** | **0.027** |  |  |
| Celecoxib | 7/388 | 0.691(0.328-1.453) | 0.329 | 0.677(0.322-1.426) | 0.305 |  |  |
| Etodolac | 3/142 | 0.774(0.249-2.406) | 0.658 | 0.729(0.234-2.267) | 0.585 |  |  |
| Etoricoxib | 7/212 | 1.419(0.675-2.987) | 0.356 | 1.353(0.643-2.847) | 0.427 |  |  |
| Meloxicam | 5/326 | 0.646(0.268-1.556) | 0.330 | 0.642(0.266-1.547) | 0.323 |  |  |
| Mefenamic | 1/120 | 0.621(0.087-4.418) | 0.634 | 0.731(0.103-5.203) | 0.754 |  |  |
| Indometacin | 3/202 | 0.618(0.199-1.921) | 0.406 | 0.619(0.199-1.925) | 0.407 |  |  |
| Rofecoxib | 8/224 | 1.408(0.702-2.825) | 0.336 | 1.414(0.705-2.838) | 0.329 |  |  |
| **AD** | | | | | |  |  |
| **Exposure elements** |  |  |  |  |  |  |  |
| Non-OA ^a^ | 1489/322361 |  |  |  |  |  |  |
| OA | 456/51317 | 1.089(0.980-1.210) | 0.114 | **1.127(1.013-1.254)** | **0.028** |  |  |
| Nonuse | 367/39974 | 1.111(0.993-1.244) | 0.067 | **1.191(1.061-1.338)** | **0.003** |  |  |
| NSAID | 89/11343 | 0.850(0.687-1.052) | 0.134 | 0.923(0.745-1.145) | 0.468 |  |  |
| Nonuse ^b^ | 367/39974 |  |  |  |  |  |  |
| NSAID | 89/11343 | **0.767(0.609-0.967)** | **0.025** | **0.771(0.611-0.972)** | **0.028** |  |  |
| **Lag time** |  |  |  |  |  |  |  |
| Excluding last two years | 82/11336 | **0.707(0.557-0.898)** | **0.005** | **0.710(0.559-0.903)** | **0.005** |  |  |
| **Adm.R** |  |  |  |  |  |  |  |
| Oral | 66/9738 | **0.686(0.528-0.891)** | **0.005** | **0.689(0.530-0.896)** | **0.005** |  |  |
| Topical | 43/4544 | 0.777(0.567-1.067) | 0.119 | 0.778(0.567-1.067) | 0.119 |  |  |
| **Cumulative exposure time** |  |  |  |  |  |  |  |
| 0-60 | 57/7011 |  |  |  |  |  |  |
| 61-180 | 22/4047 | 0.777(0.474-1.273) | 0.317 | 0.823(0.474-1.428) | 0.488 |  |  |
| 181-540 | 2/104 | 2.444(0.596-10.019) | 0.214 | 3.045(0.733-12.647) | 0.125 |  |  |
| ≥541 | 2/164 | 1.288(0.314-5.289) | 0.726 | 1.568(0.374-6.569) | 0.538 |  |  |
| **CHM-STRUC** |  |  |  |  |  |  |  |
| Acetic acid | 30/4338 | 0.731(0.504-1.060) | 0.099 | 0.743(0.512-1.078) | 0.118 |  |  |
| Propionic acid | 41/6586 | **0.642(0.465-0.887)** | **0.007** | **0.641(0.464-0.885)** | **0.007** |  |  |
| Coxibs | 9/791 | 1.009(0.521-1.954) | 0.980 | 0.986(0.509-1.910) | 0.966 |  |  |
| Oxicams | 1/418 | 0.213(0.030-1.516) | 0.122 | 0.217(0.030-1.544) | 0.127 |  |  |
| **Substances** |  |  |  |  |  |  |  |
| Diclofenac | 27/4051 | 0.718(0.486-1.062) | 0.097 | 0.733(0.496-1.084) | 0.119 |  |  |
| Ibuprofen | 23/3655 | **0.591(0.388-0.901)** | **0.015** | **0.583(0.382-0.888)** | **0.012** |  |  |
| Naproxen | 22/3564 | 0.708(0.460-1.088) | 0.115 | 0.708(0.460-1.089) | 0.116 |  |  |
| Celecoxib | 4/385 | 0.866(0.323-2.320) | 0.775 | 0.870(0.324-2.332) | 0.782 |  |  |
| Etodolac | 3/142 | 1.707(0.548-5.319) | 0.357 | 1.621(0.519-5.059) | 0.406 |  |  |
| Etoricoxib | 2/207 | 0.911(0.227-3.657) | 0.895 | 0.786(0.196-3.159) | 0.735 |  |  |
| Meloxicam | 1/322 | 0.286(0.040-2.038) | 0.212 | 0.301(0.042-2.145) | 0.231 |  |  |
| Mefenamic | 1/120 | 1.365(0.191-9.733) | 0.756 | 1.721(0.241-12.285) | 0.588 |  |  |
| Indometacin | 1/200 | 0.469(0.066-3.340) | 0.450 | 0.494(0.069-3.523) | 0.482 |  |  |
| Rofecoxib | 3/219 | 1.187(0.381-3.699) | 0.767 | 1.218(0.391-3.795) | 0.734 |  |  |
| **VD** | | | | | |  |  |
| **Exposure elements** |  |  |  |  |  |  |  |
| Non-OA ^a^ | 760/321632 |  |  |  |  |  |  |
| OA | 246/51107 | **1.203(1.040-1.390)** | **0.013** | 1.115(0.962-1.291) | 0.148 |  |  |
| Nonuse | 193/39800 | 1.143(0.978-1.334) | 0.092 | 1.147(0.976-1.348) | 0.095 |  |  |
| NSAID | 53/11307 | 0.996(0.755-1.313) | 0.975 | 1.020(0.770-1.350) | 0.892 |  |  |
| Nonuse ^b^ | 193/39800 |  |  |  |  |  |  |
| NSAID | 53/11307 | 0.867(0.640-1.175) | 0.358 | 0.874(0.645-1.186) | 0.388 |  |  |
| **Lag time** |  |  |  |  |  |  |  |
| Excluding last two years | 52/11306 | 0.851(0.627-1.156) | 0.302 | 0.858(0.632-1.166) | 0.329 |  |  |
| **Adm.R** |  |  |  |  |  |  |  |
| Oral | 43/9715 | 0.845(0.607-1.175) | 0.317 | 0.857(0.616-1.193) | 0.362 |  |  |
| Topical | 24/4525 | 0.837(0.547-1.279) | 0.410 | 0.814(0.532-1.245) | 0.343 |  |  |
| **Cumulative exposure time** |  |  |  |  |  |  |  |
| 0-60 | 33/6987 |  |  |  |  |  |  |
| 61-180 | 15/4040 | 0.922(0.500-1.702) | 0.796 | 0.995(0.510-1.943) | 0.988 |  |  |
| 181-540 | 1/103 | 2.182(0.298-15.976) | 0.443 | 2.540(0.343-18.799) | 0.362 |  |  |
| ≥541 | 0/162 | - | - | - | - |  |  |
| **CHM-STRUC** |  |  |  |  |  |  |  |
| Acetic acid | 19/4327 | 0.869(0.542-1.391) | 0.559 | 0.870(0.543-1.395) | 0.564 |  |  |
| Propionic acid | 30/6575 | 0.888(0.604-1.304) | 0.544 | 0.914(0.622-1.343) | 0.647 |  |  |
| Coxibs | 5/787 | 1.095(0.450-2.660) | 0.842 | 1.084(0.446-2.635) | 0.859 |  |  |
| Oxicams | 4/421 | 1.664(0.618-4.480) | 0.314 | 1.554(0.576-4.191) | 0.384 |  |  |
| **Substances** |  |  |  |  |  |  |  |
| Diclofenac | 18/4042 | 0.900(0.555-1.458) | 0.668 | 0.909(0.560-1.473) | 0.698 |  |  |
| Ibuprofen | 19/3651 | 0.929(0.580-1.489) | 0.760 | 0.945(0.590-1.515) | 0.814 |  |  |
| Naproxen | 11/3598 | 0.667(0.363-1.225) | 0.192 | 0.706(0.384-1.297) | 0.262 |  |  |
| Celecoxib | 0/381 |  |  |  |  |  |  |
| Etodolac | 0/139 |  |  |  |  |  |  |
| Etoricox7 | 1/206 | 0.884(0.124-6.307) | 0.902 | 0.876(0.123-6.254) | 0.895 |  |  |
| Meloxicam | 2/323 | 1.124(0.279-4.529) | 0..869 | 1.046(0.259-4.218) | 0.950 |  |  |
| Mefenamic | 0/119 |  |  |  |  |  |  |
| Indometacin | 1/200 | 0.800(0.112-5.715) | 0.824 | 0.762(0.107-5.445) | 0.786 |  |  |
| Rofecoxib | 3/219 | 2.232(0.713-6.981) | 0.168 | 2.332(0.746-7.300) | 0.145 |  |  |

The results are derived from Cox proportional hazard regression models in two models. Bold indicates statistical significance (P value<0.05).

Model 2 was additionally controlled for APOE ε4, ethnicity, education, TPA, BMI, smoking and alcohol than model 1.

a, the model was based on total group, b, the model was based on OA participants.

Abbreviation: OA, osteoarthritis; ACD, all-cause dementia; AD, Alzheimer’s disease; VD, vascular dementia; APOE, Apolipoprotein E; BMI, body mass index; TPA, total physical activity; HR, Hazard ratio; CI, Confidence interval; NSAID, nonsteroidal anti-inflammatory drugs; Adm.R, route of administration; CHM-STRUC, chemical structure

#### Supplementary Table 17. The association of the opioid therapy with subsequent dementia outcome in participants.

| **Exposure** |  | **Model 1**  **(Gender+Age)** | | **Model 2**  **(+APOE ε4 +Ethnicity+Education +TPA+BMI+Smoking+Alcohol)** | |  | |
| --- | --- | --- | --- | --- | --- | --- | --- |
|  | **Number of incident dementia/ Number of participants** | **HR (95%CI)** | **P value** | **HR (95%CI)** | **P value** |  |  |
| **ACD** | | | | | |  |  |
| **Exposure elements** |  |  |  |  |  |  |  |
| Non-OA ^a^ | 3370/324242 |  |  |  |  |  |  |
| OA | 1028/51889 | **1.122(1.046-1.204)** | **0.001** | **1.116(1.039-1.199)** | **0.003** |  |  |
| Nonuse | 783/39433 | **1.103(1.022-1.191)** | **0.012** | **1.161(1.072-1.256)** | **<0.001** |  |  |
| Opioid | 245/12456 | 0.941(0.827-1.070) | 0.354 | 0.998(0.876-1.138) | 0.980 |  |  |
| Nonuse ^b^ | 783/39433 |  |  |  |  |  |  |
| Opioid | 245/12456 | **0.853(0.739-0.985)** | **0.030** | **0.853(0.739-0.985)** | **0.030** |  |  |
| **Lag time** |  |  |  |  |  |  |  |
| Excluding last two years | 229/12440 | **0.798(0.688-0.924)** | **0.003** | **0.798(0.688-0.924)** | **0.003** |  |  |
| **Adm.R** |  |  |  |  |  |  |  |
| Oral | 228/12393 | **0.797(0.688-0.924)** | **0.003** | **0.797(0.688-0.924)** | **0.003** |  |  |
| Topical | 13/421 | 1.318(0.762-2.281) | 0.323 | 1.268(0.732-2.195) | 0.397 |  |  |
| **Cumulative exposure time** |  |  |  |  |  |  |  |
| 0-10 | 231/12470 |  |  |  |  |  |  |
| 11-30 | 49/2169 | 1.175(0.863-1.600) | 0.304 | 1.287(0.921-1.797) | 0.139 |  |  |
| 31-90 | 1/30 | 1.519(0.213-10.835) | 0.677 | 1.650(0.229-11.874) | 0.619 |  |  |
| ≥91 | 0/2 | - | - | - | - |  |  |
| **CHM-STRUC** |  |  |  |  |  |  |  |
| Opium Alkaloids | 216/11579 | **0.803(0.690-0.933)** | **0.004** | **0.803(0.689-0.932)** | **0.004** |  |  |
| Other | 45/2793 | **0.704(0.522-0.951)** | **0.022** | **0.694(0.514-0.937)** | **0.017** |  |  |
| **Substances** |  |  |  |  |  |  |  |
| Codeine | 213/11438 | **0.802(0.689-0.933)** | **0.004** | **0.801(0.688-0.932)** | **0.004** |  |  |
| Tramadol | 45/2755 | **0.717(0.531-0.968)** | **0.030** | **0.707(0.523-0.954)** | **0.024** |  |  |
| Morphine | 8/230 | 1.685(0.840-3.383) | 0.142 | 1.620(0.806-3.254) | 0.176 |  |  |
| Oxycodone | 4/122 | 1.455(0.545-3.886) | 0.455 | 1.292(0.483-3.454) | 0.610 |  |  |
| **AD** | | | | | |  |  |
| **Exposure elements** |  |  |  |  |  |  |  |
| Non-OA ^a^ | 1489/322361 |  |  |  |  |  |  |
| OA | 456/51317 | **1.089(0.980-1.210)** | **0.114** | **1.127(1.012-1.254)** | **0.028** |  |  |
| Nonuse | 360/39010 | **1.133(1.012-1.270)** | **0.031** | **1.213(1.079-1.363)** | **0.001** |  |  |
| Opioid | 96/12307 | 0.819(0.667-1.006) | 0.057 | 0.893(0.725-1.099) | 0.285 |  |  |
| Nonuse ^b^ | 360/39010 |  |  |  |  |  |  |
| Opioid | 96/12307 | **0.725(0.579-0.908)** | **0.005** | **0.727(0.580-0.911)** | **0.006** |  |  |
| **Lag time** |  |  |  |  |  |  |  |
| Excluding last two years | 87/12298 | **0.657(0.520-0.830)** | **<0.001** | **0.659(0.521-0.833)** | **<0.001** |  |  |
| **Adm.R** |  |  |  |  |  |  |  |
| Oral | 87//12252 | **0.659(0.522-0.833)** | **<0.001** | **0.661(0.523-0.836)** | **0.001** |  |  |
| Topical | 5/413 | 1.078(0.446-2.607) | 0.867 | 1.016(0.420-2.459) | 0.973 |  |  |
| **Cumulative exposure time** |  |  |  |  |  |  |  |
| 0-10 | 94/12333 |  |  |  |  |  |  |
| 11-30 | 18/2138 | 1.059(0.639-1.754) | 0.824 | 1.077(0.606-1.916) | 0.800 |  |  |
| 31-90 | 1/30 | **3.763(0.524-27.013)** | **0.188** | 4.714(0.638-34.804) | 0.129 |  |  |
| ≥91 | 0/2 |  |  |  |  |  |  |
| **CHM-STRUC** |  |  |  |  |  |  |  |
| Opium Alkaloids | 82/11445 | **0.660(0.520-0.839)** | **0.001** | **0.661(0.520-0.840)** | **0.001** |  |  |
| Other | 14/2762 | **0.475(0.279-0.810)** | **0.006** | **0.472(0.277-0.805)** | **0.006** |  |  |
| **Substances** |  |  |  |  |  |  |  |
| Codeine | 81/11306 | **0.661(0.519-0.841)** | **0.001** | **0.661(0.519-0.841)** | **0.001** |  |  |
| Tramadol | 14/2724 | **0.484(0.284-0.826)** | **0.008** | **0.482(0.282-0.822)** | **0.007** |  |  |
| Morphine | 3/225 | 1.428(0.458-4.451) | 0.539 | 1.542(0.494-4.815) | 0.456 |  |  |
| Oxycodone | 1/119 | 0.770(0.108-5.483) | 0.794 | 0.698(0.098-4.973) | 0.720 |  |  |
| **VD** | | | | | |  |  |
| **Exposure elements** |  |  |  |  |  |  |  |
| Non-OA ^a^ | 760/321632 |  |  |  |  |  |  |
| OA | 246/51107 | 1.203(1.040-1.390) | 0.013 | 1.115(0.962-1.291) | 0.148 |  |  |
| Nonuse | 182/38832 | 1.117(0.953-1.310) | 0.172 | 1.129(0.957-1.331) | 0.150 |  |  |
| Opioid | 64/12275 | 1.069(0.829-1.377) | 0.609 | 1.094(0.846-1.416) | 0.493 |  |  |
| Nonuse ^b^ | 182/38832 |  |  |  |  |  |  |
| Opioid | 64/12275 | 0.952(0.716-1.266) | 0.737 | 0.947(0.712-1.259) | 0;706 |  |  |
| **Lag time** |  |  |  |  |  |  |  |
| Excluding last two years | 59/12270 | 0.878(0.655-1.178) | 0.387 | 0.873(0.651-1.172) | 0.367 |  |  |
| **Adm.R** |  |  |  |  |  |  |  |
| Oral | 2/410 | 0.893(0.222-3.598) | 0.873 | 0.791(0.196-3.194) | 0.742 |  |  |
| Topical | 59/12224 | 0.882(0.657-1.183) | 0.401 | 0.876(0.653-1.176) | 0.379 |  |  |
| **Cumulative exposure time** |  |  |  |  |  |  |  |
| 0-10 | 59/12298 |  |  |  |  |  |  |
| 11-30 | 16/2136 | 1.504(0.865-2.615) | 0.148 | 1.554(0.836-2.889) | 0.163 |  |  |
| 31-90 | 0/29 |  |  |  |  |  |  |
| ≥91 | 0/2 |  |  |  |  |  |  |
| **CHM-STRUC** |  |  |  |  |  |  |  |
| Opium Alkaloids | 56/11419 | 0.889(0.659-1.200) | 0.443 | 0.882(0.653-1.190) | 0.410 |  |  |
| Other | 12/2760 | 0.802(0.447-1.438) | 0.458 | 0.765(0.426-1.373) | 0.369 |  |  |
| **Substances** |  |  |  |  |  |  |  |
| Codeine | 55/11280 | 0.885(0.655-1.197) | 0.429 | 0.880(0.651-1.190) | 0.407 |  |  |
| Tramadol | 12/2722 | 0.816(0.455-1.464) | 0.495 | 0.779(0.434-1.397) | 0.401 |  |  |
| Morphine | 3/225 | 2.767(0.884-8.663) | 0.081 | 2.227(0.709-7.000) | 0.171 |  |  |
| Oxycodone | 2/120 | 3.087(0.766-12.441) | 0.113 | 2.397(0.592-9.707) | 0.221 |  |  |

The results are derived from Cox proportional hazard regression models in two models. Bold indicates statistical significance (P value<0.05).

Model 2 was additionally controlled for APOE ε4, ethnicity, education, TPA, BMI, smoking and alcohol than model 1.

a, the model was based on total group, b, the model was based on OA participants.

Abbreviation: OA, osteoarthritis; ACD, all-cause dementia; AD, Alzheimer’s disease; VD, vascular dementia; APOE, Apolipoprotein E; BMI, body mass index; TPA, total physical activity; HR, Hazard ratio; CI, Confidence interval; Adm.R, route of administration; CHM-STRUC, chemical structure.

### Part IV Therapy in surgery

#### Supplementary Table 18. The association of osteoarthritis and joint replacement as exposures with subsequent dementia outcome.

|  | **Number of incident dementia/ Number of participants** | **Model 1**  **(Gender+Age)** | | **Model 2**  **(+APOE ε4 +Ethnicity+Education +TPA**  **+BMI+Smoking+Alcohol)** | |  |
| --- | --- | --- | --- | --- | --- | --- |
|  |  | **HR (95%CI)** | **P value** | **HR (95%CI)** | **P value** |  |
| **ACD** | | | | | |  |
| **Exposure elements** |  |  | | | |  |
| Non-OA^a^ | 4277/400961 |  | | | |  |
| OA | 1281/62707 | **1.108(1.040-1.180)** | **0.002** | **1.111(1.034-1.193)** | **0.004** |  |
| Non-surgery | 836/44973 | **1.136(1.054-1.224)** | **0.001** | **1.115(1.024-1.214)** | **0.012** |  |
| OA surgery | 445/17734 | 0.952(0.862-1.050) | 0.325 | 1.0003(0.895-1.118) | 0.996 |  |
| Non-surgery ^b^ | 836/44973 |  | | | |  |
| OA surgery | 445/17734 | **0.848(0.755-0.952)** | **0.005** | 0.885(0.777-1.009) | 0.067 |  |
| **Lag time** |  |  |  |  |  |  |
| Excluding last two years | 419/17708 | **0.798(0.709-0.899)** | **<0.001** | **0.841(0.736-0.960)** | **0.011** |  |
| **Surgery types** |  |  |  |  |  |  |
| Hip replacement | 172/7617 | **0.786(0.667-0.927)** | **0.004** | 0.850(0.709-1.020) | 0.080 |  |
| Knee replacement | 219/8672 | **0.825(0.710-0.958)** | **0.012** | **0.825(0.695-0.979)** | **0.028** |  |
| **AD** | | | | | |  |
| **Exposure elements** |  |  |  |  |  |  |
| Non-OA^a^ | 1845/398529 |  |  |  |  |  |
| OA | 566/61992 | 1.086(0.988-1.194) | 0.089 | 1.110(0.997-1.237) | 0.057 |  |
| Non-surgery | 381/44518 | **1.145(1.024-1.280)** | **0.017** | 1.128(0.993-1.280) | 0.064 |  |
| OA surgery | 185/17474 | 0.862(0.741-1.004) | 0.057 | 0.971(0.818-1.151) | 0.731 |  |
| Non-surgery ^b^ | 381/44518 |  |  |  |  |  |
| OA surgery | 185/17474 | **0.759(0.636-0.905)** | **0.002** | 0.831(0.681-1.014) | 0.069 |  |
| **Lag time** |  |  |  |  |  |  |
| Excluding last two years | 176/17465 | **0.723(0.604-0.865)** | **<0.001** | **0.795(0.649-0.973)** | **0.026** |  |
| **Surgery types** |  |  |  |  |  |  |
| Hip replacement | 70/7515 | **0.683(0.529-0.882)** | **0.004** | 0.766(0.578-1.015) | 0.063 |  |
| Knee replacement | 95/8548 | **0.772(0.616-0.967)** | **0.025** | 0.818(0.632-1.060) | 0.128 |  |
| **VD** | | | | | |  |
| **Exposure elements** |  |  |  |  |  |  |
| Non-OA^a^ | 984/397668 |  |  |  |  |  |
| OA | 310/61736 | **1.174(1.032-1.336)** | **0.015** | 1.112(0.959-1.289) | 0.160 |  |
| Non-surgery | 201/44338 | **1.214(1.042-1.415)** | **0.013** | 1.122(0.940-1.338) | 0.202 |  |
| OA surgery | 109/17398 | 1.003(0.821-1.225) | 0.976 | 0.983(0.784-1.232) | 0.881 |  |
| Non-surgery ^b^ | 201/44366 |  |  |  |  |  |
| OA surgery | 109/17371 | 0.856(0.677-1.083) | 0.194 | 0.864(0.663-1.127) | 0.280 |  |
| **Lag time** |  |  |  |  |  |  |
| Excluding last two years | 105/17394 | 0.825(0.651-1.047) | 0.114 | 0.823(0.629-1.077) | 0.156 |  |
| **Surgery types** |  |  |  |  |  |  |
| Hip replacement | 43/7488 | 0.812(0.583-1.130) | 0.216 | 0.813(0.557-1.187) | 0.284 |  |
| Knee replacement | 58/8511 | 0.894(0.666-1.200) | 0.456 | 0.843(0.605-1.174) | 0.313 |  |

The results are derived from Cox proportional hazard regression models in two models. Bold indicates statistical significance (P value<0.05).

Model 2 was additionally controlled for APOE ε4, ethnicity, education, TPA, BMI, smoking and alcohol than model 1.

a, the model was based on total group, b, the model was based on OA participants.

Abbreviation: OA, osteoarthritis; ACD, all-cause dementia; AD, Alzheimer’s disease; VD, vascular dementia; APOE, Apolipoprotein E; BMI, body mass index; TPA, total physical activity; HR, Hazard ratio; CI, Confidence interval.

# Brain structure

#### Supplementary Table 19. The association between OA with treatments and brain cortical areas in UK Biobank.

| **Brain area** | **β** | **P value** | **FDR-P value** |
| --- | --- | --- | --- |
| lh_bankssts | -0.054 | 0.002 | 0.011 |
| lh_caudalanteriorcingulate | -0.009 | 0.605 | 0.739 |
| lh_caudalmiddlefrontal | -0.048 | 0.005 | 0.022 |
| lh_cuneus | -0.007 | 0.675 | 0.782 |
| lh_entorhinal | 0.001 | 0.934 | 0.954 |
| lh_fusiform | -0.029 | 0.075 | 0.155 |
| lh_inferiorparietal | -0.058 | 0.001 | 0.005 |
| lh_inferiortemporal | -0.036 | 0.026 | 0.084 |
| lh_isthmuscingulate | -0.014 | 0.407 | 0.548 |
| lh_lateraloccipital | -0.012 | 0.452 | 0.585 |
| lh_lateralorbitofrontal | -0.034 | 0.037 | 0.101 |
| lh_lingual | 0.005 | 0.763 | 0.853 |
| lh_medialorbitofrontal | -0.017 | 0.294 | 0.404 |
| lh_middletemporal | -0.044 | 0.007 | 0.029 |
| lh_paracentral | -0.020 | 0.256 | 0.376 |
| lh_parahippocampal | -0.030 | 0.081 | 0.163 |
| lh_parsopercularis | -0.063 | 0.000 | 0.003 |
| lh_parsorbitalis | -0.001 | 0.966 | 0.966 |
| lh_parstriangularis | -0.023 | 0.174 | 0.294 |
| lh_pericalcarine | 0.006 | 0.747 | 0.850 |
| lh_postcentral | -0.071 | <0.001 | <0.001 |
| lh_posteriorcingulate | -0.027 | 0.110 | 0.201 |
| lh_precentral | -0.034 | 0.039 | 0.101 |
| lh_precuneus | -0.054 | 0.001 | 0.007 |
| lh_rostralanteriorcingulate | -0.021 | 0.209 | 0.320 |
| lh_rostralmiddlefrontal | -0.022 | 0.171 | 0.294 |
| lh_superiorfrontal | -0.031 | 0.050 | 0.111 |
| lh_superiorparietal | -0.029 | 0.086 | 0.163 |
| lh_superiortemporal | -0.034 | 0.034 | 0.101 |
| lh_supramarginal | -0.062 | <0.001 | 0.002 |
| lh_frontalpole | -0.005 | 0.792 | 0.871 |
| lh_transversetemporal | -0.037 | 0.035 | 0.101 |
| lh_insula | -0.036 | 0.027 | 0.084 |
| rh_bankssts | -0.021 | 0.239 | 0.358 |
| rh_caudalanteriorcingulate | -0.036 | 0.043 | 0.101 |
| rh_caudalmiddlefrontal | -0.046 | 0.008 | 0.029 |
| rh_cuneus | -0.004 | 0.810 | 0.876 |
| rh_entorhinal | 0.003 | 0.864 | 0.919 |
| rh_fusiform | -0.040 | 0.012 | 0.041 |
| rh_inferiorparietal | -0.011 | 0.497 | 0.631 |
| rh_inferiortemporal | -0.029 | 0.068 | 0.145 |
| rh_isthmuscingulate | -0.022 | 0.190 | 0.305 |
| rh_lateraloccipital | -0.008 | 0.629 | 0.755 |
| rh_lateralorbitofrontal | -0.018 | 0.270 | 0.387 |
| rh_lingual | -0.009 | 0.600 | 0.739 |
| rh_medialorbitofrontal | -0.028 | 0.087 | 0.163 |
| rh_middletemporal | -0.058 | <0.001 | 0.003 |
| rh_paracentral | -0.025 | 0.151 | 0.269 |
| rh_parahippocampal | -0.023 | 0.187 | 0.305 |
| rh_parsopercularis | -0.053 | 0.003 | 0.013 |
| rh_parsorbitalis | 0.001 | 0.939 | 0.954 |
| rh_parstriangularis | 0.002 | 0.925 | 0.954 |
| rh_pericalcarine | 0.008 | 0.657 | 0.774 |
| rh_postcentral | -0.077 | <0.001 | <0.001 |
| rh_posteriorcingulate | -0.047 | 0.006 | 0.025 |
| rh_precentral | -0.048 | 0.003 | 0.015 |
| rh_precuneus | -0.059 | <0.001 | 0.003 |
| rh_rostralanteriorcingulate | -0.022 | 0.200 | 0.315 |
| rh_rostralmiddlefrontal | -0.017 | 0.292 | 0.404 |
| rh_superiorfrontal | -0.033 | 0.041 | 0.101 |
| rh_superiorparietal | -0.033 | 0.050 | 0.111 |
| rh_superiortemporal | -0.051 | 0.002 | 0.011 |
| rh_supramarginal | -0.072 | <0.001 | <0.001 |
| rh_frontalpole | -0.034 | 0.042 | 0.101 |
| rh_transversetemporal | -0.062 | <0.001 | 0.003 |
| rh_insula | -0.012 | 0.442 | 0.584 |
| **Drug** | | | |
| lh_bankssts | 0.075 | 0.030 | 0.592 |
| lh_caudalanteriorcingulate | 0.029 | 0.413 | 0.681 |
| lh_caudalmiddlefrontal | 0.045 | 0.183 | 0.592 |
| lh_cuneus | -0.022 | 0.523 | 0.719 |
| lh_entorhinal | 0.059 | 0.093 | 0.592 |
| lh_fusiform | 0.007 | 0.836 | 0.907 |
| lh_inferiorparietal | 0.062 | 0.065 | 0.592 |
| lh_inferiortemporal | 0.048 | 0.133 | 0.592 |
| lh_isthmuscingulate | 0.055 | 0.091 | 0.592 |
| lh_lateraloccipital | 0.023 | 0.479 | 0.681 |
| lh_lateralorbitofrontal | 0.028 | 0.382 | 0.681 |
| lh_lingual | 0.025 | 0.457 | 0.681 |
| lh_medialorbitofrontal | 0.028 | 0.383 | 0.681 |
| lh_middletemporal | 0.054 | 0.090 | 0.592 |
| lh_paracentral | 0.000 | 0.990 | 0.990 |
| lh_parahippocampal | 0.036 | 0.294 | 0.664 |
| lh_parsopercularis | -0.020 | 0.561 | 0.741 |
| lh_parsorbitalis | 0.040 | 0.215 | 0.592 |
| lh_parstriangularis | 0.005 | 0.874 | 0.916 |
| lh_pericalcarine | -0.036 | 0.302 | 0.664 |
| lh_postcentral | 0.042 | 0.186 | 0.592 |
| lh_posteriorcingulate | 0.040 | 0.240 | 0.592 |
| lh_precentral | 0.046 | 0.148 | 0.592 |
| lh_precuneus | 0.008 | 0.814 | 0.907 |
| lh_rostralanteriorcingulate | 0.024 | 0.459 | 0.681 |
| lh_rostralmiddlefrontal | -0.019 | 0.541 | 0.729 |
| lh_superiorfrontal | 0.038 | 0.230 | 0.592 |
| lh_superiorparietal | -0.003 | 0.940 | 0.969 |
| lh_superiortemporal | 0.015 | 0.629 | 0.766 |
| lh_supramarginal | 0.072 | 0.026 | 0.592 |
| lh_frontalpole | -0.049 | 0.150 | 0.592 |
| lh_transversetemporal | 0.046 | 0.180 | 0.592 |
| lh_insula | 0.040 | 0.205 | 0.592 |
| rh_bankssts | 0.009 | 0.797 | 0.907 |
| rh_caudalanteriorcingulate | 0.045 | 0.193 | 0.592 |
| rh_caudalmiddlefrontal | 0.028 | 0.406 | 0.681 |
| rh_cuneus | -0.010 | 0.758 | 0.878 |
| rh_entorhinal | 0.027 | 0.425 | 0.681 |
| rh_fusiform | 0.037 | 0.237 | 0.592 |
| rh_inferiorparietal | 0.060 | 0.064 | 0.592 |
| rh_inferiortemporal | 0.030 | 0.347 | 0.681 |
| rh_isthmuscingulate | 0.015 | 0.639 | 0.766 |
| rh_lateraloccipital | 0.017 | 0.584 | 0.756 |
| rh_lateralorbitofrontal | 0.038 | 0.242 | 0.592 |
| rh_lingual | 0.026 | 0.456 | 0.681 |
| rh_medialorbitofrontal | -0.006 | 0.838 | 0.907 |
| rh_middletemporal | 0.042 | 0.185 | 0.592 |
| rh_paracentral | 0.031 | 0.358 | 0.681 |
| rh_parahippocampal | 0.063 | 0.058 | 0.592 |
| rh_parsopercularis | -0.040 | 0.242 | 0.592 |
| rh_parsorbitalis | 0.005 | 0.868 | 0.916 |
| rh_parstriangularis | 0.023 | 0.485 | 0.681 |
| rh_pericalcarine | 0.002 | 0.959 | 0.974 |
| rh_postcentral | 0.092 | 0.005 | 0.315 |
| rh_posteriorcingulate | 0.038 | 0.254 | 0.598 |
| rh_precentral | 0.030 | 0.343 | 0.681 |
| rh_precuneus | 0.013 | 0.676 | 0.797 |
| rh_rostralanteriorcingulate | 0.024 | 0.474 | 0.681 |
| rh_rostralmiddlefrontal | 0.029 | 0.374 | 0.681 |
| rh_superiorfrontal | 0.022 | 0.476 | 0.681 |
| rh_superiorparietal | -0.016 | 0.624 | 0.766 |
| rh_superiortemporal | 0.053 | 0.103 | 0.592 |
| rh_supramarginal | 0.030 | 0.369 | 0.681 |
| rh_frontalpole | -0.051 | 0.131 | 0.592 |
| rh_transversetemporal | 0.018 | 0.596 | 0.756 |
| rh_insula | 0.044 | 0.169 | 0.592 |
| **Surgery** | | | |
| lh_bankssts | 0.152 | 0.014 | 0.381 |
| lh_caudalanteriorcingulate | -0.142 | 0.025 | 0.381 |
| lh_caudalmiddlefrontal | -0.172 | 0.005 | 0.317 |
| lh_cuneus | -0.017 | 0.776 | 0.927 |
| lh_entorhinal | 0.013 | 0.838 | 0.927 |
| lh_fusiform | -0.044 | 0.444 | 0.858 |
| lh_inferiorparietal | -0.017 | 0.785 | 0.927 |
| lh_inferiortemporal | -0.098 | 0.088 | 0.529 |
| lh_isthmuscingulate | -0.027 | 0.646 | 0.927 |
| lh_lateraloccipital | -0.010 | 0.857 | 0.927 |
| lh_lateralorbitofrontal | 0.026 | 0.651 | 0.927 |
| lh_lingual | -0.022 | 0.717 | 0.927 |
| lh_medialorbitofrontal | 0.000 | 0.997 | 0.997 |
| lh_middletemporal | -0.029 | 0.613 | 0.927 |
| lh_paracentral | -0.112 | 0.071 | 0.475 |
| lh_parahippocampal | -0.047 | 0.438 | 0.858 |
| lh_parsopercularis | -0.051 | 0.409 | 0.854 |
| lh_parsorbitalis | -0.063 | 0.284 | 0.749 |
| lh_parstriangularis | -0.093 | 0.126 | 0.639 |
| lh_pericalcarine | -0.053 | 0.406 | 0.854 |
| lh_postcentral | -0.060 | 0.295 | 0.749 |
| lh_posteriorcingulate | -0.079 | 0.192 | 0.669 |
| lh_precentral | -0.121 | 0.035 | 0.381 |
| lh_precuneus | -0.071 | 0.222 | 0.669 |
| lh_rostralanteriorcingulate | -0.054 | 0.359 | 0.845 |
| lh_rostralmiddlefrontal | -0.050 | 0.384 | 0.854 |
| lh_superiorfrontal | -0.070 | 0.212 | 0.669 |
| lh_superiorparietal | -0.081 | 0.183 | 0.669 |
| lh_superiortemporal | -0.011 | 0.853 | 0.927 |
| lh_supramarginal | 0.012 | 0.843 | 0.927 |
| lh_frontalpole | -0.073 | 0.223 | 0.669 |
| lh_transversetemporal | -0.008 | 0.903 | 0.956 |
| lh_insula | 0.024 | 0.680 | 0.927 |
| rh_bankssts | -0.040 | 0.520 | 0.858 |
| rh_caudalanteriorcingulate | -0.080 | 0.204 | 0.669 |
| rh_caudalmiddlefrontal | -0.099 | 0.106 | 0.582 |
| rh_cuneus | -0.026 | 0.673 | 0.927 |
| rh_entorhinal | 0.038 | 0.548 | 0.861 |
| rh_fusiform | -0.017 | 0.755 | 0.927 |
| rh_inferiorparietal | -0.020 | 0.738 | 0.927 |
| rh_inferiortemporal | -0.028 | 0.620 | 0.927 |
| rh_isthmuscingulate | -0.116 | 0.052 | 0.427 |
| rh_lateraloccipital | -0.035 | 0.546 | 0.861 |
| rh_lateralorbitofrontal | 0.017 | 0.768 | 0.927 |
| rh_lingual | -0.040 | 0.516 | 0.858 |
| rh_medialorbitofrontal | 0.014 | 0.811 | 0.927 |
| rh_middletemporal | -0.042 | 0.467 | 0.858 |
| rh_paracentral | -0.137 | 0.025 | 0.381 |
| rh_parahippocampal | -0.039 | 0.520 | 0.858 |
| rh_parsopercularis | -0.005 | 0.940 | 0.969 |
| rh_parsorbitalis | 0.021 | 0.716 | 0.927 |
| rh_parstriangularis | -0.049 | 0.414 | 0.854 |
| rh_pericalcarine | 0.001 | 0.987 | 0.997 |
| rh_postcentral | -0.042 | 0.470 | 0.858 |
| rh_posteriorcingulate | -0.069 | 0.254 | 0.721 |
| rh_precentral | -0.064 | 0.262 | 0.721 |
| rh_precuneus | -0.073 | 0.205 | 0.669 |
| rh_rostralanteriorcingulate | -0.120 | 0.051 | 0.427 |
| rh_rostralmiddlefrontal | -0.104 | 0.072 | 0.475 |
| rh_superiorfrontal | -0.082 | 0.148 | 0.669 |
| rh_superiorparietal | -0.078 | 0.191 | 0.669 |
| rh_superiortemporal | 0.006 | 0.913 | 0.956 |
| rh_supramarginal | 0.059 | 0.325 | 0.794 |
| rh_frontalpole | -0.128 | 0.034 | 0.381 |
| rh_transversetemporal | 0.016 | 0.793 | 0.927 |
| rh_insula | 0.039 | 0.495 | 0.858 |

The associations betweenOA/OA treatment and brain area were investigated using the multiple linear regression models with the covariates regressed out including age, sex, body mass index, education qualification, smoking status, drinking status, APOE ε4, ethnicity and total physical activity in the UK Biobank.

*P: All P values were calculated after FDR correction.

Abbreviations: CI, Confidence interval; lh, left hemisphere; rh, right hemisphere; FDR, false discovery rate.

# Supplementary Table 20. The association of paracetamol with subsequent dementia outcome in participants.

|  |  | **Model 1**  **(Gender+Age)** | | **Model 2**  **(+APOE ε4 +Ethnic+Education +TPA+BMI+Smoking+Alcohol)** | |  | |
| --- | --- | --- | --- | --- | --- | --- | --- |
|  | **Number of incident dementia/ Number of participants** | **HR (95%CI)** | **P value** | **HR (95%CI)** | **P value** |  |  |
| **ACD** | | | | | |  |  |
| **Exposure elements^a^** |  |  |  |  |  |  |  |
| Nonuse | 640/32151 |  |  |  |  |  |  |
| Drugs^b^ | 388/19738 | **0.865(0.762-0.981)** | **0.024** | **0.862(0.760-0.978)** | **0.021** |  |  |
| Nonuse | 695/35728 |  |  |  |  |  |  |
| NSAIDs^b^ | 333/16161 | 0.899(0.788-1.024) | 0.109 | 0.889(0.780-1.013) | 0.078 |  |  |
| Nonuse | 779/41734 |  |  |  |  |  |  |
| paracet | 249/10155 | 1.004(0.871-1.159) | 0.953 | 0.992(0.860-1.145) | 0.916 |  |  |
| **Lag time** |  |  |  |  |  |  |  |
| Excluding last two years | 222/10128 | 0.896(0.772-1.041) | 0.150 | 0.886(0.763-1.029) | 0.112 |  |  |
| **AD** | | | | | |  |  |
| **Exposure elements** |  |  |  |  |  |  |  |
| Nonuse | 295/31806 |  |  |  |  |  |  |
| Drugs^b^ | 161/19511 | **0.778(0.642-0.942)** | **0.010** | **0.774(0.638-0.938)** | **0.009** |  |  |
| Nonuse | 318/35351 |  |  |  |  |  |  |
| NSAIDs^b^ | 138/15966 | **0.810(0.664-0.990)** | **0.039** | **0.802(0.657-0.980)** | **0.031** |  |  |
| Nonuse | 352/41307 |  |  |  |  |  |  |
| paracet | 104/10010 | 0.915(0.735-1.139) | 0.427 | 0.909 (0.730-1.133) | 0.396 |  |  |
| **Lag time** |  |  |  |  |  |  |  |
| Excluding last two years | 92/9998 | 0.809(0.643-1.018) | 0.071 | 0.803 (0.638-1.011) | 0.062 |  |  |
| **VD** | | | | | |  |  |
| **Exposure elements** |  |  |  |  |  |  |  |
| Nonuse | 231/31652 |  |  |  |  |  |  |
| Drugs^b^ | 105/19455 | 1.048(0.814-1.349) | 0.717 | 1.042(0.809-1.341) | 0.752 |  |  |
| Nonuse | 161/35194 |  |  |  |  |  |  |
| NSAIDs^b^ | 85/15913 | 0.981(0.754-1.276) | 0.884 | 0.966(0.743-1.257) | 0.799 |  |  |
| Nonuse ^b^ | 177/41132 |  |  |  |  |  |  |
| paracet | 69/9975 | 1.212(0.917-1.601) | 0.177 | 1.168(0.883-1.544) | 0.277 |  |  |
| **Lag time** |  |  |  |  |  |  |  |
| Excluding last two years | 60/9966 | 1.053 (0.785-1.413) | 0.729 | 1.016(0.757-1.363) | 0.917 |  |  |

The results are derived from Cox proportional hazard regression models in two models. Bold indicates statistical significance (P value<0.05).

Model 2 was additionally controlled for APOE ε4, ethnic, education, TPA, BMI, smoking and alcohol than model 1.

^a^, the model was based on OA participants, b, the model was based on OA participants.

Abbreviation: OA, osteoarthritis; ACD, all-cause dementia; AD, Alzheimer’s disease; VD, vascular dementia; APOE, Apolipoprotein E; BMI, body mass index; TPA, total physical activity; HR, Hazard ratio; CI, Confidence interval; NSAID, nonsteroidal anti-inflammatory drugs.

# Supplementary figures

Supplementary

## Supplementary Figure 1. Association of NSAID usage in OA patients with incident dementia during follow-up.


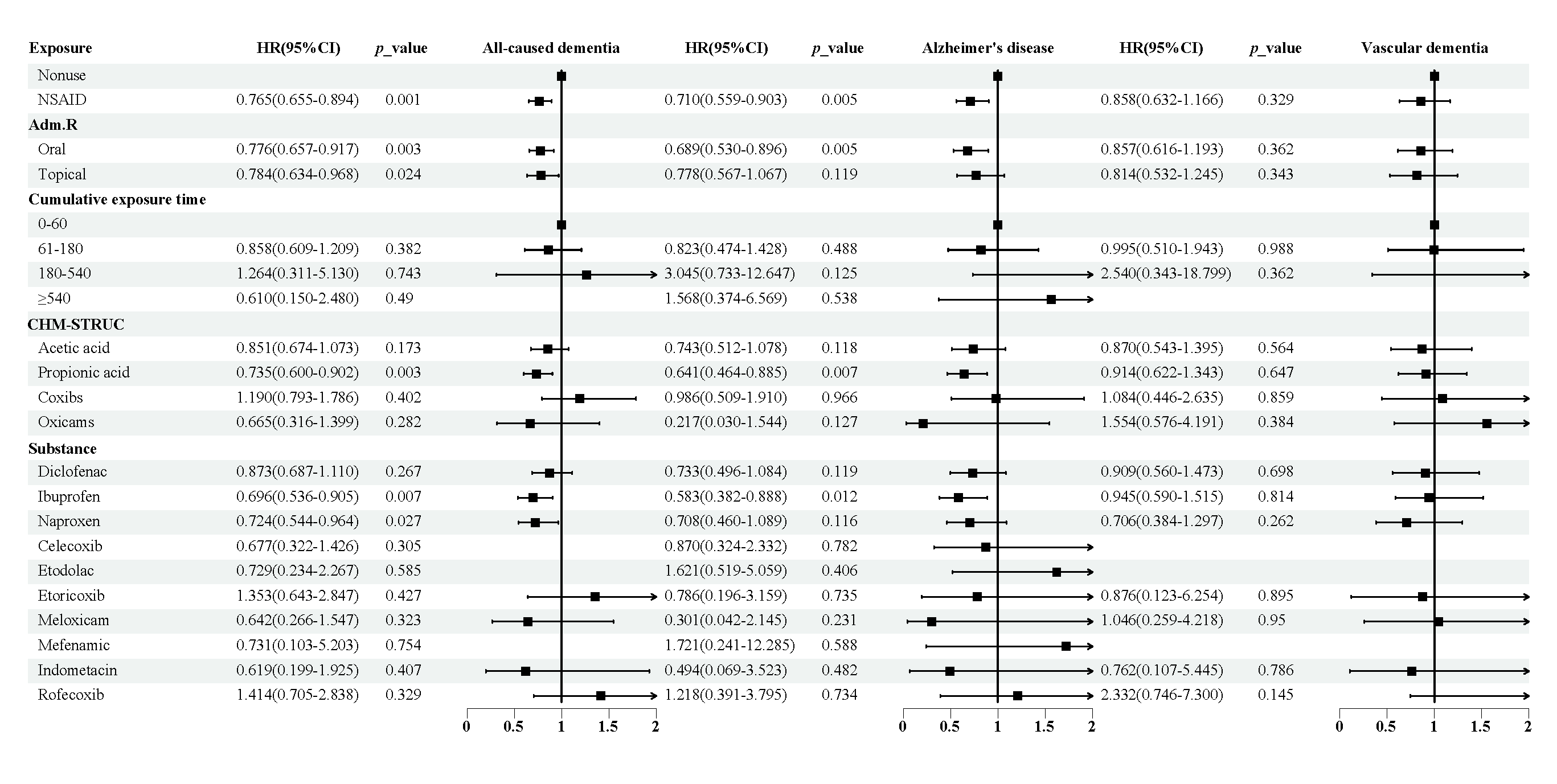


P values were computed by Cox proportional hazard regressions. Covariates included age, sex, ethnicity, education, BMI, TPA, smoking status, alcohol status and APOE ε4 status. After adjusting for all covariates, participants who participants who developed OA, the risk of ACD and AD decreased with NSAIDs, and no statistical significance was found in VD. The black squares represent the hazard ratio (HR) of dementia of developed osteoarthritis group. The black lines indicate the corresponding 95% confidence intervals (CIs) around the HRs.

Abbreviations: ACD, all-cause dementia; AD, Alzheimer’s disease; VD, vascular dementia; HR, hazard ratio; BMI, body mass index; APOE ε4, apolipoprotein E4; TPA, total physical activities; NSAID, nonsteroidal anti-inflammatory drug; Adm.R, route of administration; CHM-STRUC, chemical structure.

## Supplementary Figure 2. Association of opioid usage in OA patients with incident dementia during follow-up.


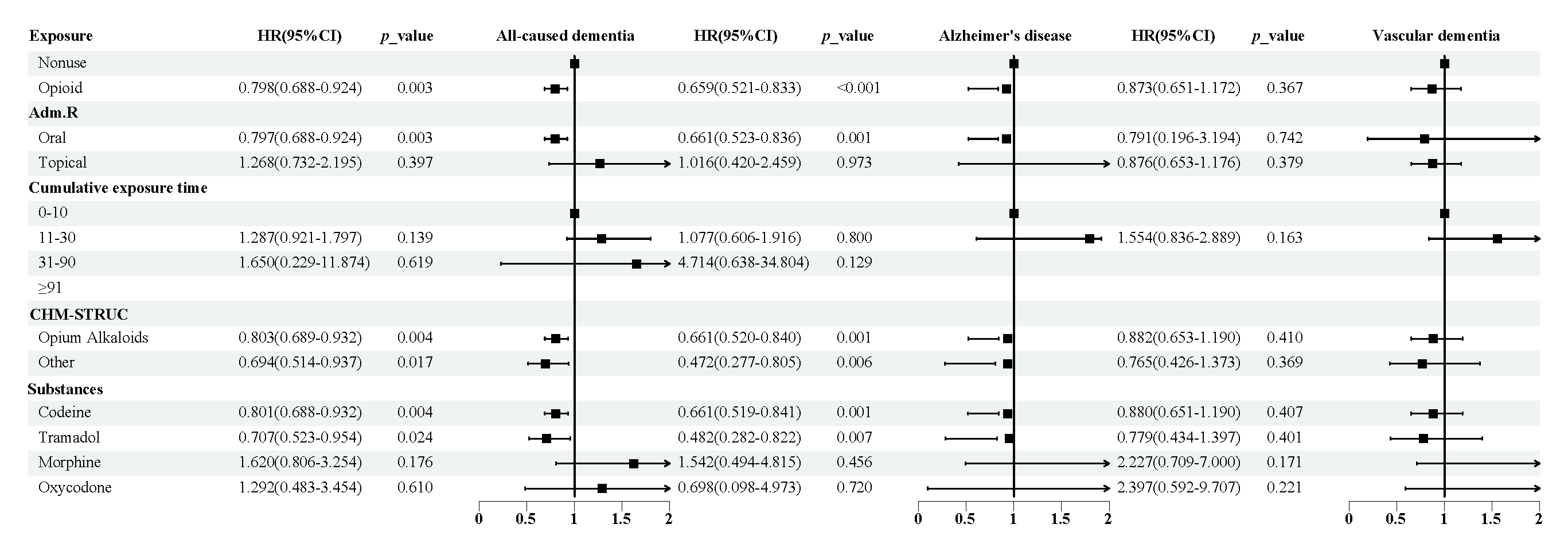


P values were computed by Cox proportional hazard regressions. Covariates included age, sex, ethnicity, education, BMI, TPA, smoking status, alcohol status and APOE ε4 status. After adjusting for all covariates, participants who developed OA, the risk of ACD and AD decreased with opioid, and no statistical significance was found in VD. The black squares represent the hazard ratio (HR) of dementia of developed osteoarthritis group. The black lines indicate the corresponding 95% confidence intervals (CIs) around the HRs.

Abbreviations: ACD, all-cause dementia; AD, Alzheimer’s disease; VD, vascular dementia; HR, hazard ratio; BMI, body mass index; APOE ε4, apolipoprotein E4; TPA, total physical activities; Adm.R, route of administration; CHM-STRUC, chemical structure.

## Supplementary Figure 3. Association between brain structure with OA and OA treatment.


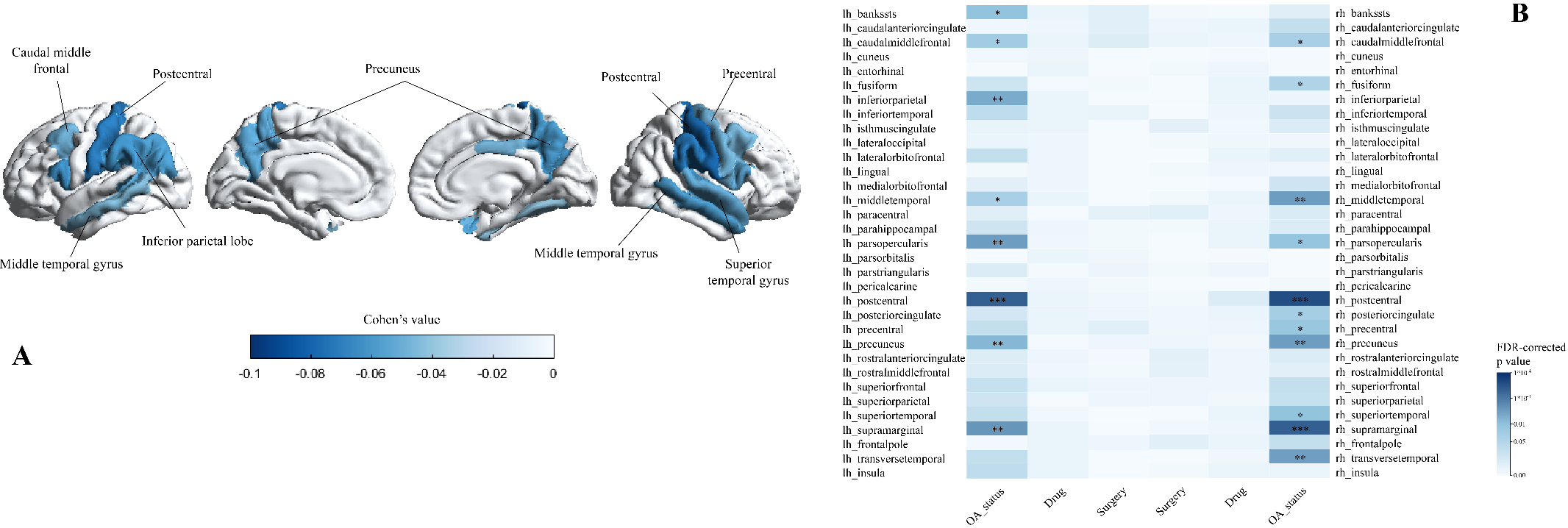


Supplementary Figure 3A displays that cortical area significantly associated with osteoarthritis (FDR-corrected p < 0.05). Here, we only show the regions with Cohen’s value <0. The heatmap (3B), the FDR-corrected p values for the associations between OA/OA treatment and brain structure.

Abbreviations: OA, osteoarthritis; lh, left hemisphere; rh, right hemisphere; FDR, false discovery rate.

# Reference

1. Yang L, Deng YT, Leng Y, Ou YN, Li YZ, Chen SD, et al. Depression, Depression Treatments, and Risk of Incident Dementia: A Prospective Cohort Study of 354,313 Participants. Biol Psychiatry. 2023 May 1;93(9):802-9.

2. Horwitz RI, Feinstein AR. The problem of "protopathic bias" in case-control studies. The American journal of medicine. 1980 Feb;68(2):255-8.

3. Bannuru RR, Schmid CH, Kent DM, Vaysbrot EE, Wong JB, McAlindon TE. Comparative effectiveness of pharmacologic interventions for knee osteoarthritis: a systematic review and network meta-analysis. Ann Intern Med. 2015 Jan 6;162(1):46-54.

4. Zhu X, Wu D, Sang L, Wang Y, Shen Y, Zhuang X, et al. Comparative effectiveness of glucosamine, chondroitin, acetaminophen or celecoxib for the treatment of knee and/or hip osteoarthritis: a network meta-analysis. Clin Exp Rheumatol. 2018 Jul-Aug;36(4):595-602.

5. Wluka AE, Ding C, Wang Y, Jones G, Urquhart DM, Cicuttini FM. Aspirin is associated with reduced cartilage loss in knee osteoarthritis: Data from a cohort study. Maturitas. 2015 Jul;81(3):394-7.

6. Toupin April K, Bisaillon J, Welch V, Maxwell LJ, Jüni P, Rutjes AW, et al. Tramadol for osteoarthritis. Cochrane Database Syst Rev. 2019 May 27;5(5):Cd005522.

7. Fuggle N, Curtis E, Shaw S, Spooner L, Bruyère O, Ntani G, et al. Safety of Opioids in Osteoarthritis: Outcomes of a Systematic Review and Meta-Analysis. Drugs Aging. 2019 Apr;36(Suppl 1):129-43.

8. Beard CM, Waring SC, O'Brien PC, Kurland LT, Kokmen E. Nonsteroidal anti-inflammatory drug use and Alzheimer's disease: a case-control study in Rochester, Minnesota, 1980 through 1984. Mayo Clin Proc. 1998 Oct;73(10):951-5.

9. Xue Y-H, Peng Y-S, Ting H-F, Hsieh JP, Huang Y-K, Wang Y-H, et al. Etoricoxib and Diclofenac Might Reduce the Risk of Dementia in Patients with Osteoarthritis: A Nation-Wide, Population-Based Retrospective Cohort Study. Dementia and Geriatric Cognitive Disorders. 2018 2018;45(5-6):262-71.

10. Organization. WH. Anatomical therapeutic classification (ATC) index including defined daily doses (DDD) for plain substances.; Available from: <https://www.whocc.no/ddd/definition_and_general_considera/>.

11. in t' Veld BA, Ruitenberg A, Hofman A, Launer LJ, van Duijn CM, Stijnen T, et al. Nonsteroidal antiinflammatory drugs and the risk of Alzheimer's disease. N Engl J Med. 2001 Nov 22;345(21):1515-21.

12. Gray SL, Anderson ML, Dublin S, Hanlon JT, Hubbard R, Walker R, et al. Cumulative use of strong anticholinergics and incident dementia: a prospective cohort study. JAMA Intern Med. 2015 Mar;175(3):401-7.

13. Dublin S, Walker RL, Gray SL, Hubbard RA, Anderson ML, Yu O, et al. Prescription Opioids and Risk of Dementia or Cognitive Decline: A Prospective Cohort Study. J Am Geriatr Soc. 2015 Aug;63(8):1519-26.

14. Gallagher B, Tjoumakaris FP, Harwood MI, Good RP, Ciccotti MG, Freedman KB. Chondroprotection and the prevention of osteoarthritis progression of the knee: a systematic review of treatment agents. Am J Sports Med. 2015 Mar;43(3):734-44.

15. Richy F, Bruyere O, Ethgen O, Cucherat M, Henrotin Y, Reginster JY. Structural and symptomatic efficacy of glucosamine and chondroitin in knee osteoarthritis: a comprehensive meta-analysis. Arch Intern Med. 2003 Jul 14;163(13):1514-22.

16. Wandel S, Jüni P, Tendal B, Nüesch E, Villiger PM, Welton NJ, et al. Effects of glucosamine, chondroitin, or placebo in patients with osteoarthritis of hip or knee: network meta-analysis. Bmj. 2010 Sep 16;341:c4675.

17. Ayhan E, Kesmezacar H, Akgun I. Intraarticular injections (corticosteroid, hyaluronic acid, platelet rich plasma) for the knee osteoarthritis. World J Orthop. 2014 Jul 18;5(3):351-61.

18. Kolasinski SL, Neogi T, Hochberg MC, Oatis C, Guyatt G, Block J, et al. 2019 American College of Rheumatology/Arthritis Foundation Guideline for the Management of Osteoarthritis of the Hand, Hip, and Knee. Arthritis Care Res (Hoboken). 2020 Feb;72(2):149-62.

19. O'Connor D, Johnston RV, Brignardello-Petersen R, Poolman RW, Cyril S, Vandvik PO, et al. Arthroscopic surgery for degenerative knee disease (osteoarthritis including degenerative meniscal tears). Cochrane Database Syst Rev. 2022 Mar 3;3(3):Cd014328.

20. Chen L, Ferreira ML, Nassar N, Preen DB, Hopper JL, Li S, et al. Association of chronic musculoskeletal pain with mortality among UK adults: A population-based cohort study with mediation analysis. EClinicalMedicine. 2021 Dec;42:101202.

21. Zhu J, Chen W, Hu Y, Qu Y, Yang H, Zeng Y, et al. Physical activity patterns, genetic susceptibility, and risk of hip/knee osteoarthritis: a prospective cohort study based on the UK Biobank. Osteoarthritis Cartilage. 2022 Aug;30(8):1079-90.

22. Knevel R, le Cessie S, Terao CC, Slowikowski K, Cui J, Huizinga TWJ, et al. Using genetics to prioritize diagnoses for rheumatology outpatients with inflammatory arthritis. Sci Transl Med. 2020 May 27;12(545).

23. Wu X. Innate Lymphocytes in Inflammatory Arthritis. Front Immunol. 2020;11:565275.

24. Gullick NJ, Scott DL. Drug therapy of inflammatory arthritis. Clin Med (Lond). 2012 Aug;12(4):357-63.
